# Rapid outbreak sequencing of Ebola virus in Sierra Leone identifies transmission chains linked to sporadic cases

**DOI:** 10.1093/ve/vew016

**Published:** 2016-06-22

**Authors:** Armando Arias, Simon J. Watson, Danny Asogun, Ekaete Alice Tobin, Jia Lu, My V. T. Phan, Umaru Jah, Raoul Emeric Guetiya Wadoum, Luke Meredith, Lucy Thorne, Sarah Caddy, Alimamy Tarawalie, Pinky Langat, Gytis Dudas, Nuno R. Faria, Simon Dellicour, Abdul Kamara, Brima Kargbo, Brima Osaio Kamara, Sahr Gevao, Daniel Cooper, Matthew Newport, Peter Horby, Jake Dunning, Foday Sahr, Tim Brooks, Andrew J.H. Simpson, Elisabetta Groppelli, Guoying Liu, Nisha Mulakken, Kate Rhodes, James Akpablie, Zabulon Yoti, Margaret Lamunu, Esther Vitto, Patrick Otim, Collins Owilli, Isaac Boateng, Lawrence Okoror, Emmanuel Omomoh, Jennifer Oyakhilome, Racheal Omiunu, Ighodalo Yemisis, Donatus Adomeh, Solomon Ehikhiametalor, Patience Akhilomen, Chris Aire, Andreas Kurth, Nicola Cook, Jan Baumann, Martin Gabriel, Roman Wölfel, Antonino Di Caro, Miles W. Carroll, Stephan Günther, John Redd, Dhamari Naidoo, Oliver G. Pybus, Andrew Rambaut, Paul Kellam, Ian Goodfellow, Matthew Cotten

**Affiliations:** ^1^ Division of Virology, Department of Pathology, University of Cambridge, Cambridge, United Kingdom; ^2^ Wellcome Trust Sanger Institute, Hinxton, United Kingdom; ^3^ Irrua Specialist Teaching Hospital, Institute of Lassa Fever Research and Control, Irrua, Nigeria; ^4^ The European Mobile Laboratory Consortium, Bernhard Nocht Institute for Tropical Medicine, Hamburg, Germany; ^5^ University of Makeni, Makeni, Sierra Leone; ^6^ Institute of Evolutionary Biology, Ashworth Laboratories, Edinburgh, United Kingdom; ^7^ Department of Zoology, University of Oxford, Oxford, UK; ^8^ Sierra Leone Ministry of Health, Freetown, Sierra Leone; ^9^ International Medical Corps, Los Angeles, CA, USA; ^10^ Department of Medicine, Epidemic Diseases Research Group Oxford (ERGO), Centre for Tropical Medicine and Global Health Nuffield, University of Oxford, Oxford, United Kingdom; ^11^ Republic of Sierra Leone Armed Forces, Freetown, Sierra Leone; ^12^ Rare and Imported Pathogens Laboratory, Public Health England, United Kingdom; ^13^ Thermo Fisher Scientific, South San Francisco, CA, USA; ^14^ WHO Ebola Response Team, Geneva, Switzerland; ^15^ Federal University, Oye-Ekit, Nigeria; ^16^ Robert Koch Institute, Berlin, Germany; ^17^ Public Health England, Porton Down, United Kingdom; ^18^ Bernhard Nocht Institute for Tropical Medicine, Hamburg, Germany; ^19^ Bundeswehr Institute of Microbiology, Munich, Germany; ^20^ National Institute for Infectious Diseases “L. Spallanzani”, Rome, Italy; ^21^ Sierra Leone and Division of Global Health Protection, CDC Country Office, Georgia Center for Global Health Centers for Disease Control and Prevention, Atlanta, GA, USA; ^22^ Fogarty International Center, NIH, Bethesda, MD, USA; ^23^ Infection and Evolution, Centre for Immunology, Ashworth Laboratories, Edinburgh, United Kingdom; ^24^ Division of Infection and Immunity, University College London, London, United Kingdom

**Keywords:** Ebola virus, evolution, transmission, outbreak sequencing

## Abstract

To end the largest known outbreak of Ebola virus disease (EVD) in West Africa and to prevent new transmissions, rapid epidemiological tracing of cases and contacts was required. The ability to quickly identify unknown sources and chains of transmission is key to ending the EVD epidemic and of even greater importance in the context of recent reports of Ebola virus (EBOV) persistence in survivors. Phylogenetic analysis of complete EBOV genomes can provide important information on the source of any new infection. A local deep sequencing facility was established at the Mateneh Ebola Treatment Centre in central Sierra Leone. The facility included all wetlab and computational resources to rapidly process EBOV diagnostic samples into full genome sequences. We produced 554 EBOV genomes from EVD cases across Sierra Leone. These genomes provided a detailed description of EBOV evolution and facilitated phylogenetic tracking of new EVD cases. Importantly, we show that linked genomic and epidemiological data can not only support contact tracing but also identify unconventional transmission chains involving body fluids, including semen. Rapid EBOV genome sequencing, when linked to epidemiological information and a comprehensive database of virus sequences across the outbreak, provided a powerful tool for public health epidemic control efforts.

## 1. Introduction

Starting in December 2013, West Africa experienced the largest known outbreak of Ebola virus disease (EVD). Sierra Leone was the most widely affected country, with 14,124 cases and 3,956 confirmed deaths as of 21 February 2016 (WHO 2016). In the absence of large-scale vaccination and effective antiviral drugs, controlling the epidemic and maintaining the zero transmission status have relied on rapid patient identification and isolation, contact tracing and quarantine, as well as the implementation of safe burial practices (Kucharski et al. 2015; Nouvellet et al. 2015; Fang et al. 2016).

By January 2015, the decline in new cases in the three most-affected countries (Sierra Leone, Guinea, and Liberia) suggested that epidemiological containment efforts were succeeding, particularly in Liberia which was initially declared free of EVD by the WHO on 9 May 2015 (WHO 2015). However, the recurrence of EVD in Liberia (WHO 2015) and Sierra Leone (WHO 2016) indicated that sources of new infections remained; even after all recognized chains of transmission had been extinguished. Worryingly, evidence is accumulating that EVD survivors may harbor and transmit EBOV for several months after recovery (Deen et al. 2015; Christie et al. 2015; Mate et al. 2015; Blackley et al. 2016; Sow et al. 2016; Uyeki et al. 2016) raising the possibility that transmission through exposure to bodily fluids and/or sexual transmission can occur at times beyond the standard quarantine periods.

To facilitate the use of phylogenetics for tracing virus transmission, a local EBOV sequencing facility was established in a tent at the Ebola Treatment Centre in Makeni, Sierra Leone. The facility provided local capacity for rapid real-time sequencing of EBOV genomes directly from clinical samples and contributed important information on the transmission pathways of EBOV.

## 2. Methods

### 2.1. Samples

**Figure 1.**
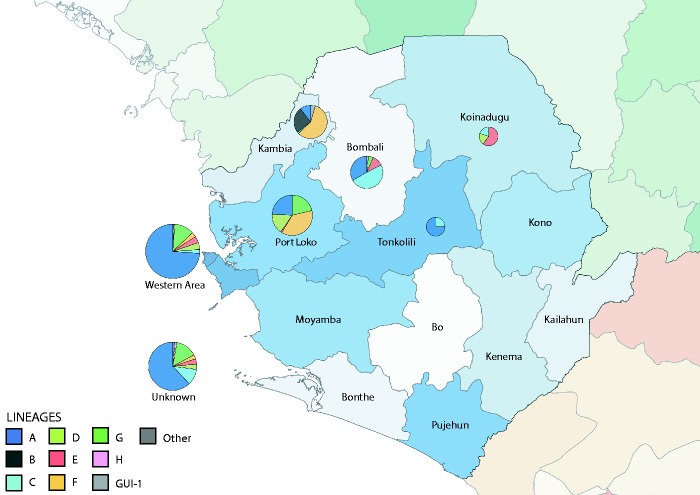
Lineages circulating in sampled regions. Districts of Sierra Leone (blue), Guinea (green), and Liberia (orange) are indicated. Pie charts are drawn over districts from which samples of this study were collected, with size relative to the number of samples, and segment area indicating the proportion of lineages (as defined in Figure 2) observed at that location. The number of genomes from each location was the following: Bombali: 63, Kambia: 67, Koinadugu: 5, Port Loko: 98, Tonkolili: 4, Western Area: 182, Unknown location: 135.

Samples were collected from patients being cared for in Ebola isolation and treatment centers in Makeni (Bombali district), Port Loko (Port Loko district), Kambia district, Kerrytown (Western Urban district), and Koinadugu district (see Fig. 1, sample details are summarized in Supplementary Table 1). The study was conducted in compliance with principles expressed in the Declaration of Helsinki, and ethical approvals for the use of residual diagnostic samples for sequencing were obtained from the Sierra Leone Ethics and Scientific Review Committee and the Ministry of Health of Sierra Leone. The Sierra Leone Ethics and Scientific Review Committee approved the use of diagnostic leftover samples collected by EMLab and corresponding patient data for this study.

### 2.2. Logistics

Equipment and reagents for the establishment of the sequencing facility were initially shipped to the University of Cambridge, Cambridge, UK, for testing and repacking prior to transport to Makeni, Sierra Leone. These materials included reagents for sequencing, unassembled benches, PCR cabinets, centrifuges, general molecular biology reagents, N_2_ canisters (required for Ion Torrent sequencing), and the equipment required to perform the sequencing workflow, namely an Ion Chef liquid handling robot and an Ion Torrent PGM sequencer. The Ion Torrent PGM sequencer and Chef were unpacked, installed and tested in Cambridge by the users with the aid of a Thermo Fisher Scientific engineer. Calibration sequencing runs were performed to ensure the required reagents and equipment functioned correctly, prior to repacking and transfer to East Midlands Airport for transport to Makeni via UK Department for International Development-funded humanitarian aid flights. The equipment arrived in Makeni on 15 April 2015 and was installed in a lined, air-conditioned tent in the Mateneh Ebola treatment centre (ETC) in Makeni, Bombali district, adjacent to the Public Health England (PHE) operated diagnostic facility. The sequencing facility was operational from 16 April 2015 and the first data files were transferred to the UK on 20 April 2015.

### 2.3. Sample preparation and sequencing

Total nucleic acid extracts were prepared from plasma obtained from collected blood samples or buccal swabs using either the Qiagen EZ-1 automated nucleic acid purification platform or the QIAamp manual RNA extraction procedure. Samples were tested for the presence of EBOV RNA using as previously described (Trombley et al. 2010) and were considered positive if Ct values were <40. Nucleic acid extracts from EBOV PCR-positive samples were then subjected to reverse transcription/PCR amplification using the Thermo Fisher Scientific Ion Ampliseq workflow according to the protocol manufacturer with EBOV specific reagents and the Ion Torrent sequencing platform. Following nucleic acid isolation, all subsequent procedures were performed within physically separated PCR cabinets dedicated for either reagent preparation or sample manipulations, with a 30 min UV treatment cycle between uses. Briefly, 5–7 µl of nucleic acid extract were reverse transcribed using the VILO reverse transcriptase kit (Life Technologies) in a total volume of 10 µl. Following reverse transcription, PCR amplification of the EBOV cDNA was performed with two multiplex PCR reactions: pool 1 containing 73 EBOV-specific primer pairs and five human housekeeping gene controls and pool 2 containing 72 EBOV-specific primer pairs and the same five human housekeeping gene controls. The amplicon sizes range from 80 to 237 bp (see Supplementary Table 2 for primer sequences and mapping positions). Following PCR amplification, primer sequences were removed from the amplicons and barcoded adapters ligated according to the protocol of the manufacturer. Amplicon purification and size selection were performed with the AMPure DNA purification system, followed by library quantification by qPCR using the Ion Library Quantitation Kit. Libraries were normalized to 85 pM, combined in pools of 10–24 samples per pool and template libraries were prepared using the Ion PGM Hi-Q Sequencing Kit on an Ion Chef Instrument (Thermo Fisher Scientific). Libraries were subsequently sequenced on the Ion PGM System using Ion Torrent Hi-Q sequencing reagents (500 cycles).

### 2.4. Data handling and genomes assembly

Short read sets were processed to remove short and low quality reads, terminal primers were removed and the reads were sorted to retain reads with length >125 nt and median Phred score > 30 using QUASR (Watson et al. 2013). Chimeric reads were resolved using a Python script and the final reads were processed by *de novo* assembly using SPAdes 3.5.0 (Bankevich et al. 2012). EBOV contigs were further assembled into complete genomes (if not already complete) using Sequencher v5.3 (Gene Codes Corporation, USA). Conflicts were resolved by direct counting of the motif in the short read data set. Further details of the genome assembly process are included in the Supplementary material.

### 2.5. Phylogenetic methods

All available EBOV Makona genomes were downloaded from the NCBI Ebolavirus Resource (NCBI 2016). These 1019 genomes were combined with the 554 new genomes generated here, and aligned manually using the AliView alignment editor (Larsson 2014). A maximum-likelihood phylogenetic tree was inferred from this alignment using RAxML version 7.8.6 (Stamatakis 2014) under a general time reversible (GTR) substitution model, with among-site heterogeneity modelled using a 4-category discrete approximation of a gamma distribution, as previously described (Gire et al. 2014; Ladner et al. 2015). Robustness of the tree topology was assessed by bootstrap analysis of 1,000 pseudo-replicates, with support values for the topology calculated using the SumTrees program version 4.0.0 of the DendroPy package version 4.0.0 (Sukumaran and Holder 2010). The tree was rooted on the Gueckedou-C05 genome (GenBank accession no. KJ660348) and visualised using FigTree version 1.4.2 (http://tree.bio.ed.ac.uk/software/figtree/).

From this tree, the well-supported clades were identified, including the previously determined SL3 introduction into Sierra Leone. Viruses derived from the SL3 introduction that were isolated in Sierra Leone were extracted from the alignment. These did not include those that were derived from a re-importation of the virus from another country (e.g. Lineage B, which was derived from a reintroduction from Guinea). A molecular clock phylogenetic tree was inferred from these 1058 genomes using a Bayesian Markov chain Monte Carlo (MCMC) approach implemented in BEAST version 1.8.2 (Drummond et al. 2012). The alignment was partitioned into a concatenated coding region, containing the protein-coding sequences of the *NP*, *vp35*, *vp40*, *GP1*, *GP2*, *vp30*, *vp24*, and L genes, and a non-coding inter-genic region. The coding region was modeled under an SRD06 substitution model (Shapiro et al. 2006) to allow for partitioning of codon positions 1 + 2 and 3, while the inter-genic region was modeled under an HKY + Γ4 substitution model (Hasegawa et al. 1985), as previously applied for molecular dating of EBOV (Gire et al. 2014). The data were run under an uncorrelated lognormal relaxed molecular clock (Drummond et al. 2006), and a non-parametric Bayesian Skygrid coalescent model (Gill et al. 2013). Ten independent chains were run for a combined total of at least 30 million states, then combined after burn-in. Burn-in values were determined for each chain separately after checking for convergence using Tracer version 1.6 (http://tree.bio.ed.ac.uk/software/tracer/). The posterior tree sets were combined using LogCombiner version 1.8.2, then summarised as a maximum clade credibility tree using TreeAnnotator version 1.8.2. This tree was visualised using FigTree version 1.4.2.

## 3. Results and discussion

We produced 554 contemporary EBOV genome sequences from 855 EVD samples (64% success rate) collected in Sierra Leone between December 2014 and September 2015. PCR-positive EBOV samples were provided by EBOV diagnostic field laboratories (PHE Makeni, PHE Port Loko, PHE Kerrytown, EML Hastings, EML Kambia), collected primarily from the northern and western districts of Sierra Leone (Fig. 1, Supplementary Table 1), reflecting EVD case locations during this period (WHO 2016). Genomes were successfully obtained from blood, buccal swabs, semen and breast milk with successful genome yield dependent on EBOV reads of greater than 10,000 (Supplementary Fig. 1). The sequenced genomes represent 4.5% of the EVD cases reported for Sierra Leone, and 23.8% of all 2015 Sierra Leone cases (see Supplementary Fig. 2) and provide a detailed description of EBOV evolution during 2015. From these data we identified sources of infection for some of the final EVD cases in Sierra Leone and indicate potential routes of sexual and breast milk transmissions.

This was an unconventional use of new sequencing technology under harsh conditions (high temperature, dust, high humidity, unreliable power supplies, complicated reagent transport, in a tent). Accordingly, special care was taken to ensure that the sequencing process was reproducible and consistent with EBOV sequencing results obtained by other groups. Furthermore, we provided quantitative data on the level of residual primer content from the amplicon sequencing method and the potential level of sample cross contamination under the sequencing conditions used (see Supplementary material).

**Figure 2.**
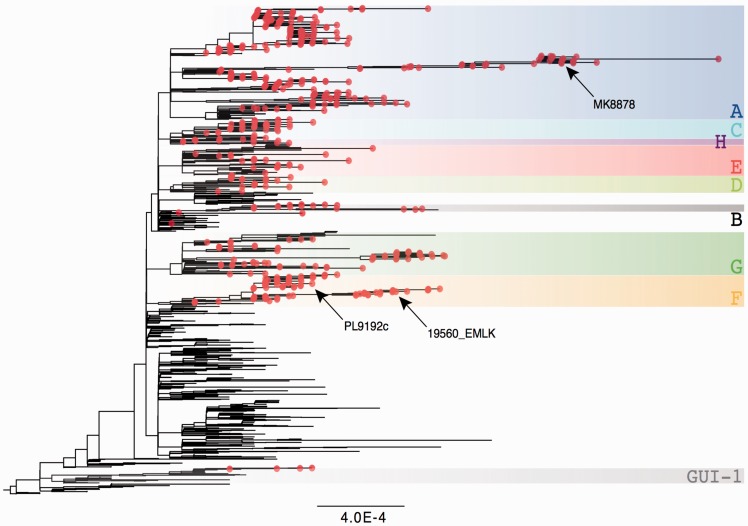
Maximum-likelihood tree showing the phylogenetic context of the viruses sequenced in this study. The 554 genomes generated here are shown as red circles, while the nine comprising lineages are highlighted with colored boxes and labeled A–H for those derived from the SL3 lineage, or GUI-1 for viruses derived from the divergent Guinean lineage. The tree was rooted on Gueckedou-C05 (GenBank accession no. KJ660348), with the scale bar indicating genetic distance in units of substitutions/site. Specific genomes in the three transmission vignettes (see Fig. 3), MK8878, 19560_EMLK, and PL9192c are highlighted.

Evolutionary analysis of the complete set of EBOV Makona genomes revealed that at least nine viral lineages were circulating in Sierra Leone (Fig. 2). Eight of these lineages (A–H) were derived from the SL3 variant that emerged in Sierra Leone in June 2014 (Park et al. 2015) and became the most prevalent lineage (Tong et al. 2015). The remaining viruses were derived from a separate introduction into Sierra Leone of the GUI-1 lineage from Guinea (Simon-Loriere et al. 2015). By June 2015, reported EVD cases were from infections by only three viral lineages A, E, and F (Supplementary Fig. 3). The majority of these cases arose from two separate outbreaks: one with lineage F viruses that occurred primarily in the Port Loko and Kambia districts (80 genomes), and the other from lineage A viruses that were identified primarily in the Magazine Wharf area of Freetown in the Western Urban district (39 genomes). Both these outbreaks persisted for over a month, with the phylogenetic analyses revealing movement of the virus to surrounding districts. This virus movement was observed across the entire Sierra Leone outbreak, with viruses from all lineages except B and C found in more than one district (Supplementary Fig. 3).

The Ebola Outbreak Sequencing Support (EOSS) was established in July 2015 as a coordinated effort from the Sierra Leone Ministry of Health, WHO, CDC and the local sequencing facility to rapidly sequence all new Sierra Leone EVD cases and rapidly place them in phylogenetic context. EOSS processed 21 samples from July-September 2015 (median 4 days, range 1–12 days, Supplementary Fig. 4) and provided an additional level of information to field workers tracing the source of the infection. Three examples of the use of these sequence data follow.

**Figure 3.**
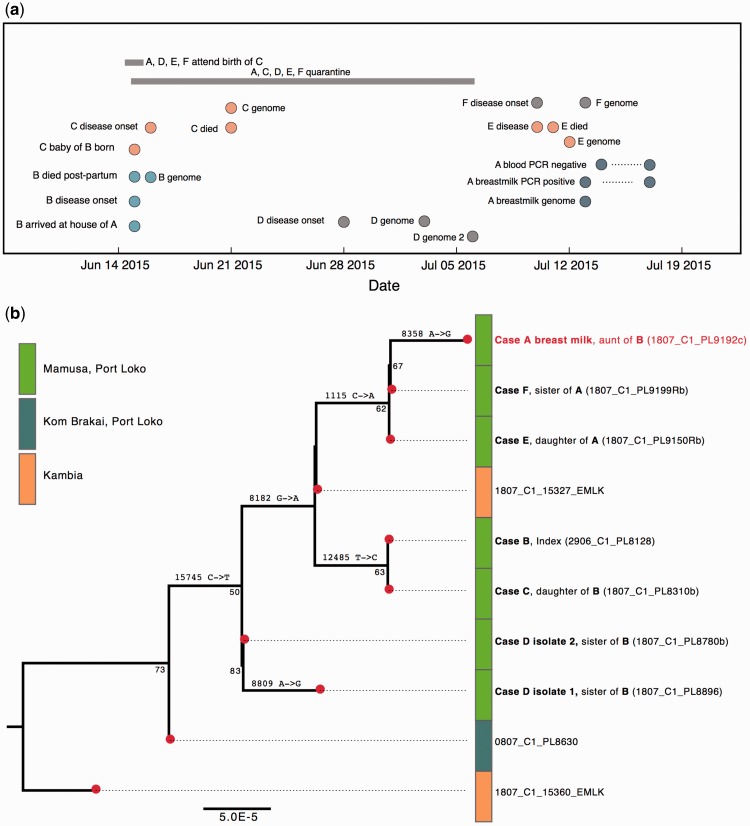
(a) Mamusa Cluster timeline. Key events in the Mamusa cluster examined in (b) are summarized. (b) Maximum-likelihood tree of the Mamusa cluster showing the phylogenetic relationship between each case's virus genome. The genome from the case *A* breast milk sample (PL9192, labeled in red, GenBank accession no. KU296401) is highlighted in red. Additional cases in the cluster include the earlier case *B* (most probable index case of the cluster, GenBank accession no. KU296340); case *C* (the 6 day-old newborn daughter of *B*, GenBank accession no. KU296618), and *D* (sister of *B*, includes two viruses sampled 3 d apart, GenBank accession nos. KU296404 and KU296342). Contacts of *A* include cases *E* (13 month-old daughter of *A*, GenBank accession no. KU296522) and *F* (sister of *A*, GenBank accession no. KU296371). Bootstrap support values greater than 50% are given below the respective node. The bar colors on the right indicate the place of sampling of each virus (legend is shown on the left). All mutations within the case cluster are given above the relevant branch as the position in the original alignment followed by the nucleotide change. The scale bar indicates the genetic distance in units of substitutions/site.

An EVD cluster occurred in late June 2015 in Mamusa, Port Loko District. Case *B*, who was in the late stages of pregnancy, had been exposed to EVD in another village (Kom Brakai) and was under quarantine there. She fled quarantine and traveled to the house of her aunt (case *A*) in Mamusa (Fig. 3a). Case *B* went into labour, and died on 15 June during the birth of case *C*. Cases *B* and *C* were subsequently found to be EBOV positive. Consequently, all household contacts present at *C*’s birth were placed in quarantine, including cases *A*, *C* (*B*'s newborn daughter who died on 25 June), *D* (*B*’s sister), *E* (*A*’s 13-month old daughter), and *F* (*A*’s sister). Cases *A*, *E*, and *F* were released from quarantine on 7 July after completing their observation period without apparent illness other than red conjunctivae noted in *A* on 29 June, although no EBOV diagnostics were performed before release. Cases *E* and *F* subsequently developed symptoms of EVD on 10 July, 3 d after completing quarantine (see timeline, Fig. 3a), prompting evaluation of *A*, who remained asymptomatic. Although a blood sample from *A* was EBOV-negative on 17 July, two samples of her breast milk were EBOV-positive on 13 and 17 July (Fig. 3a). The full EBOV genome obtained from *A*’s breast milk (PL9192) was found to phylogenetically cluster with genomes from *E* (PL9150Rb) and *F* (PL9199Rb) (Fig. 3b). This cluster is strongly supported and is distinct from genomes from the earlier cases *B, C*, and *D*. We hypothesized three possible routes by which E was infected:

*Route* 1: *A*, *E*, and *F* were infected while attending *C*’s birth by direct contact with *B* or *C*.

Route 2: *A* was infected while attending C’s birth. *A* transmitted the virus to *E*, through breastfeeding or direct contact; the virus was subsequently transmitted onward to *F* during quarantine due to close proximity of *F* with *A* or *E*.

Route 3: *A*, *E*, and *F* were infected by exposure to *C* or *D* during the quarantine.

If Route 1 or 3 were correct, the viruses isolated from *A, E*, and *F* would be more closely related to and cluster with viruses isolated from cases *B, C*, and *D*.

However, the viral genome isolated from *B* and the two genomes from *D* bear distinct nucleotide changes (12,485 T->C and 8,182 A->G), that were not in the genomes of viruses obtained from cases *A, E*, and *F*, with no evidence of mixed infections at these genome sites (results not show), suggesting a separate transmission chain. Based on these data, we therefore, concluded that transmission scenarios Routes 1 and 3 were less likely.

Although A’s viral genome contains a unique mutation (A8358G) not shared by any other virus, analysis of *A*’s viral reads shows that this was a polymorphic position with 65% of the reads having the G, and 35% containing the *A*. Therefore, as cases *A*, *E*, and *F* have evidence for identical viruses, and they all share a unique mutation (C1115A), they are likely to either all share a common direct ancestor (likely *B*, *C*, or *D* given the timings and locations) or one case gave rise to the others (e.g. case *A* was infected by *B/C/D* and transmitted to *E* and *F*) and the data best support Route 2.

It is important to note that given the practical difficulties of obtaining multiple samples from EVD patients and that the primary priority of field workers at that time was to contain the epidemic, further sampling of community members and additional body compartments and fluids was not performed, which could have provided clarification of the transmission route. The two EBOV-positive breast milk samples from *A*, and the fact that *E* was actively breastfed by *A* during the quarantine period, support the possibility of breast milk transmission. However, *A* and *E* also had close contact other than breastfeeding, and the lack of an earlier blood sample from *A* does not allow us to prove that transmission occurred via breast milk. Similar complexities of drawing conclusions about EBOV breast milk transmission have been reported (Moreau et al. 2015; Nordenstedt et al. 2016).

**Figure 4.**
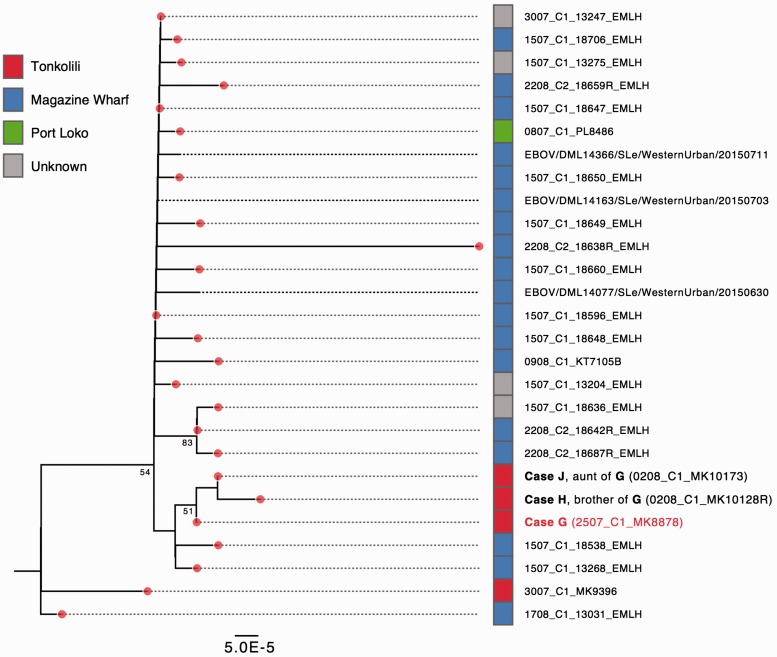
Maximum-likelihood tree showing that the Tonkolili case derived from the Magazine Wharf lineage. The Tonkolili index case *G* (MK8878, labeled in red, GenBank accession no. KU296684) was derived from a clade of viruses circulating predominantly in Magazine Wharf, and clusters with the two secondary Tonkolili secondary cases *H* (*G*’s brother, GenBank accession no. KU296502) and *J* (*G*’s aunt, GenBank accession no. KU296313). See legend of Fig. 3(a) for additional figure details.

In a second cluster, on 24 July 2015, EVD case *G* was identified in a village in Tonkolili district which had been EVD-free for the previous 130 d. However, at that time, there were only three locations in Sierra Leone with on-going EBOV transmission (Magazine Wharf in Freetown, Kambia and Port Loko) in addition to cases in Guinea. Case *G* reported travel from Freetown to Tonkolili on 16 July 2015, providing a hypothesis for EBOV appearance in Tonkolili. Phylogenetic analysis confirmed this hypothesis; the virus genome from *G* (MK8878) clustered with recent infections from Magazine Wharf and *not* with viruses from the other locations with active transmission at the time (Fig. 4 and Supplementary Fig. 3). Furthermore, genomes from two subsequent EVD cases from Tonkolili, *H* (MK10128; *G's* brother), and *J* (MK10173; *G's* aunt), both *G’s* caregivers, were closely related to the *G* genome expanding the transmission chain (Fig. 4). The combined data link case *G* to known infections in Magazine Wharf and exclude the possibility that this Tonkolili cluster was a re-emergence of EBOV from previous Tonkolili cases or from an unknown transmission chain.

**Figure 5.**
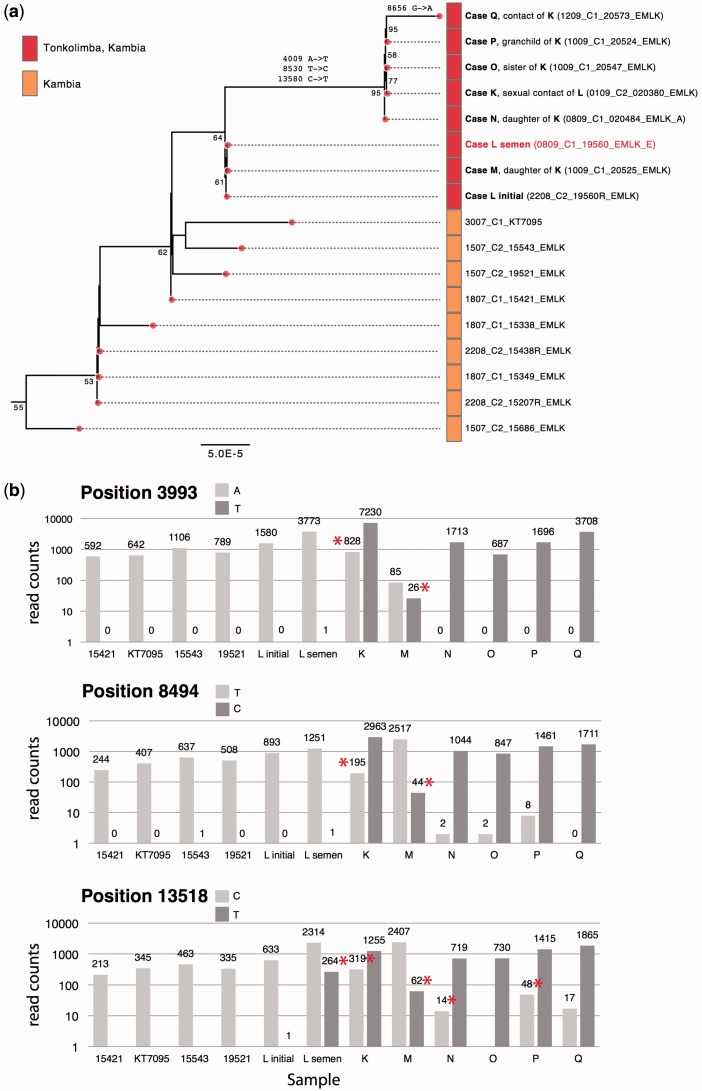
(a) Maximum-likelihood tree showing the Kambia cluster with possible sexual transmission and full genome from a semen sample. The virus from case *L*’s initial acute sample (19560R_EMLK, GenBank accession no. KU296580) is the most probable index case of the Kambian cluster. After a 21-d period of quarantine, case *L* was discharged on 18 July 2015. A sample from case *L*’s semen (19560_EMLK, labeled in red, GenBank accession no. KU296821) was collected on 7 September 2015. The virus genome isolated from the deceased case *K* (020380_EMLK, GenBank accession no. KU296775) is genetically identical to case *L*, which also clusters closely with case *M* (*K*’s 23-year-old daughter, 20525_EMLK, GenBank accession no. KU296487). For each cluster case, minority variants for three key positions can be found in (b). Symptom onset of case *M* (3 September 2015) was 15 d later than onset of case *K* (26 August 2015). Case *K* is genetically identical to three known contacts of *K*: case *N* (020484_EMLK, older daughter of *K*, GenBank accession no. KU296462), case *O* (20547_EMLK, sister of *K*, GenBank accession no. KU296455), case *P* (20524_EMLK, grandchild of K, GenBank accession no. KU296424). Case *Q* (20573_EMLK, GenBank accession no. KU296654), also from the same village, is the most recent sampled case from this cluster. The lineage is related to earlier viruses from lineage F (19521_EMLK, 15543_EMLK, KT7095, and 15421_EMLK, see Fig. 2). See legend of Fig. 3(b) for additional figure details. (b) Minor variants in the Kambia lineage. In genomes from the Kambia cluster (a) three genome positions (3993, 8494, and 13518) showed changes across the entire lineage leading from 19521 through to all genomes in the family cluster. The presence of each of the two variant nucleotides was counted in the raw read set for each sample to gain additional information about possible transmission patterns. Positions with minor variants at >1% frequency are marked with a red asterisk. Positions 3994, 8496, and 13520 showed mixed nucleotides in samples from cases *K* and *M*, similar to the case *L* semen sample (but not in the case *L* initial sample). Later cases in the lineage (*N–Q*) showed predominately one of the variants at each of the three positions, although position 13520 showed some persistence of the minor variant C. These data further support the phylogenetic conclusions based on the consensus genome sequence with the *L* semen sample containing minor variants at the three positions that increase in frequency in samples from cases *K* and *M* and become the dominant nucleotide in cases *N–Q*.

There is accumulating evidence of EBOV sexual transmission (Deen et al. 2015; Christie et al. 2015; Mate et al. 2015; Blackley et al. 2016; Sow et al. 2016; Uyeki et al. 2016). On 29 August 2015 in the Kambia district, a post-mortem swab from case *K* tested positive for EBOV, some 50 d following the last confirmed case in this district. The viral genome from case *K* (020380_EMLK) clustered with a genome from case *L* from a blood sample collected on 7 July 2015 (19560R_EMLK, Fig. 5a). Case *L* was an EVD survivor, who was released from quarantine on 18 July 2015 and subsequently had sexual contact with *K* during August 2015. *L* provided a semen sample on 7 September 2015 from which an EBOV genome was obtained (19560_EMLK). The viral genome obtained from *L*’s semen was identical to the virus genome in *L*’s initial blood sample, collected 2 months earlier during acute EVD (Fig. 5a). The clustering of genomes from case *L* with those from *K*, and from several secondary contacts of *K* (cases *N*, *O*, *P*, and *Q*) indicates transmission among these cases in Kambia (Fig. 5a). In addition, the absence of nucleotide changes between the virus genomes of the two *L* samples suggests that the virus was maintained in a low replicating state within *L*. Consistent with this pattern, reduced virus evolutionary rate after virus re-emergence was also recently reported (Blackley et al. 2016). Furthermore, at three positions in the virus genome (3,993, 8,494 and 13,518), minority variants were present in the *K* and *M* read sets that show a transition between the majority nucleotide in *L* and the majority nucleotide in the viruses later in the putative transmission chain (Fig. 5b). Thus mixed nucleotide variants at three positions in *L*’s semen virus genome were consistent with *L* as the direct source of virus for *K* and *M*.

An alternate transmission route might be contact of *K* with unknown EVD cases in the community. However, such a hypothesis would require that the virus in this unidentified contact was as close, or more closely related to the viruses sequenced from the known cases, which had only three nucleotide differences between L and K. Alternately, transmission from *L* to *K* could have occurred via non-sexual contact or with other body fluids; however, given that *L*'s blood was negative but *L*'s semen was genome positive, between these two possibilities semen is the more likely source of *K*'s infection. There was no report of sexual contact between L and M, so tentatively M might have been infected from L’s bodily fluid or while taking care of K. However, the phylogenetic analysis strongly supports viral transmission between these cases (Fig. 5a), with sexual transmission from *L* to *K* as the most likely component in the transmission chain.

The local sequencing described here was rapid enough to be epidemiologically useful; however, a comprehensive genome database across the outbreak was essential to identify sources of new infections. During the course of this project, the sequence data that were generated contributed more than a third of the 1500 EBOV Makona genomes now available and represent 23.8% of the 2015 Sierra Leone cases (see Supplementary Fig. 2). These data were made available to all groups involved in outbreak sequencing (Goodfellow et al. 2015a,b; Neher and Bedford 2015) and yielded a sufficiently comprehensive set of viral genomes to identify transmission chains in other countries and across borders (Gardy et al. 2015).

In future epidemics, rapid and local sequencing of pathogens at the onset and the end of the outbreak can support outbreak investigation and control, but sequencing and data sharing during peak transmission should also be maintained to provide the genetic context for contact tracing and control of new cases. With the increasing global risk of viral zoonosis, the success of this project provides a strong incentive to establish and maintain local sequencing facilities throughout the world.

## Supplementary Material

Supplementary Data

## Author information

The 554 new EBOV genomes are deposited in GenBank (accession nos. KU296293–KU296846) and the short read data can be accessed under the study Accession no. SRP068607.
