# Supplementary material for: Rapid outbreak sequencing of Ebola virus in Sierra Leone identifies transmission chains linked to sporadic cases
Source: Virus Evol. 2016 Jun 22;2(1):vew016. doi: 10.1093/ve/vew016 (PMC5499387; doi:10.1093/ve/vew016)
Supplement: Supplementary Data [file vew016_Supp_01.zip › Supplementary_Tables.pdf]

SM Table 1 Page 1 Metadata 554

Genome_name	GenBank	Sample collection date	Specimen type	District	Genome_length	Mapped_reads	Median_read_length	Coverage
0106_C2_KT2315	KU296812	02/12/2014	blood	Port_Loko	18882	22368	210	225.08
0106_C2_KT2316	KU296774	02/12/2014	blood	Port_Loko	18879	22791	204	222.13
0106_C2_KT2317	KU296735	02/12/2014	blood	Port_Loko	18402	57068	201	561.31
0106_C2_KT2321	KU296762	02/12/2014	blood	Port_Loko	18884	46702	208	464.94
0106_C2_KT2449	KU296465	05/12/2014	blood	Western_Urban	18872	74014	185	647.11
0106_C2_KT2641	KU296709	12/12/2014	blood	not_stated	18479	22233	207	224.99
0106_C2_PL7375	KU296498	27/05/2015	blood	Port_Loko	18451	22058	194	208.02
0109_C2_020380_EMLK	KU296775	28/08/2015	swab	Kambia	18881	176039	186	1547.72
0205_C1_PL5249	KU296744	24/03/2015	blood	Kambia	18746	73893	179	626.75
0205_C1_PL5260	KU296591	23/03/2015	swab	Kambia	18768	170858	193	1574.93
0205_C1_PL5294	KU296764	24/03/2015	swab	Kambia	18889	248158	200	2364.78
0205_C1_PL5361	KU296415	26/03/2015	swab	Kambia	18885	65350	207	647.10
0205_C1_PL5428	KU296532	27/03/2015	swab	Kambia	18542	261383	170	2114.53
0205_C2_PL5674	KU296699	31/03/2015	blood	Port_Loko	18886	221357	206	2180.04
0205_C2_PL5801	KU296844	04/04/2015	swab	Kambia	18449	298675	192	2784.54
0205_C2_PL5803	KU296795	04/04/2015	blood	Kambia	18889	351931	214	3614.52
0205_C2_PL5868	KU296550	06/04/2015	swab	Kambia	18439	252021	217	2692.56
0205_C2_PL5950	KU296559	08/04/2015	blood	Port_Loko	18872	28266	212	287.57
0205_C2_PL6049	KU296743	11/04/2015	blood	Kambia	18881	66818	210	672.39
0205_C2_PL6070	KU296614	11/04/2015	swab	Kambia	18338	162077	214	1714.63
0205_C2_PL6086	KU296651	12/04/2015	blood	Kambia	18437	211192	189	1935.85
0205_C2_PL6158	KU296308	13/04/2015	swab	Kambia	18873	99401	205	974.37
0208_C1_77667_EMLK	KU296361	08/01/2015	non_stated	non_stated	18777	65915	211	670.49
0208_C1_77669_EMLK	KU296367	08/01/2015	blood	Western_Urban	18883	32173	217	335.65
0208_C1_77670_EMLK	KU296329	08/01/2015	blood	Western_Urban	18884	67597	216	701.60
0208_C1_77673_EMLK	KU296458	08/01/2015	blood	Western_Urban	18872	48434	215	500.46
0208_C1_77675_EMLK	KU296807	08/01/2015	blood	Western_Urban	18883	40215	217	419.55
0208_C1_77676_EMLK	KU296309	08/01/2015	blood	Western_Urban	18884	192707	215	1989.93
0208_C1_77677_EMLK	KU296558	08/01/2015	blood	Western_Urban	18881	74070	210	745.37
0208_C1_77679_EMLK	KU296512	08/01/2015	blood	Western_Urban	18882	44679	217	466.14
0208_C1_77681_EMLK	KU296435	08/01/2015	blood	not_stated	18749	100436	201	969.59
0208_C1_MK10128R	KU296502	31/07/2015	blood	Tonkolili	18747	250036	217	2627.48
0208_C1_MK10173	KU296313	01/08/2015	blood	Tonkolili	18881	164722	216	1709.94
0208_C2_KT4828R	KU296745	10/02/2015	swab	not_stated	18872	104731	208	1043.31
0208_C2_KT4935R	KU296467	11/02/2015	swab	Western_Rural	18868	134078	205	1314.63
0208_C2_KT5022R	KU296519	13/02/2015	swab	WesterN	18266	95732	205	969.59
0208_C2_KT5245R	KU296411	22/02/2015	blood	not_stated	18882	82287	205	806.22
0305_C1_PL4651	KU296841	12/03/2015	blood	Port_Loko	18886	83743	203	811.45
0305_C1_PL4696	KU296513	13/03/2015	blood	Port_Loko	18308	210962	214	2235.45
0305_C1_PL4844	KU296527	16/03/2015	blood	Port_Loko	18887	73763	214	757.66
0305_C1_PL4932	KU296547	18/03/2015	blood	Port_Loko	18751	193466	217	2032.57
0305_C1_PL5025	KU296412	20/03/2015	blood	Kambia	18749	104038	211	1059.86
0305_C1_PL5201	KU296612	23/03/2015	blood	Port_Loko	18885	114067	208	1135.54
0305_C1_PL5202	KU296396	23/03/2015	blood	Port_Loko	18874	124214	214	1276.75
0305_C1_PL5228	KU296535	23/03/2015	blood	Port_Loko	18873	47212	205	462.79
0305_C2_PL4570	KU296451	10/03/2015	blood	Port_Loko	18885	73612	212	748.40
0507_C1_PL4226	KU296615	02/03/2015	blood	Kambia	18791	45766	188	409.17
0507_C1_PL4248	KU296658	03/03/2015	blood	Port_Loko	18873	65562	183	566.24
0507_C1_PL4249	KU296349	03/03/2015	blood	Port_Loko	18874	27614	203	267.74
0507_C1_PL4283	KU296840	03/03/2015	blood	Port_Loko	18886	89889	189	804.37
0507_C1_PL4322	KU296677	05/03/2015	swab	Port_Loko	18876	73001	201	700.00
0507_C1_PL4443	KU296418	07/03/2015	swab	Port_Loko	18874	87265	201	836.87
0507_C1_PL4671	KU296782	12/03/2015	blood	Port_Loko	18798	100067	180	851.73
0507_C1_PL4679	KU296623	12/03/2015	blood	not_stated	18872	12920	200	123.23
0507_C1_PL4680	KU296689	12/03/2015	swab	not_stated	18750	31006	174	254.66
0507_C1_PL5179	KU296638	22/03/2015	blood	Port_Loko	18884	38093	184	330.82
0507_C2_MK0390	KU296398	22/12/2014	blood	Bombali	18773	138811	184	1212.64
0507_C2_MK0392	KU296691	22/12/2014	blood	Bombali	18872	16637	190	149.87
0507_C2_MK0664	KU296761	30/12/2014	blood	not_stated	18751	11548	209	116.40
0507_C2_MK0725	KU296629	01/01/2015	blood	Bombali	18749	20610	200	197.87
0806_C1_KT4677	KU296608	03/02/2015	swab	Western_Rural	18883	224411	210	2258.02
0806_C1_KT4695	KU296549	04/02/2015	swab	Western_Rural	18874	299420	201	2871.42
0806_C1_KT4715	KU296707	06/02/2015	blood	not_stated	18067	54997	160	426.17
0806_C1_KT4730	KU296416	07/02/2015	blood	not_stated	18875	80866	209	809.73
0806_C1_KT4801	KU296722	10/02/2015	blood	not_stated	18748	31149	191	284.11
0806_C1_KT5073	KU296553	16/02/2015	blood	not_stated	18873	189462	210	1907.36
0806_C1_MK1040	KU296528	11/01/2015	blood	Bombali	18751	18280	204	179.38
0806_C1_MK1079	KU296815	11/01/2015	swab	Bombali	18751	110593	190	1002.66
0806_C1_PL7376	KU296740	27/05/2015	blood	Port_Loko	18878	104508	210	1051.83
0806_C1_PL7401	KU296819	27/05/2015	blood	Port_Loko	18876	51737	206	509.81
0806_C1_PL7429	KU296617	29/05/2015	blood	Port_Loko	18871	54029	201	518.21
0806_C1_PL7451	KU296837	29/05/2015	blood	Port_Loko	18873	20206	203	195.93
0806_C1_PL7496	KU296846	31/05/2015	blood	Port_Loko	18758	6501	206	64.46
0806_C1_PL7511	KU296685	01/06/2015	blood	Port_Loko	18400	88043	218	947.42
0806_C1_PL7551	KU296322	02/06/2015	blood	Port_Loko	18872	77368	216	803.52
0806_C2_KT2669	KU296726	13/12/2014	blood	not_stated	18870	16880	213	172.65
0806_C2_KT2810	KU296778	16/12/2014	blood	not_stated	18749	54850	206	544.14
0806_C2_KT4054	KU296429	13/01/2015	blood	not_stated	18750	49958	211	508.90
0806_C2_KT4553	KU296698	29/01/2015	blood	not_stated	18885	156482	216	1624.07
0806_C2_KT4555	KU296687	29/01/2015	blood	not_stated	18866	155041	216	1610.73
0806_C2_KT4622	KU296587	01/02/2015	blood	not_stated	18885	26334	214	270.52
0806_C2_KT4631	KU296607	02/02/2015	blood	Western_Urban	18817	74450	210	751.74
0806_C2_KT4663	KU296570	03/02/2015	blood	not_stated	18075	29261	216	317.30
0806_C2_PL1387	KU296434	04/01/2015	blood	Port_Loko	18199	113783	216	1225.42
0806_C2_PL2101	KU296714	18/01/2015	blood	Port_Loko	18873	16597	211	167.97
0806_C2_PL2668	KU296294	29/01/2015	blood	Port_Loko	18075	96193	212	1021.80
0806_C2_PL2921	KU296506	03/02/2015	blood	Kambia	17554	184316	214	2036.98
0807_C1_14729_EMLK	KU296392	18/04/2015	non_stated	Kambia	18873	54394	161	406.38
0807_C1_14743_EMLK	KU296298	19/04/2015	non_stated	Kambia	18872	90774	193	832.13
0807_C1_14748_EMLK	KU296769	19/04/2015	non_stated	Kambia	18874	87490	179	737.04
0807_C1_14750_EMLK	KU296459	20/04/2015	non_stated	Kambia	18874	64501	189	577.55

SM Table 1 Page 2 Metadata 554

0807_C1_PL3984	KU296330	25/02/2015	blood	Port_Loko	18873	13005	201	124.72
0807_C1_PL4119	KU296645	28/02/2015	blood	Port_Loko	18746	10272	201	99.18
0807_C1_PL4121	KU296305	28/02/2015	blood	not_stated	18874	42714	198	402.83
0807_C1_PL4123	KU296752	28/02/2015	blood	Port_Loko	18749	16453	206	163.22
0807_C1_PL4125	KU296471	28/02/2015	blood	Port_Loko	18752	15352	206	152.28
0807_C1_PL4126	KU296478	28/02/2015	blood	Port_Loko	18872	134554	201	1290.50
0807_C1_PL4763	KU296503	14/03/2015	blood	Kambia	18882	9618	203	93.22
0807_C1_PL4784	KU296660	14/03/2015	blood	Port_Loko	18735	5693	207	56.82
0807_C1_PL4785	KU296688	14/03/2015	blood	Port_Loko	18868	22556	200	215.18
0807_C1_PL4804	KU296766	15/03/2015	blood	Port_Loko	18875	50606	196	471.88
0807_C1_PL4813	KU296798	14/03/2015	blood	Port_Loko	18750	38250	193	352.92
0807_C1_PL4864	KU296437	16/03/2015	blood	Kambia	18872	35697	200	340.48
0807_C1_PL4865	KU296633	16/03/2015	blood	Kambia	18747	41403	201	399.74
0807_C1_PL4866	KU296390	16/03/2015	blood	Kambia	18749	58253	186	515.76
0807_C1_PL7577	KU296385	03/06/2015	blood	Port_Loko	18888	36711	204	357.63
0807_C1_PL8429	KU296440	24/06/2015	blood	Port_Loko	18873	34102	210	343.31
0807_C1_PL8486	KU296446	25/06/2015	blood	Port_Loko	18883	27061	211.5	274.44
0807_C1_PL8630	KU296325	29/06/2015	blood	Port_Loko	18880	60816	202	586.26
0807_C2_MK2826	KU296405	08/03/2015	blood	Bombali	18678	115022	212	1182.37
0809_C1_020484_EMLK	KU296462	04-Sep-15	blood	Kambia	18882	74342	213	759.88
0809_C1_19560_EMLK	KU296821	07/09/2015	semen	Kambia	18399	136255	214	1436.68
0908_C1_KT7105	KU296319	16/07/2015	blood	Western_Urban	18887	153158	217	1597.51
1007_C1_MK1782	KU296792	06/02/2015	blood	not_stated	18872	81391	200	776.31
1007_C1_PL4028	KU296552	26/02/2015	blood	Port_Loko	18874	27744	201	266.06
1007_C1_PL4059	KU296485	26/02/2015	blood	Port_Loko	18886	60645	182	520.20
1007_C1_PL4094	KU296476	27/02/2015	blood	Kambia	18889	83689	200	797.50
1007_C1_PL4187	KU296567	01/03/2015	blood	Port_Loko	18886	42777	214	439.41
1007_C1_PL4190	KU296351	01/03/2015	blood	Port_Loko	18874	93036	179	783.77
1007_C1_PL4346	KU296641	04/03/2015	swab	Kambia	18871	148688	209	1489.16
1007_C2_MK0020	KU296551	08/12/2014	blood	Bombali	18883	171095	214	1757.80
1007_C2_MK0024	KU296705	08/12/2014	blood	Bombali	18870	6112	215	63.16
1007_C2_MK0045	KU296776	09/12/2014	blood	Bombali	18885	384558	217	4011.54
1007_C2_MK0085	KU296738	10/12/2014	blood	Bombali	18885	49849	215	514.72
1007_C2_MK0391	KU296675	22/12/2014	swab	Bombali	18873	17660	200	168.43
1007_C2_MK0607	KU296335	28/12/2014	blood	not_stated	18873	59361	201	569.30
1007_C2_MK0894	KU296356	06/01/2015	blood	Bombali	18882	33970	216	352.62
1007_C2_MK1000	KU296384	10/01/2015	blood	Bombali	18872	95311	203	924.22
1007_C2_MK1014	KU296316	10/01/2015	swab	Bombali	18883	450143	210	4529.33
1007_C2_MK1888	KU296708	09/02/2015	blood	not_stated	18749	9477	214	98.06
1007_C2_PL4105	KU296694	28/02/2015	blood	Port_Loko	18749	26030	200	249.90
1009_C1_20524_EMLK	KU296424	06/09/2015	blood	Kambia	18742	43503	204	427.09
1009_C1_20525_EMLK	KU296487	06/09/2015	blood	Kambia	18742	177510	191	1619.59
1009_C1_20547_EMLK	KU296455	07/09/2015	blood	Kambia	18253	159386	209	1650.35
1105_C1_MK2405	KU296542	25/02/2015	blood	not_stated	18873	45512	215	470.24
1105_C1_MK2427	KU296759	25/02/2015	blood	not_stated	18885	297062	215	3067.37
1105_C1_MK2431	KU296562	25/02/2015	swab	not_stated	18885	351277	216	3645.77
1105_C1_MK2436	KU296626	26/02/2015	blood	Bombali	18886	174632	210	1756.86
1105_C1_MK2673	KU296749	03/03/2015	swab	Bombali	18322	339287	220	3703.60
1105_C1_MK2790	KU296452	07/03/2015	blood	Bombali	18750	202572	188	1815.05
1105_C2_MK2334	KU296366	23/02/2015	swab	not_stated	18315	172938	183	1539.12
1105_C2_MK2369	KU296832	25/02/2015	blood	not_stated	18881	45850	212	466.24
1105_C2_MK2370	KU296760	25/02/2015	blood	not_stated	18883	121860	209	1219.70
1105_C2_MK2371	KU296397	25/02/2015	blood	not_stated	18441	295678	202	2918.14
1105_C2_MK2383R	KU296583	25/02/2015	blood	Bombali	18738	129979	201	1255.53
1209_C1_020573_EMLK	KU296654	not_stated	non_stated	not_stated	18816	140735	206	1391.20
1309_C2_MK13013	KU296454	12/09/2015	blood	Bombali	18873	194356	209	1946.34
1507_C1_13204_EMLH	KU296544	16/06/2015	blood	not_stated	18873	24606	192	224.25
1507_C1_13268_EMLH	KU296603	23/06/2015	blood	Western_Urban	18751	29128	193	268.74
1507_C1_13275_EMLH	KU296430	24/06/2015	blood	not_stated	18871	17859	206	176.03
1507_C1_18538_EMLH	KU296727	04/07/2015	blood	Western_Urban	18751	41487	201	400.47
1507_C1_18596_EMLH	KU296715	05/07/2015	blood	Western_Urban	18749	18133	201	175.05
1507_C1_18636_EMLH	KU296460	08/07/2015	blood	not_stated	18878	122717	193	1124.60
1507_C1_18647_EMLH	KU296566	09/07/2015	blood	Western_Urban	18746	12320	206	122.24
1507_C1_18648_EMLH	KU296701	09/07/2015	blood	Western_Urban	18866	71370	190	643.11
1507_C1_18649_EMLH	KU296456	10/07/2015	blood	Western_Urban	18872	56482	210	568.65
1507_C1_18650_EMLH	KU296370	10/07/2015	blood	Western_Urban	18873	114042	201	1093.71
1507_C1_18660_EMLH	KU296838	10/07/2015	blood	Western_Urban	18750	99038	200	950.76
1507_C1_18706_EMLH	KU296599	14/07/2015	blood	not_stated	18891	63657	190	572.85
1507_C2_15543_EMLK	KU296666	22/06/2015	non_stated	Western_Urban	18883	42018	194	387.18
1507_C2_15661_EMLK	KU296822	28/06/2015	non_stated	Kambia	18746	24266	201	234.30
1507_C2_15674_EMLK	KU296843	28/06/2015	non_stated	Kambia	18880	64474	201	618.11
1507_C2_15686_EMLK	KU296555	30/06/2015	non_stated	Kambia	18810	23378	190	211.29
1507_C2_19521_EMLK	KU296306	05/07/2015	non_stated	Kambia	18879	30346	209	303.80
1507_C2_MK0363	KU296545	21/12/2014	swab	not_stated	18885	69984	190	629.99
1507_C2_MK0393	KU296496	22/12/2014	swab	not_stated	18751	34708	176	288.75
1507_C2_MK0574	KU296332	28/12/2014	blood	not_stated	18870	33016	202	318.44
1507_C2_MK0575	KU296681	28/12/2014	blood	not_stated	18889	120323	188	1070.16
1507_C2_MK0584	KU296339	28/12/2014	blood	Bombali	18788	168014	208	1681.22
1507_C2_MK0709	KU296373	31/12/2014	blood	Tonkolili	18870	43658	199	414.14
1507_C2_MK0857	KU296789	05/01/2015	blood	not_stated	18882	17095	213	174.74
1507_C2_MK0864	KU296334	05/01/2015	blood	not_stated	18870	19926	209	199.58
1507_C2_MK0865	KU296500	05/01/2015	blood	not_stated	18883	117107	201	1122.51
1507_C2_MK0866	KU296497	05/01/2015	blood	not_stated	18882	31331	214	321.91
1507_C2_MK0867	KU296748	05/01/2015	blood	not_stated	18872	29151	190	262.59
1507_C2_MK0868	KU296537	05/01/2015	swab	not_stated	18874	84000	193	769.95
1507_C2_MK0869	KU296534	05/01/2015	swab	Bombali	18752	154706	175	1278.76
1507_C2_MK0879	KU296410	05/01/2015	blood	Port_Loko	18883	36799	213	376.12
1507_C2_MK0888	KU296376	06/01/2015	blood	not_stated	18885	45742	203	443.25
1507_C2_MK0889	KU296794	06/01/2015	blood	not_stated	18872	132989	125	739.93
1507_C2_MK0966	KU296507	08/01/2015	blood	Bombali	18750	21909	188	196.31
1706_C1_KT4609	KU296622	31/01/2015	swab	Western Area	18888	199822	220	2115.86
1706_C1_PL7604	KU296592	04/06/2015	blood	Port_Loko	18882	228862	215	2363.52

SM Table 1 Page 3 Metadata 554

1706_C1_PL7605	KU296486	04/06/2015	blood	Port_Loko	18869	32725	214	336.46
1706_C1_PL7626	KU296753	04/06/2015	swab	Port_Loko	18881	373169	205	3656.41
1706_C1_PL7678	KU296625	06/06/2015	swab	Port_Loko	18869	46517	214	478.26
1706_C1_PL7685	KU296556	06/06/2015	blood	Port_Loko	18881	71450	214	734.14
1706_C1_PL7709	KU296426	06/06/2015	blood	Port_Loko	18261	249017	214	2645.49
1706_C1_PL7773	KU296417	08/06/2015	blood	not_stated	18756	58911	210	596.77
1706_C1_PL7774	KU296589	08/06/2015	blood	Port_Loko	18747	38805	216	405.71
1706_C1_PL7775	KU296422	08/06/2015	blood	not_stated	18386	9260	211	96.20
1706_C1_PL7792	KU296793	09/06/2015	blood	Port_Loko	18881	112604	217	1174.88
1706_C1_PL7801	KU296327	09/06/2015	blood	Port_Loko	18398	52135	214	549.74
1706_C1_PL7820	KU296804	09/06/2015	blood	Port_Loko	18880	28798	215	297.44
1708_C1_12634_EMLH	KU296653	24/03/2015	blood	Western_Urban	18884	29216	216	303.24
1708_C1_12705_EMLH	KU296490	29/03/2015	blood	Western_Urban	18872	22695	214	233.30
1708_C1_13031_EMLH	KU296665	18/05/2015	blood	Western_Urban	18818	90669	217	949.19
1708_C1_MK0367R	KU296770	21/12/2014	blood	Bombali	18885	10825	217	112.92
1708_C1_MK0369R	KU296413	21/12/2014	blood	Bombali	18874	99774	216	1036.12
1708_C1_MK0375R	KU296606	22/12/2014	blood	Bombali	18885	154952	215	1599.98
1708_C1_MK0379R	KU296480	22/12/2014	swab	Bombali	18874	61776	214	634.97
1708_C1_MK0384R	KU296628	22/12/2014	blood	Bombali	17598	220218	218	2477.73
1708_C1_MK0388R	KU296630	22/12/2014	blood	Bombali	18872	31740	217	331.33
1708_C1_MK0425R	KU296379	23/12/2014	blood	Bombali	18893	260332	220	2755.86
1708_C1_MK0431R	KU296621	23/12/2014	blood	Bombali	18872	37860	217	395.21
1708_C1_MK0491R	KU296510	23/12/2014	blood	Bombali	18885	13232	217	138.03
1708_C1_MK0531R	KU296448	26/12/2014	swab	Bombali	18894	70883	217	739.07
1708_C1_MK0533R	KU296436	26/12/2014	blood	not_stated	18862	4300	217	44.91
1708_C1_MK0592R	KU296814	28/12/2014	blood	Bombali	18872	32599	218	342.02
1708_C1_MK0704R	KU296663	31/12/2014	blood	Bombali	18873	11864	217	123.84
1708_C1_MK0723R	KU296610	01/01/2015	blood	Bombali	18879	123796	214	1272.13
1708_C1_MK0766R	KU296777	02/01/2015	blood	not_stated	18881	11913	217	124.30
1708_C1_MK0849R	KU296323	05/01/2015	blood	not_stated	18873	178153	221	1897.34
1708_C2_MK1036R	KU296834	11/01/2015	blood	Bombali	18548	57376	214	600.12
1708_C2_MK1156R	KU296605	15/01/2015	blood	Bombali	18800	104526	212	1067.50
1708_C2_MK2008R2	KU296526	12/02/2015	swab	Western_Urban	18734	96924	191	884.70
1708_C2_MK2282R	KU296788	22/02/2015	blood	Bombali	18871	33352	216	346.40
1708_C2_MK2283R	KU296833	22/02/2015	blood	Bombali	18547	111855	216	1182.05
1708_C2_MK2307R	KU296536	23/02/2015	blood	not_stated	18746	12178	216	127.33
1708_C2_MK2506R	KU296360	27/02/2015	blood	Bombali	18871	68738	214	706.65
1708_C2_MK2656R2	KU296577	03/03/2015	swab	Koinadugu	18746	18882	214	195.41
1708_C2_MK2717R	KU296425	04/03/2015	swab	Bombali	18870	61275	215	633.21
1708_C2_MK2900R2	KU296787	10/03/2015	blood	Bombali	18745	13186	214	136.47
1804_C1_MK2570	KU296346	01/03/2015	blood	not_stated	18834	89425	212	911.63
1805_C1_MK2255	KU296648	21/02/2015	blood	not_stated	18749	69404	210	703.33
1805_C1_MK2341	KU296494	24/02/2015	blood	not_stated	18867	67496	200	643.94
1805_C1_MK2382	KU296686	25/02/2015	blood	Bombali	18883	14204	209	142.17
1805_C1_MK2395	KU296336	25/02/2015	blood	not_stated	18746	29296	206	290.68
1805_C1_MK2397	KU296444	25/02/2015	blood	not_stated	18749	83619	200	802.79
1805_C1_MK2398	KU296578	25/02/2015	blood	not_stated	18870	33167	207	328.68
1805_C1_MK2710	KU296703	05/03/2015	blood	not_stated	18725	59968	202	582.86
1805_C1_MK2724	KU296695	05/03/2015	blood	Bombali	18875	43985	211	445.09
1805_C1_MK2788	KU296573	07/03/2015	blood	Bombali	18869	33073	213	338.28
1805_C1_MK2825	KU296402	08/03/2015	blood	Bombali	18885	92824	213	948.64
1805_C1_MK3043	KU296474	14/03/2015	blood	not_stated	18875	75509	215	780.09
1805_C1_MK3462	KU296565	26/03/2015	blood	Bombali	18860	20482	212	208.51
1805_C1_MK3479	KU296483	26/03/2015	blood	Bombali	18862	17790	214	182.97
1807_C1_14859_EMLK	KU296438	30/04/2015	non_stated	Kambia	18894	12313	217	128.38
1807_C1_14888_EMLK	KU296423	03/05/2015	non_stated	Kambia	18872	32473	204	316.61
1807_C1_15000_EMLK	KU296504	13/05/2015	non_stated	Kambia	18399	40160	207	408.17
1807_C1_15274_EMLK	KU296337	04/06/2015	non_stated	Kambia	18376	35359	186	319.42
1807_C1_15314_EMLK	KU296600	06/06/2015	non_stated	Kambia	18747	60531	220	645.77
1807_C1_15323_EMLK	KU296668	07/06/2015	non_stated	Kambia	18883	19236	218	201.70
1807_C1_15327_EMLK	KU296649	07/06/2015	non_stated	Kambia	18868	24151	219	254.72
1807_C1_15338_EMLK	KU296414	08/06/2015	non_stated	Kambia	18883	117571	220	1245.26
1807_C1_15349_EMLK	KU296442	09/06/2015	non_stated	Kambia	18869	42555	217	444.29
1807_C1_15360_EMLK	KU296399	08/06/2015	non_stated	Kambia	18884	29492	218	309.23
1807_C1_15373_EMLK	KU296802	10/06/2015	non_stated	Kambia	18747	31270	216	326.93
1807_C1_15421_EMLK	KU296796	13/06/2015	non_stated	Kambia	18768	16112	211	163.97
1807_C1_15470_EMLK	KU296543	16/06/2015	non_stated	Kambia	18870	37860	201	363.15
1807_C1_77724_EMLK	KU296656	10/01/2015	blood	Western_Urban	18736	21227	179	180.14
1807_C1_MK0464	KU296835	24/12/2014	blood	not_stated	18874	46325	199	439.35
1807_C1_MK0551	KU296717	27/12/2014	blood	not_stated	18886	114834	191	1039.75
1807_C1_PL8310	KU296618	21/06/2015	blood	Port_Loko	18873	175327	220	1857.97
1807_C1_PL8780	KU296404	03/07/2015	blood	Port_Loko	18387	203017	221	2219.30
1807_C1_PL8896	KU296342	06/07/2015	blood	Port_Loko	18262	26416	220	289.30
1807_C1_PL9150R	KU296522	12/07/2015	swab	Port_Loko	18885	214514	220	2271.79
1807_C1_PL9192R	KU296401	13/07/2015	breast_milk	Port_Loko	18262	20205	218	219.07
1807_C1_PL9199R	KU296371	13/07/2015	blood	Port_Loko	18540	254575	213	2650.11
1807_C2_10354_EMLK	KU296314	08/02/2015	blood	not_stated	18764	59162	207	589.60
1807_C2_10356_EMLK	KU296441	08/02/2015	blood	not_stated	18871	32589	215	336.75
1807_C2_10394_EMLK	KU296576	09/02/2015	blood	Western_Urban	18892	23391	217	243.92
1807_C2_10398_EMLK	KU296473	10/02/2015	blood	Western_Urban	18875	65307	214	671.23
1807_C2_10402_EMLK	KU296635	10/02/2015	blood	Western_Urban	18872	44576	214	458.23
1807_C2_10415_EMLK	KU296741	10/02/2015	swab	Western_Urban	18757	41995	211	427.63
1807_C2_10454_EMLK	KU296679	12/02/2015	blood	Western_Urban	18530	47562	186	426.08
1807_C2_10455_EMLK	KU296388	12/02/2015	blood	Western_Urban	18884	59592	211	602.74
1807_C2_14521_EMLK	KU296755	13/02/2015	non_stated	Western_Urban	18885	121717	210	1224.58
1807_C2_14549_EMLK	KU296594	15/02/2015	blood	Western_Urban	18749	85334	197	805.59
1807_C2_14687_EMLK	KU296443	21/02/2015	swab	Western_Urban	18884	39378	214	404.54
1807_C2_14723_EMLK	KU296541	18/04/2015	blood	Kambia	18872	65742	214	675.82
1807_C2_14749_EMLK	KU296763	19/04/2015	blood	Kambia	18485	42318	183	373.16
1807_C2_14763_EMLK	KU296518	21/04/2015	swab	Kambia	18882	59921	218	628.34
1807_C2_14776_EMLK	KU296450	23/04/2015	swab	Kambia	18882	35453	218	371.77
1807_C2_14782_EMLK	KU296639	23/04/2015	swab	Kambia	18883	71076	217	741.51

SM Table 1 Page 4 Metadata 554

1807_C2_14795_EMLK	KU296737	25/04/2015	swab	Kambia	18878	45951	214	472.22
1807_C2_14830_EMLK	KU296345	27/04/2015	swab	Kambia	18892	113507	210	1141.55
1807_C2_14846_EMLK	KU296831	29/04/2015	blood	Kambia	18881	31174	217	325.26
2005_C2_PL6656	KU296523	01/05/2015	swab	Port_Loko	18836	25184	213	258.04
2005_C2_PL7053	KU296757	15/05/2015	blood	Port_Loko	18278	19300	209	199.57
2005_C2_PL7055	KU296484	15/05/2015	swab	Port_Loko	18388	9174	210	94.79
2104_C1_MK2342	KU296347	24/02/2015	blood	not_stated	18254	424106	210	4414.37
2104_C1_MK2343	KU296640	24/02/2015	blood	not_stated	18887	208619	201	1999.26
2104_C1_MK2344	KU296586	24/02/2015	blood	not_stated	18885	361683	203	3504.78
2104_C1_MK2362	KU296842	24/02/2015	blood	not_stated	18907	158349	201	1515.90
2104_C1_MK2364	KU296719	24/02/2015	blood	not_stated	18873	123033	211	1245.12
2104_C1_MK2365	KU296363	25/02/2015	blood	not_stated	18549	242854	203	2395.93
2104_C1_MK2367	KU296652	25/02/2015	blood	not_stated	18886	159513	211	1613.20
2104_C1_MK2437	KU296469	26/02/2015	blood	not_stated	18877	180550	208	1798.13
2104_C1_MK2449	KU296466	26/02/2015	swab	not_stated	18886	87730	215	905.82
2104_C2_MK2366	KU296810	25/02/2015	blood	not_stated	18751	89008	203	868.68
2104_C2_MK2447	KU296642	26/02/2015	swab	not_stated	18815	272561	205	2679.97
2104_C2_MK2448	KU296818	26/02/2015	blood	not_stated	18882	163302	205	1599.98
2104_C2_MK2850R	KU296374	09/03/2015	blood	Bombali	18747	48947	205	483.02
2104_C2_MK2938	KU296771	11/03/2015	blood	Bombali	18872	96797	206	954.02
2104_C2_MK2983	KU296393	12/03/2015	blood	Bombali	18861	101705	202	981.41
2104_C2_MK3050	KU296445	14/03/2015	blood	Koinadugu	18861	46305	211	468.92
2104_C2_MK4165	KU296575	16/04/2015	swab	Koinadugu	18884	199386	203	1932.20
2208_C1_MK0433R2	KU296845	23/12/2014	blood	Bombali	18746	5862	217	61.60
2208_C1_MK0434R2	KU296554	23/12/2014	blood	Bombali	18872	10750	216	111.65
2208_C1_MK0549R2	KU296825	27/12/2014	blood	Bombali	18735	5066	216	53.00
2208_C1_MK2789R3	KU296613	07/03/2015	blood	Bombali	18746	20082	217	211.04
2208_C1_MK2918R	KU296300	10/03/2015	swab	Koinadugu	18871	121526	218	1275.09
2208_C1_MK3183R2	KU296800	17/03/2015	swab	Bombali	18882	99224	216	1029.97
2208_C1_PL4127R	KU296529	28/02/2015	blood	Port_Loko	18746	51989	216	543.57
2208_C1_PL4169R	KU296828	01/03/2015	blood	Port_Loko	18870	39569	216	411.00
2208_C1_PL4197R	KU296299	02/03/2015	blood	Port_Loko	18881	53656	218	562.67
2208_C1_PL4203R	KU296311	02/03/2015	blood	Port_Loko	18749	31958	216	334.09
2208_C1_PL4256R	KU296509	03/03/2015	blood	Port_Loko	18748	159478	212	1633.23
2208_C1_PL4284R	KU296829	03/03/2015	blood	Port_Loko	18869	23480	217	245.14
2208_C1_PL4292R	KU296827	03/03/2015	swab	Kambia	18223	95503	215	1021.96
2208_C1_PL4432R	KU296481	07/03/2015	swab	Kambia	18882	23991	216	249.03
2208_C1_PL4736R	KU296724	13/03/2015	swab	Kambia	18747	30185	216	315.58
2208_C2_12615_EMLH	KU296521	22.03.2015	blood	Western_Rural	18882	40091	215	414.03
2208_C2_15207R_EMLK	KU296488	29/05/2015	non_stated	Kambia	18746	20083	214	207.84
2208_C2_15438R_EMLK	KU296353	14/06/2015	non_stated	Kambia	18881	33771	217	352.36
2208_C2_18638R_EMLH	KU296348	08/07/2015	blood	Western_Urban	18746	33986	214	351.72
2208_C2_18642R_EMLH	KU296357	08/07/2015	blood	Western_Urban	18882	76420	214	785.16
2208_C2_18659R_EMLH	KU296585	10/07/2015	blood	Western_Urban	18866	32495	216	337.59
2208_C2_18687R_EMLH	KU296650	12/07/2015	blood	Western_Urban	18872	40702	217	424.88
2208_C2_19560R_EMLK	KU296580	07/07/2015	non_stated	Kambia	18881	56393	216	585.40
2208_C2_356_EMLH	KU296546	21/01/2015	blood	not_stated	18882	8370	216	86.88
2208_C2_651_EMLH	KU296457	07/02/2015	blood	not_stated	18870	22964	217	239.74
2208_C2_75711_EMLH	KU296596	15/01/2015	blood	Western_Urban	18870	34856	216	362.04
2208_C2_75722_EMLH	KU296791	15/01/2015	blood	Western_Rural	18881	116709	217	1217.71
2208_C2_75731_EMLH	KU296564	16/01/2015	blood	Western_Urban	18882	14373	216	149.20
2208_C2_75779_EMLH	KU296407	19/01/2015	blood	Western_Urban	18883	20804	216	215.94
2208_C2_762_EMLH	KU296728	12/02/2015	blood	not_stated	18871	14691	216	152.59
2208_C2_PL7483R	KU296673	31/05/2015	blood	not_stated	18747	50373	216	526.65
2208_C2_PL7834R2	KU296326	10/06/2015	blood	Port_Loko	18766	22061	214	228.06
2507_C1_10012_EMLK	KU296634	18/01/2015	blood	Western_Rural	18803	38542	210	389.46
2507_C1_10022_EMLK	KU296482	19/01/2015	blood	Western_Urban	18762	25005	213	257.22
2507_C1_10023_EMLK	KU296333	19/01/2015	blood	Western_Urban	18868	293808	203	2849.63
2507_C1_10025_EMLK	KU296470	19/01/2015	blood	Western_Urban	18797	57837	214	596.92
2507_C1_77725_EMLK	KU296315	10/01/2015	blood	not_stated	18812	29183	216	304.05
2507_C1_77727_EMLK	KU296303	10/01/2015	blood	Western_Urban	18870	63038	201	604.66
2507_C1_77737_EMLK	KU296664	11/01/2015	blood	Western_Urban	18886	51273	215	529.40
2507_C1_77742_EMLK	KU296378	11/01/2015	blood	Western_Urban	18815	68107	213	698.63
2507_C1_77746_EMLK	KU296742	11/01/2015	blood	not_stated	18883	100404	200	957.09
2507_C1_77747_EMLK	KU296756	11/01/2015	blood	not_stated	18884	54568	214	560.59
2507_C1_77748_EMLK	KU296358	11/01/2015	blood	not_stated	18869	100265	201	961.78
2507_C1_77749_EMLK	KU296359	11/01/2015	blood	Western_Urban	18884	30642	217	319.66
2507_C1_77750_EMLK	KU296530	12/01/2015	blood	Western_Urban	18885	302169	214	3104.10
2507_C1_77751_EMLK	KU296387	12/01/2015	blood	Western_Urban	18884	258922	204	2522.87
2507_C1_77753_EMLK	KU296582	12/01/2015	blood	Western_Urban	18885	82821	209	828.87
2507_C1_77756_EMLK	KU296839	12/01/2015	blood	Western_Urban	18790	106336	187	945.08
2507_C1_77767_EMLK	KU296386	14/01/2015	blood	not_stated	18871	25035	214	257.37
2507_C1_77768_EMLK	KU296772	14/01/2015	blood	not_stated	18873	17984	217	187.72
2507_C1_77795_EMLK	KU296730	16/01/2015	swab	Western_Urban	18873	36533	213	373.59
2507_C1_MK8878	KU296684	23/07/2015	non_stated	Tonkolili	18748	108287	203	1057.00
2507_C2_10027_EMLK	KU296786	19/01/2015	blood	Western_Urban	18870	28512	217	297.66
2507_C2_10038_EMLK	KU296655	20/01/2015	blood	Western_Urban	18870	130868	208	1303.83
2507_C2_10040_EMLK	KU296678	20/01/2015	blood	Western_Urban	18757	61455	204	602.86
2507_C2_10049_EMLK	KU296341	21/01/2015	blood	not_stated	18869	25726	212	261.77
2507_C2_10065_EMLK	KU296372	22/01/2015	blood	Western_Urban	18755	15944	216	166.62
2507_C2_10066_EMLK	KU296637	22/01/2015	blood	Western_Urban	18886	79335	217	827.54
2507_C2_10080_EMLK	KU296781	22/01/2015	blood	not_stated	18884	181966	217	1898.29
2507_C2_10082_EMLK	KU296584	22/01/2015	blood	not_stated	18908	35200	214	361.16
2507_C2_10084_EMLK	KU296731	22/01/2015	swab	not_stated	18815	15606	222	167.55
2507_C2_10112_EMLK	KU296670	24/01/2015	blood	Western_Rural	18878	31416	216	326.18
2507_C2_10113_EMLK	KU296611	24/01/2015	blood	Western_Rural	18884	40614	217	423.69
2507_C2_10123_EMLK	KU296609	25/01/2015	blood	Western_Urban	18754	29797	205	293.93
2507_C2_10132_EMLK	KU296811	25/01/2015	blood	Western_Urban	18883	28647	217	298.86
2507_C2_10134_EMLK	KU296409	25/01/2015	blood	Western_Urban	18747	59223	210	600.23
2507_C2_10142_EMLK	KU296317	26/01/2015	blood	Western_Urban	18882	11544	217	120.44
2507_C2_10212_EMLK	KU296785	31/01/2015	blood	Western_Urban	18871	136881	217	1428.94
2507_C2_10244_EMLK	KU296568	02/02/2015	blood	not_stated	18882	128996	217	1345.84

SM Table 1 Page 5 Metadata 554

2507_C2_10247_EMLK	KU296674	02/02/2015	blood	not_stated	18884	15489	217	161.58
2507_C2_10248_EMLK	KU296669	02/02/2015	blood	Port_Loko	18881	67000	217	699.06
2507_C2_10278_EMLK	KU296499	03/02/2015	blood	Western_Urban	18882	15288	217	159.50
2507_C2_10352_EMLK	KU296631	08/02/2015	blood	not_stated	18882	30592	218	320.79
2507_C2_10353_EMLK	KU296751	08/02/2015	blood	Western_Urban	18883	14865	217	155.08
2507_C2_77722_EMLK	KU296479	10/01/2015	blood	Western_Urban	18871	59753	207	592.12
2507_C2_77723_EMLK	KU296563	10/01/2015	blood	Western_Urban	18872	90391	217	943.57
2605_C1_MK0885	KU296758	06/01/2015	blood	not_stated	18816	107355	178	901.47
2605_C1_MK1197	KU296301	17/01/2015	blood	not_stated	18780	212857	151	1484.78
2605_C1_MK1202	KU296581	17/01/2015	blood	Bombali	18078	108772	149	776.17
2605_C1_MK1320	KU296354	20/01/2015	blood	Bombali	18759	45053	123	247.37
2605_C2_KT5226	KU296297	21/02/2015	blood	not_stated	18875	93691	203	908.37
2605_C2_KT5233	KU296750	21/02/2015	blood	not_stated	18875	75557	190	680.52
2605_C2_KT5243	KU296736	22/02/2015	blood	not_stated	18871	80491	186	708.04
2605_C2_PL5133R	KU296489	21/03/2015	swab	Port_Loko	18400	111631	212	1164.85
2605_C2_PL5275	KU296439	24/03/2015	blood	Port_Loko	18881	131938	202	1271.79
2605_C2_PL5534	KU296784	29/03/2015	blood	Port_Loko	18387	84153	193	791.78
2605_C2_PL7136b	KU296732	18/05/2015	blood	Port_Loko	18881	71481	213	730.67
2607_C1_12004_EMLH	KU296302	14/02/2015	swab	Western_Rural	18748	9220	217	96.88
2607_C1_12106_EMLH	KU296780	18/02/2015	blood	Western_Urban	18887	208068	219	2192.27
2607_C1_12112_EMLH	KU296647	18/02/2015	blood	Western_Urban	18870	20313	217	212.06
2607_C1_12161_EMLH	KU296697	21/02/2015	blood	not_stated	18873	289104	218	3033.05
2607_C1_12178_EMLH	KU296525	22/02/2015	blood	Western_Urban	18882	93078	216	966.17
2607_C1_12179_EMLH	KU296531	22.02.2015	blood	Western_Urban	18884	74156	217	773.60
2607_C1_592_EMLH	KU296447	05/02/2015	blood	not_stated	18884	20768	217	216.65
2607_C1_611_EMLH	KU296619	05/02/2015	blood	not_stated	18871	29361	219	309.62
2607_C1_614_EMLH	KU296597	05/02/2015	blood	Western_Rural	18883	123243	217	1285.75
2607_C1_632_EMLH	KU296693	06/02/2015	blood	Western_Urban	18883	37959	217	396.01
2607_C1_634_EMLH	KU296799	06/02/2015	blood	Western_Urban	18883	16809	218	176.25
2607_C1_704_EMLH	KU296721	09/02/2015	blood	not_stated	18872	26237	219	276.66
2607_C1_705_EMLH	KU296683	09/02/2015	blood	not_stated	18748	54475	220	581.13
2607_C1_731_EMLH	KU296765	11/02/2015	blood	Western_Urban	18871	30577	217	319.20
2607_C1_732_EMLH	KU296391	11/02/2015	blood	not_stated	18887	39510	217	412.11
2607_C1_735_EMLH	KU296661	11/02/2015	blood	not_stated	18885	48900	217	510.10
2607_C1_741_EMLH	KU296295	11/02/2015	swab	Western_Urban	18883	57113	218	598.86
2607_C1_793_EMLH	KU296492	13/02/2015	blood	not_stated	18882	93084	219	981.03
2607_C1_PL4022R	KU296362	26/02/2015	blood	Port_Loko	18748	108194	210	1096.48
2607_C1_PL4052R	KU296676	26/02/2015	swab	Port_Loko	18868	131596	211	1332.15
2607_C1_PL4330R	KU296624	05/03/2015	blood	Port_Loko	18873	89226	201	855.71
2607_C2_12180_EMLH	KU296310	22/02/2015	blood	Western_Urban	18885	162215	217	1692.15
2607_C2_12181_EMLH	KU296783	23/02/2015	blood	Western_Urban	18798	20791	201	200.19
2607_C2_12189_EMLH	KU296369	23/02/2015	blood	Western_Urban	18890	82099	217	856.20
2607_C2_12192_EMLH	KU296511	23/02/2015	blood	Western_Urban	18871	117290	218	1230.64
2607_C2_12193_EMLH	KU296616	23/02/2015	blood	Western_Urban	18889	38197	218	400.39
2607_C2_12229_EMLH	KU296643	27/02/2015	blood	Western_Rural	18888	13813	217	144.07
2607_C2_12242_EMLH	KU296604	28/02/2015	blood	Western_Urban	18872	147417	220	1562.28
2607_C2_12257_EMLH	KU296702	01/03/2015	swab	Port_Loko	18884	77737	220	823.31
2607_C2_12275_EMLH	KU296538	02/03/2015	blood	Western_Urban	18884	56159	216	582.88
2607_C2_12600_EMLH	KU296449	21/03/2015	blood	Western_Urban	18871	31732	217	331.26
2607_C2_12628_EMLH	KU296692	23/03/2015	blood	Western_Rural	18870	25005	216	259.72
2607_C2_12666_EMLH	KU296803	26/03/2015	blood	Western_Rural	18885	50463	214	518.39
2607_C2_12757_EMLH	KU296468	03/04/2015	blood	Western_Urban	18873	41773	208	416.12
2607_C2_12767_EMLH	KU296768	04/04/2015	blood	Western_Urban	18884	12957	210.5	130.71
2607_C2_12781_EMLH	KU296520	06/04/2015	blood	Western_Urban	18871	104537	217	1091.29
2607_C2_12851_EMLH	KU296671	15/04/2015	blood	Western_Urban	18750	30853	217	324.16
2607_C2_12854_EMLH	KU296729	15/04/2015	blood	Western_Urban	18881	50465	217	526.54
2607_C2_12855_EMLH	KU296723	15/04/2015	blood	Western_Urban	18882	33303	217	347.46
2607_C2_12872_EMLH	KU296560	18/04/2015	blood	Western_Urban	18870	34205	216	355.28
2607_C2_12916_EMLH	KU296318	29/04/2015	blood	Western_Urban	18745	7756	217	81.51
2607_C2_12934_EMLH	KU296790	04/05/2015	blood	Western_Urban	18869	48067	217	501.84
2607_C2_12936_EMLH	KU296561	04/05/2015	blood	Western_Urban	18883	179587	214	1845.04
2807_C1_361_EMLH	KU296420	21/01/2015	blood	Western_Urban	18883	57861	217	603.64
2807_C1_362_EMLH	KU296505	21/01/2015	blood	Western_Urban	18884	182599	218	1914.56
2807_C1_375_EMLH	KU296813	21/01/2015	blood	not_stated	18871	35791	218	375.53
2807_C1_380_EMLH	KU296377	22/01/2015	blood	not_stated	18878	42547	217	444.00
2807_C1_389_EMLH	KU296593	23/01/2015	blood	Western_Rural	18884	49922	214	512.86
2807_C1_402_EMLH	KU296495	23/01/2015	swab	Western_Rural	18884	43549	216	452.00
2807_C1_405_EMLH	KU296805	24/01/2015	blood	not_stated	18800	30573	217	320.37
2807_C1_408_EMLH	KU296408	24/01/2015	blood	not_stated	18883	271248	217	2829.83
2807_C1_413_EMLH	KU296472	24/01/2015	blood	not_stated	18884	278943	218	2924.73
2807_C1_466_EMLH	KU296433	28/01/2015	blood	Western_Urban	18872	92556	211	936.75
2807_C1_468_EMLH	KU296773	29/01/2015	blood	not_stated	18874	121030	211	1224.79
2807_C1_470_EMLH	KU296662	29/01/2015	blood	not_stated	18814	86023	201	827.58
2807_C1_473_EMLH	KU296524	29/01/2015	blood	not_stated	18746	41410	205	408.67
2807_C1_480_EMLH	KU296491	29/01/2015	blood	not_stated	18871	241492	216	2508.20
2807_C1_75674_EMLH	KU296350	13/01/2015	blood	Western_Urban	18872	11522	217	120.28
2807_C1_75685_EMLH	KU296365	14/01/2015	blood	not_stated	18750	25734	217	270.38
2807_C1_75688_EMLH	KU296672	14/01/2015	blood	Western_Rural	18861	4316	215	44.62
2807_C1_75705_EMLH	KU296602	15/01/2015	blood	Western_Urban	18884	53534	217	558.47
2807_C1_75707_EMLH	KU296344	15/01/2015	blood	Western_Rural	18872	25576	215	264.27
2807_C1_75727_EMLH	KU296716	16/01/2015	blood	Western_Urban	18884	56356	218	590.90
2807_C1_75728_EMLH	KU296331	16/01/2015	blood	Western_Urban	18873	65706	217	685.85
2807_C1_75750_EMLH	KU296464	17/01/2015	blood	Western_Urban	18884	217384	217	2267.77
2807_C1_75789_EMLH	KU296382	20/01/2015	blood	not_stated	18874	27099	217	282.85
2807_C2_489_EMLH	KU296324	30/01/2015	blood	Western_Rural	18856	71758	217	749.70
2807_C2_490_EMLH	KU296746	30/01/2015	swab	not_stated	18882	36685	217	382.74
2807_C2_491_EMLH	KU296588	30/01/2015	blood	not_stated	18870	50820	217	530.55
2807_C2_505_EMLH	KU296659	31/01/2015	blood	Western_Urban	18882	51761	217	540.03
2807_C2_508_EMLH	KU296304	31/01/2015	blood	Western_Urban	18870	23252	217	242.75
2807_C2_511_EMLH	KU296646	01/02/2015	blood	Western_Urban	18870	36150	216	375.48
2807_C2_518_EMLH	KU296747	02/02/2015	blood	not_stated	18882	38915	217	406.01
2807_C2_539_EMLH	KU296823	02/02/2015	blood	Western_Urban	18881	72907	216	756.83
2807_C2_554_EMLH	KU296817	03/02/2015	swab	not_stated	18882	23787	216	246.91
2807_C2_567_EMLH	KU296427	03/02/2015	blood	not_stated	18748	35353	210	358.28
2807_C2_77685_EMLK	KU296364	08/01/2015	swab	Western_Urban	18874	60597	202	584.33

SM Table 1 Page 6 Metadata 554

2807_C2_77687_EMLK	KU296296	09/01/2015	blood	Western_Urban	18518	132178	174	1099.22
2807_C2_77688_EMLK	KU296328	09/01/2015	blood	Western_Urban	18872	88806	206	875.26
2807_C2_77693_EMLK	KU296381	09/01/2015	blood	Western_Urban	18871	128522	217	1341.68
2807_C2_77696_EMLK	KU296767	09/01/2015	blood	Western_Urban	18883	24221	217	252.69
2807_C2_77706_EMLK	KU296572	10/01/2015	blood	Western_Urban	18884	93367	217	974.01
2807_C2_77710_EMLK	KU296696	10/01/2015	blood	Western_Urban	18869	36985	217	386.14
2807_C2_77711_EMLK	KU296644	10/01/2015	blood	Western_Urban	18869	23799	214	244.69
2807_C2_77712_EMLK	KU296400	10/01/2015	blood	Western_Urban	18869	36477	214	375.04
2807_C2_77716_EMLK	KU296569	10/01/2015	blood	Western_Urban	18747	60471	211	616.10
2807_C2_MK1355R	KU296515	22/01/2015	blood	Bombali	18800	105068	125	586.82
2906_C1_PL4194	KU296501	02/03/2015	swab	Port_Loko	18871	59189	193	542.62
2906_C1_PL4347	KU296720	04/03/2015	swab	Kambia	18871	101626	201	974.74
2906_C1_PL4483	KU296389	08/03/2015	swab	Kambia	18748	41413	184	362.27
2906_C1_PL4561	KU296725	10/03/2015	swab	Port_Loko	18874	57546	189	515.28
2906_C1_PL4569	KU296598	10/03/2015	blood	Port_Loko	18748	38469	191	350.88
2906_C1_PL4872	KU296419	16/03/2015	swab	Kambia	18887	77941	201	746.93
2906_C1_PL4886	KU296461	16/03/2015	blood	Port_Loko	18885	138401	205	1355.80
2906_C1_PL5001	KU296514	19/03/2015	swab	Port_Loko	18881	124671	201	1195.14
2906_C1_PL5019	KU296627	19/03/2015	blood	Port_Loko	18870	42397	205	415.66
2906_C1_PL5099	KU296682	19/03/2015	swab	Port_Loko	18871	24856	214	255.53
2906_C1_PL5157	KU296421	22/03/2015	blood	Port_Loko	18870	107835	201	1034.35
2906_C1_PL7689	KU296712	06/06/2015	blood	Port_Loko	18871	29259	211	296.14
2906_C1_PL7946	KU296667	12/06/2015	swab	Port_Loko	18813	16623	212	169.65
2906_C1_PL7947	KU296571	12/06/2015	swab	Port_Loko	18748	43906	186	388.76
2906_C1_PL8128	KU296340	16/06/2015	swab	Port_Loko	18872	63080	183	544.83
2906_C1_PL8172	KU296428	18/06/2015	blood	Port_Loko	18884	165065	202	1590.87
2906_C2_KT5034	KU296632	14/02/2015	blood	Western_Urban	18872	31569	201	302.78
2906_C2_KT5786	KU296590	07/03/2015	blood	Western_Urban	18747	31299	209	315.55
2906_C2_MK0364	KU296453	21/12/2014	blood	Bombali	18748	19556	204	191.93
2906_C2_MK0365	KU296406	21/12/2014	blood	Bombali	18776	39645	190	358.95
2906_C2_MK0366	KU296320	21/12/2014	blood	Bombali	18884	30778	215	317.82
2906_C2_MK0368	KU296718	21/12/2014	blood	Bombali	18769	50024	200	479.75
3004_C1_KT5734	KU296595	06/03/2015	blood	Western_Rural	18864	119066	210	1199.25
3004_C1_KT5738	KU296394	06/03/2015	blood	not_stated	18874	154522	209	1547.36
3004_C1_KT5787	KU296711	07/03/2015	blood	Western_Urban	17573	183838	215	2039.97
3004_C1_KT5788	KU296704	07/03/2015	blood	Western_Urban	18248	144856	214	1540.01
3004_C1_KT5789	KU296713	07/03/2015	blood	Western_Urban	18886	43128	212	438.45
3004_C1_KT5832	KU296493	10/03/2015	blood	not_stated	18871	50781	214	522.05
3004_C1_KT5858	KU296754	10/03/2015	blood	Western_Rural	18883	60541	212	615.57
3004_C1_KT5859	KU296463	10/03/2015	blood	not_stated	18505	135097	181	1175.39
3004_C1_KT5974	KU296475	16/03/2015	blood	not_stated	18871	143285	213	1465.43
3004_C2_KT5214	KU296338	20/02/2015	blood	not_stated	18881	101915	215	1052.56
3004_C2_KT5224	KU296383	21/02/2015	blood	not_stated	18871	99882	203	968.60
3004_C2_KT5225	KU296517	21/02/2015	blood	not_stated	18734	137542	204	1350.90
3004_C2_KT5249	KU296601	22/02/2015	blood	Western_Urban	18875	150447	201	1442.69
3004_C2_KT5317	KU296352	24/02/2015	blood	Western_Rural	18836	137888	210	1390.88
3004_C2_KT5320	KU296809	24/02/2015	not_stated	Western_Rural	18871	84061	206	828.54
3004_C2_KT5382	KU296657	25/02/2015	swab	Western_Rural	18828	63080	209	633.21
3004_C2_KT5388	KU296355	25/02/2015	swab	Western_Rural	18870	110727	204	1079.70
3004_C2_KT5486	KU296680	27/02/2015	blood	not_stated	18876	122763	212	1248.70
3004_C2_KT5542	KU296557	20/02/2015	blood	not_stated	18869	201668	209	2019.99
3007_C1_12578_EMLH	KU296307	20/03/2015	blood	Western_Rural	18747	107091	219	1136.78
3007_C1_12579_EMLH	KU296548	20/03/2015	blood	Western_Rural	18871	63515	217	663.05
3007_C1_12580_EMLH	KU296574	20/03/2015	blood	Western_Rural	18872	27499	216	285.60
3007_C1_13247_EMLH	KU296830	19/06/2015	blood	not_stated	18869	96676	215	999.09
3007_C1_442_EMLH	KU296375	26/01/2015	blood	Western_Urban	18392	24290	217	260.18
3007_C1_443_EMLH	KU296539	26/01/2015	blood	Western_Urban	18748	56502	219	599.74
3007_C1_448_EMLH	KU296797	27/01/2015	blood	Western_Urban	18750	28614	218	302.16
3007_C1_75708_EMLH	KU296739	15/01/2015	blood	Western_Rural	18882	15838	217	165.24
3007_C1_77524_EMLK	KU296368	30/12/2014	blood	Western_Urban	17830	15261	216	167.76
3007_C1_77525_EMLK	KU296820	30/12/2014	blood	Western_Urban	18871	238750	201	2289.95
3007_C1_77526_EMLK	KU296824	30/12/2014	blood	Western_Urban	18869	94722	186	833.31
3007_C1_77529_EMLK	KU296477	30/12/2014	blood	Western_Urban	18871	106284	196	991.25
3007_C1_77549_EMLK	KU296293	01/01/2015	blood	Western_Urban	18763	6475	217	67.98
3007_C1_77551_EMLK	KU296808	01/01/2015	blood	Western_Urban	18406	22084	217	236.37
3007_C1_77558_EMLK	KU296733	02/01/2015	swab	Western_Urban	18868	33057	214	339.89
3007_C1_77563_EMLK	KU296432	02/01/2015	blood	Western_Urban	18884	56692	214	582.41
3007_C1_77572_EMLK	KU296508	03/01/2015	blood	Western_Urban	18832	18936	214	195.07
3007_C1_KT7095	KU296690	05/07/2015	blood	Kambia	18878	28632	218	300.31
3007_C1_MK9396	KU296321	18/07/2015	non_stated	not_stated	18869	112577	209	1127.62
3007_C2_77573_EMLK	KU296516	03/01/2015	non_stated	Western_Urban	18869	131430	216	1365.22
3007_C2_77574_EMLK	KU296700	03/01/2015	non_stated	Western_Rural	18831	22440	215	232.37
3007_C2_77577_EMLK	KU296533	03/01/2015	non_stated	Western_Urban	18739	6133	216	64.15
3007_C2_77578_EMLK	KU296806	03/01/2015	non_stated	Western_Urban	18819	14234	212	145.22
3007_C2_77580_EMLK	KU296431	03/01/2015	non_stated	Western_Urban	18746	11853	209	119.50
3007_C2_77581_EMLK	KU296540	03/01/2015	non_stated	Western_Urban	18754	22727	206	225.40
3007_C2_77582_EMLK	KU296816	03/01/2015	non_stated	Western_Urban	18746	177432	203	1732.11
3007_C2_77585_EMLK	KU296734	04/01/2015	non_stated	Western_Urban	18869	12676	211	128.31
3007_C2_77588_EMLK	KU296801	04/01/2015	non_stated	Western_Urban	18883	18966	216	196.86
3007_C2_77589_EMLK	KU296403	04/01/2015	non_stated	Western_Urban	18822	55913	204	546.60
3007_C2_77591_EMLK	KU296836	04/01/2015	non_stated	Western_Urban	18874	41484	210	417.61
3007_C2_77594_EMLK	KU296343	04/01/2015	non_stated	Western_Urban	18759	134567	190	1219.49
3007_C2_77601_EMLK	KU296826	05/01/2015	non_stated	Western_Urban	18882	30639	211	309.93
3007_C2_77612_EMLK	KU296636	05/01/2015	non_stated	Western_Urban	18748	66965	201	646.51
3007_C2_77613_EMLK	KU296579	05/01/2015	non_stated	Western_Urban	18748	29948	217	314.69
3007_C2_77615_EMLK	KU296706	05/01/2015	non_stated	Western_Urban	18746	39254	217	412.52
3007_C2_77630_EMLK	KU296779	06/01/2015	non_stated	Western_Urban	18746	13317	216	139.24
3007_C2_77632_EMLK	KU296710	06/01/2015	non_stated	Western_Urban	18883	14991	215	154.81
3007_C2_77643_EMLK	KU296395	07/01/2015	non_stated	Western_Urban	18768	24924	188	223.11
3007_C2_77644_EMLK	KU296312	07/01/2015	non_stated	Western_Urban	18744	36027	190	326.75
3007_C2_77648_EMLK	KU296620	07/01/2015	non_stated	Western_Urban	18746	26842	207	267.76
3007_C2_77649_EMLK	KU296380	07/01/2015	non_stated	Western_Urban	18748	50316	192	461.62

SM Table 2 Page 1 Primers, positions

AMPLICON_ID	FWD_PRIMER	REV_PRIMER	POOL_NAME	Position1	Position2
KM034562v1_1.1.22751	ACACAAAAAGAAAGAAGAATTTTTAGGATCTTT	CACCCTGCAGAGTTTAAGGC	Pool1	38	256
KM034562v1_1.1.26078	ACCAAGATAGAGAACAACCTAGGTCT	TTCTAATGTTTTACCCCAGGCTTTAGG	Pool2	178	400
KM034562v1_1.1.136463	GCAGGGTGATCCAACAACCT	ACTCGGCGTCATCCAGAC	Pool1	288	490
KM034562v1_1.1.118581	GACAAATTGCTCGGAATCACAAAATTC	ATGATGAAGACAAAGCATGAGAAGGAA	Pool2	464	685
KM034562v1_1.1.96185	CGCCGAGTCTCACTGAATCT	TGACTGCGCCACTTTCCA	Pool1	520	740
KM034562v1_1.1.81337	CATGCGTACCAAGGAGATTACAAAC	ACCTTCTCAAGGCAAGCCTTT	Pool2	734	963
KM034562v1_1.2.80110	ATGCCGGTCAGTTCCTCTC	TGGCATCATGTCCGGCAA	Pool1	924	1133
KM034562v1_1.2.266471	GGCAAATTCAAGTACATGCAGAGC	ATGGAGACGAACTCCTCGTTCT	Pool2	1013	1242
KM034562v1_1.2.334435	TGATGCCAACGATGCTGTGA	ACAGTTGAGGGAAAAGACCATGC	Pool1	1163	1395
KM034562v1_1.2.284911	GTCTCCATCCTCTTGCAAGGAC	GAGTTGCTTCTCAGCCTCAGT	Pool2	1278	1510
KM034562v1_1.2.306584	TCAACTGTCGGCAATTGCAC	GGCCAGGCGCTCTTTTCT	Pool1	1430	1660
KM034562v1_1.2.94906	CAACTCCAACAATATGCGGAGTCT	TCATCTTGATGGCCAGGATTGTC	Pool2	1549	1777
KM034562v1_1.2.148817	CCTGGCCAAGCTGACAGA	ACTCATGCCGTTTTCCGAGT	Pool1	1689	1883
KM034562v1_1.2.96357	CAAGATGATGATCCGACTGACTCAC	ATCTGTTAGGCACTGGCTTGG	Pool2	1817	1952
KM034562v1_1.3.203962	GCATGAGTGCACCAGATGAC	CATGCGAGGGCTGGTTGA	Pool1	1915	2134
KM034562v1_1.3.253845	TAACAGATCGACCAAGGGTGGA	ATCTGACTCTAAGGGCGGAAGG	Pool2	1987	2211
KM034562v1_1.3.271473	TCGCATGCTGACCCCAAT	CTGGCTGGGTGTTGTCACT	Pool1	2163	2383
KM034562v1_1.3.61085	AGTCAGATGATGAAGAACAGGACAG	ATGATAATACAAAACGGCATCAAATGGC	Pool2	2250	2448
KM034562v1_1.3.115064	CCAGCCAGAACATTCTTTTGAGG	GGCCAATAAAATTGTTGACCATCCA	Pool1	2417	2612
KM034562v1_1.3.116103	CCAGTGATGGTAAAGAGTACACGTATC	ACATCAAAAATTCTTCCTGTTTGCGT	Pool2	2534	2749
KM034562v1_1.3.75358	ATTGGCCAGTAATGAATCACAGGAA	CTGTGGCTTAACGCTTATTTGCAT	Pool1	2655	2876
KM034562v1_1.4.252452	TAGCCGTTTTTCAAAGTTCAATTTGAGT	ACATTGAAGACAATAAGCTTCTAGTGCT	Pool2	2872	3077
KM034562v1_1.4.51313	AGCCACAGTTATAGCCATAATGGTAAC	GTATGGCCCCTGCCCTTT	Pool1	2919	3142
KM034562v1_1.4.283502	TGGCCTAACAAGATGACAACTAGAAC	ACCTCCTCAAAACTATGATTGCAAATTG	Pool2	3142	3357
KM034562v1_1.4.177274	GATGCGCAACAGCAAACCC	CACAAACCCTGTTCAATGAGGAG	Pool1	3348	3511
KM034562v1_1.4.110197	CATTGGCTACTGTTGTGCAACAA	CCCCGAATCGCACTTTCTTCA	Pool2	3425	3655
KM034562v1_1.4.227114	GGTTTGTGCTGAGATGGTTGC	TGTCAGGTTTCCCAAAATTTTCCTCA	Pool1	3547	3757
KM034562v1_1.4.78271	ATTGAATCTAGAGATGAGACTGTCCCT	GGCCTGGAACTCAGCATGAAT	Pool2	3707	3902
KM034562v1_1.5.42730	ACCTGACATTTCGGCAAAGGA	GGGAATGTCACCTCGAGAGC	Pool1	3796	4020
KM034562v1_1.5.170899	CTGAAGGAGACTCCCCTCAATG	TTGAAGCCTCTGCTATTAGTTGCC	Pool2	3952	4176
KM034562v1_1.5.35906	ACATTCCCCGAGCTTGCC	AGCGGGATATGAACAATTTTATCTTGGT	Pool1	4050	4277
KM034562v1_1.5.223349	GCTTCAACTGCTGAACTATAGGGT	ATTTTGCTCAACGTTTACAAGATGAAGG	Pool2	4217	4440
KM034562v1_1.5.74938	ATCCCGCTAGCAGCTTTAAAGATAAA	TTGCCACCCCTAGCAATTGT	Pool1	4323	4550
KM034562v1_1.5.72370	ATATGAGGCGGGTTATATTGCCTAC	GGGCCCGATATGACATTCACC	Pool2	4501	4711
KM034562v1_1.5.245783	GGTGGCAACAGCAATACAGG	GATGGCGGCCGTAGTTGA	Pool1	4582	4805
KM034562v1_1.5.239118	GGGCCCCAAAGTGCTAATGAA	GGAGGAAAGCCTGGTTTCCAA	Pool2	4747	4932
KM034562v1_1.6.132275	CCGCCATCATGCTTGCTT	CGCAACGCTCCATTTGATCC	Pool1	4833	5069
KM034562v1_1.6.312041	TTTCCTCCAGGAGTTCGTTCTTC	TAAAGTCCTGGAGTGAAGTCATTATTGC	Pool2	4968	5192
KM034562v1_1.6.208802	GCGTTGCGTCCAGGAATTT	ACTTTGGCAAAAGAACAGGGATGA	Pool1	5100	5328
KM034562v1_1.6.221389	GGACTTTAAGATCGTTCCAATTGATCCA	CTTCTCAACCACAGCTGGAAGA	Pool2	5240	5434
KM034562v1_1.6.202261	GCCAAAGTACATTGGGTTGGAC	AAGAGACAATGGTGTAAAGCACCTT	Pool1	5366	5570
KM034562v1_1.6.270229	TCCAGTTTTACAGAATCTTCTCAGGGA	AGAGTATGTGCAATGAGAAAGATTGACA	Pool2	5505	5721
KM034562v1_1.6.297059	TGTCTCTTTTCTCTCCTAAATGTAGAGCTT	GGCTGTCCTCAGATGTAAGCA	Pool1	5617	5775
KM034562v1_1.6.253953	TACTCTTTGTCCTTATCCTCAAATTGCC	AGGACCTGATGACCCCTACTC	Pool2	5771	5959
KM034562v1_1.7.211634	GGACAGCCAGTGTGACTTGG	CCATTGTGTTGTCGCCTCAC	Pool1	5808	6022
KM034562v1_1.7.100934	CAGGTCCTTTTCAATTGGATAACCAAAA	TCCAAGCGGGATGGAAAATGT	Pool2	6000	6125
KM034562v1_1.7.95000	CACAATGGGTGTTACAGGAATATTGC	CGGACCTGAAGCCCCATC	Pool1	6060	6291
KM034562v1_1.7.136853	CGCTTGGAGTTATCCACAATAGTACATT	GCTGCTGGTAGACACTCACTC	Pool2	6166	6391
KM034562v1_1.7.251893	GTCCGGTGTCCCACCAAA	CGACACCTTCAGCGAAAGTC	Pool1	6322	6559
KM034562v1_1.7.297656	TGCCGGTATGTGCACAAAGTAT	CAAAACCGGTAGCCTGATATCTAATTGT	Pool2	6462	6686
KM034562v1_1.7.56640	AGGTGTCGTTGCATTTCTGATACT	TGGTGTTGCTCCTCTTCCC	Pool1	6595	6827
KM034562v1_1.7.140899	CGGTTTTGGAACTAATGAGACAGAGTAC	GGTCCGTTTGATACAGCTGTG	Pool2	6734	6960
KM034562v1_1.8.7962	AACACCACGGGAAAACTAATTTGGA	TTCCTTCCTTGACTGTGCACTT	Pool1	6864	7091
KM034562v1_1.8.11617	AACGGACCCAAAAACATCAGTG	TTGAGTTGCCTCAGAGATGTCAAG	Pool2	6995	7222
KM034562v1_1.8.209136	GGAAGGAAAGCTGCAGTGTC	AGTCAGCGCTCTTACTCGTG	Pool1	7125	7344
KM034562v1_1.8.85723	CAACTCAAGTTGGACAACATCACC	TAGTATTGGTAATTAAGCCTAGCTTCCC	Pool2	7262	7465
KM034562v1_1.8.204342	GCTGACTCCCTGGACCTC	TTGCAGCACCTTCATCCTGA	Pool1	7375	7596
KM034562v1_1.8.89003	CAATACTATTGCTGGAGTAGCAGGAC	CTCAGGAACAGTTGGAGAGCTT	Pool2	7511	7736
KM034562v1_1.8.270719	TGCTGCAATCGGATTGGC	CGGTCCCAAAATGTGGCATG	Pool1	7626	7835
KM034562v1_1.9.262042	TTCCTGAGAGCCACAACTGAG	ATACCTGCCGGTATCCATTGTC	Pool2	7771	7982
KM034562v1_1.9.250004	TGGGACCGGACTGCTGTA	TGCCGTGAAACAATCTGAAGAAAG	Pool1	7865	8069
KM034562v1_1.9.175749	GCAGGTATTGGAGTTACAGGTGTTATA	CATTGGCTATGTTTGAAGCTCCAG	Pool2	8023	8180
KM034562v1_1.9.225908	TCACGGCAAAACTCAACCTCA	GATTGTAGAATCAAGGATGAAGACTAAAGATAA	Pool1	8106	8318
KM034562v1_1.9.177909	GCCAATGTGATTCTAACTCCTTTAAACTC	TCTACCGGTTTGCAGATCTGATTATTAC	Pool2	8226	8399
KM034562v1_1.9.89770	CAATCATGACAGTTGTCTTTAATGAAAAAGGA	TCACCTCGATAATTCTCTCTGGATGAT	Pool1	8378	8594
KM034562v1_1.9.274099	TTTAGTTGTAACCTAACACACACAAAGC	AGGAACTGTTAATGGTTCAACTCTCTTC	Pool2	8456	8675
KM034562v1_1.9.126340	CGAGGTGAGTACCGTCAATCAA	CGAGTCCTTGGGTGCAGT	Pool1	8635	8862
KM034562v1_1.10.94624	CAGTTCCTCCAGCACCTAAAGAC	CCAGTGTTCTGCCGTCTTGAT	Pool2	8718	8949
KM034562v1_1.10.52601	AGGACTCGCGCTTAGCAAA	CAAGCCCTTCGCGCCTT	Pool1	8891	9089
KM034562v1_1.10.120641	CGAGACAAGACATCCGAACCATAG	TCGGTCCCATTGTTGCCATAG	Pool2	8995	9183
KM034562v1_1.10.56321	AGGGCTTGGGCAAGATCAG	CATGTCCCCGGGTTGGTT	Pool1	9117	9329
KM034562v1_1.10.187846	GGGACCGACAATCCCTAATTATGTTT	CGGAGTATTGTGATCAAGTAGAAGGTT	Pool2	9222	9398
KM034562v1_1.10.187265	GGGACATGCTCATGGTCTGAT	TGCTTGGCAGGTTATATTGCGA	Pool1	9360	9590
KM034562v1_1.10.48590	AGAGGTATGATATAACCCAAACATTGACC	CCTCCTCCACAACTTGAAGCA	Pool2	9565	9762
KM034562v1_1.10.170987	GCCAAGCATACCTCTTGCAC	TTTAGCTTTTCCGGTCACAATATACCT	Pool1	9624	9852

SM Table 2 Page 2 Primers, positions

KM034562v1_1.10.182045	GGAGGAGGTCTATGGTATTCGCTA	ATTTTGCTAAAAGGAGATGAAGAATGCC	Pool2	9799	9946
KM034562v1_1.11.187040	GATGAAGATTAATGCGGAGGTCTGA	GGAGTTAGCCCAGATTTATGCACT	Pool1	9909	10135
KM034562v1_1.11.270837	TATTTCAGGATAGTCCAGCTAGTGACA	GTGGGCTTCTTTTCAGCCAAT	Pool2	10003	10173
KM034562v1_1.11.147034	CTAACTCCACCAGGTCAACTCC	GACAACCCCTTTCTCCAGGTC	Pool1	10173	10389
KM034562v1_1.11.120212	CCCACCTACAACGAACATTACTTTGA	AACTTTCCACCCTTGAATAGTTTGACT	Pool2	10215	10437
KM034562v1_1.11.229840	GGGTTGTCTTAAGCGACCTCT	TGCCCACAGCGGTGATTC	Pool1	10423	10605
KM034562v1_1.11.213767	GGAAAGTTTATTGGGCTGGTATTGAGTT	AGCAGCCAATCAGAGATCAGAC	Pool2	10484	10702
KM034562v1_1.11.306918	TGTGGGCACTGAGAGTCATC	TGACACGTTGTGTTCGCATG	Pool1	10635	10748
KM034562v1_1.11.299831	TGGCTGCTAACAACCAACACTAA	TCTTGGAGCTCCACCAGAAAAC	Pool2	10739	10930
KM034562v1_1.12.39357	ACGTGTCAAGGAACAATTGAGC	TGTGGACTCATGAAGGAGGGA	Pool1	10782	11001
KM034562v1_1.12.156564	CTCCAAGAACCCGACAAATCG	AGAATTAAACTTTGCATTCGTGTCGAG	Pool2	10965	11045
KM034562v1_1.12.61658	AGTCCACACTGAAAGCATTTACACA	GAAGCTGGCTTACAGTGAGGA	Pool1	11039	11242
KM034562v1_1.12.141658	CTCAATAGTTAACTTGACATCTCTGCCT	CATGACCTGCGGGTTTAGTTACA	Pool2	11171	11395
KM034562v1_1.12.111167	CCAGCTTCCAAGTTGACACC	AAGCAGTTTTTCTTAATCTTCCTCAAGC	Pool1	11275	11496
KM034562v1_1.12.59978	AGGTCATGTGTGTTAGGTTTCACAAAT	GTATGATGAATACAACCCGCAAGC	Pool2	11437	11661
KM034562v1_1.12.12624	AACTGCTTATTGGGTCTTTCCGT	TGTCGCCACTGGTACATCAC	Pool1	11539	11770
KM034562v1_1.12.104887	CATCATACTCCCTTAATCCGCAACT	AATATTTCAGGAAGAGAGCATCTTGCAT	Pool2	11702	11901
KM034562v1_1.13.334030	TGGCGACATTGCCCATAGAT	AGCCAGGTCATACCAGAAAAACATT	Pool1	11802	12023
KM034562v1_1.13.257489	GGTGCTCAAGAAGACTGTGTTGAT	GGGATTCCTTGTGTGTTCAGTG	Pool2	11967	12175
KM034562v1_1.13.45122	ACCTGGCTATTTTAACTCGAAGGG	ACATCGGCAGTAGAAACAGAAACA	Pool1	12064	12263
KM034562v1_1.13.236582	GGAATCCCCCATGCTGCTATG	GAGAGTAAGTAATCTCCGCTCTGG	Pool2	12210	12410
KM034562v1_1.13.219986	GCCGATGTCTTGATAATGTGCAAA	GCCCTTCCTCTCGGTGTAC	Pool1	12303	12512
KM034562v1_1.13.352902	TTACTCTCCATATTAGGGTCTGATGGG	GGCGTCATCTCCAGCCTTATC	Pool2	12453	12644
KM034562v1_1.13.236099	GGAAGGGCCGATTCTTAACACA	AGGGCGTAATGCTTTTAGCAC	Pool1	12545	12762
KM034562v1_1.13.196664	GACGCCACAACAACTTTGTGA	GCAACGGAGGGAATTGATTTCTT	Pool2	12680	12911
KM034562v1_1.14.157796	CGCCCTATCGTGATTTTCGAGA	CTTTCTACTGCAGTAGCTCTGTCT	Pool1	12799	13028
KM034562v1_1.14.294573	TCCGTTGCCAATGATTAAAGAACTG	TACTCGAGTTTTTGCGCGTAGG	Pool2	12951	13177
KM034562v1_1.14.253147	GTAGAAAGGACATGCTGGGATGC	TTGCGAGTCGGATAAGGCAAT	Pool1	13067	13274
KM034562v1_1.14.174048	CTCGAGTATCTACTACCACAATATCGGA	CATGCTCACCGAAATCATCACTT	Pool2	13219	13421
KM034562v1_1.14.46501	ACTCGCAATGTTCAAACACTTTGT	ACGGTTGCAATATTCTATAAAAGGTGCT	Pool1	13311	13514
KM034562v1_1.14.306556	TGAGCATGCCACAGTTAGAGG	CCTTCAATCCCTCCCATATGACC	Pool2	13457	13686
KM034562v1_1.14.210333	GCAACCGTTGCTATGGTGTT	GCTGAGCGCAACTTAAAACCA	Pool1	13554	13772
KM034562v1_1.14.94569	ATTGAAGGACTGCAACAAAAACTCTG	GGGAAGACTGATAAAACGGTAATGC	Pool2	13727	13813
KM034562v1_1.15.144499	CGCTCAGCTGTGATGGGT	GTGCCATTCTTGTAGCCGTTT	Pool1	13803	14029
KM034562v1_1.15.220264	GCCTGTGGAATCTTTTTAAAACCTGATG	TGCAAGATTCTCACCGAAAAGAAC	Pool2	13951	14174
KM034562v1_1.15.242306	GGCACCATTGTCTGATGCAATT	CACCTGCGGTACCGCTAG	Pool1	14067	14286
KM034562v1_1.15.8965	AACCTCTGGATTTCGGAACAATATCATT	AAATGAAGTTAAGTCTTGCGACCCA	Pool2	14282	14504
KM034562v1_1.15.332596	TGGGAACTGCACTGCCAT	CTCGGCGTGCGTGAAAAT	Pool1	14468	14681
KM034562v1_1.15.192915	GACTTCGAAGACGAAATGGTTTGTAAG	GTTAAAGCCTCCGCCAGGATA	Pool2	14634	14861
KM034562v1_1.15.223312	GCGGGAAGCGATTGCAAATT	GTGGCTCCAATAAGAGGTCTCC	Pool1	14718	14950
KM034562v1_1.16.133565	CCCAAATAACTTGCACAGTTGATTTAGC	TGGGATTCCATCCCCGATAGT	Pool2	14909	15135
KM034562v1_1.16.243572	GGAGCCACACTCCCATGT	AAGGCTGCGGAAGGACATT	Pool1	14982	15208
KM034562v1_1.16.307167	TCAAGGACAGAAGATAAGATAGGGCAA	TTACTCATACGATTGGCCATGAAAGAAT	Pool2	15192	15412
KM034562v1_1.16.219189	GCAGCCTTAAGAGAGGCCATT	CCTCCTGAAAACTCACCTAAAGTGT	Pool1	15240	15469
KM034562v1_1.16.95014	ATGAGTAACTCAGCAACGCGAT	TCTCGGTATCTTGTTAAATCTAAATCCAATGT	Pool2	15454	15675
KM034562v1_1.16.139270	CCGGGAGGTACCAGCTCA	GCTAGCTCCCATCCAGATAAGTG	Pool1	15656	15831
KM034562v1_1.16.77747	ATCCTCTAAAAGGAGGACTCAATTGC	AAAGGCCCCAAAACTGTACAGAA	Pool2	15756	15970
KM034562v1_1.17.210449	GAGCTAGCTAAGACCATCATGCAA	ATGTTGGCTTAAGTATTCGCAATGAG	Pool1	15870	16094
KM034562v1_1.17.253663	GGGCCTTTGTAAGTTATTATCTTGGCA	CGCATCTGAGAGTCCTCTGTC	Pool2	16012	16197
KM034562v1_1.17.228423	GCCAACATTCAAACATGCAAGC	GCACCAGCAGTTCTACGATCT	Pool1	16134	16363
KM034562v1_1.17.123166	CAGATGCGGCCAGGTTATTTTTG	GAAATTGCTGGCTGTACTCTTACATG	Pool2	16233	16459
KM034562v1_1.17.239542	GCTGGTGCATGATTCATCAAGAC	TGCCATCATGTGGATCTCTGG	Pool1	16399	16618
KM034562v1_1.17.224467	GCAATTTCTTCCATGCGTCATTGG	GGTGCGTGTTCTCTTGTGGAA	Pool2	16501	16690
KM034562v1_1.17.319099	TGATGGCACTGAACGGAGTC	TGGCGTGTCCCTTGAGATAATG	Pool1	16651	16879
KM034562v1_1.17.255237	GGTCCGTCGTTCCAGTCATTT	CGGATCTCAATTGCCGTAAGTACTTT	Pool2	16734	16943
KM034562v1_1.18.33209	ACACGCCAATTAACGTCATCCA	CCAGCACCTTCTCCCTCTG	Pool1	16915	17080
KM034562v1_1.18.224597	GAGATCCGTCATTGATACCACAGTTTAT	AGCATCCTAGGAGTAGTCATTCCTG	Pool2	16989	17188
KM034562v1_1.18.278158	GGTGCTGGTGCCTTACTATTGA	CATCCATGGTTATAACCTCGACTTGC	Pool1	17113	17336
KM034562v1_1.18.366966	TGCTTCTACCTGTTATGTCAAAATTCCA	TCACTTAGAAAGACTTTAAGGACCACTG	Pool2	17237	17452
KM034562v1_1.18.141578	CATGGATGCAGAGACGACAGA	GGTGTGGCATCTTACGTGTAGT	Pool1	17375	17607
KM034562v1_1.18.42331	ACCGAGGGTATGTTATGGCTAAATG	GCGGATATAGCTTAAATGTAAATCGCA	Pool2	17506	17727
KM034562v1_1.18.142489	CCAAAACCATCTCAGTTGTAAGCAG	GTCGCTGTTGATTATAATCATTGGTCAATT	Pool1	17652	17872
KM034562v1_1.19.57829	ATATCCGCCTTGGTTTTCCATCAT	CCATGTCCCATTATGTTTTAATGCTTGT	Pool2	17770	17993
KM034562v1_1.19.149689	GCGACAAAGTCGGACTCAAAC	TCAGCCTTTCGATAAGCTTAACTTCA	Pool1	17918	18146
KM034562v1_1.19.157099	GGACATGGCAAGCTGAGTTTAAG	AATACTATGCACGGTTATTCAATCGAAC	Pool2	18036	18209
KM034562v1_1.19.46656	AGGCTGACAGGGCTTCTG	TGCAGCAGTAATATGTCACACATG	Pool1	18183	18416
KM034562v1_1.19.10982	AACGAAAGGAGTCCTTACATTATACACT	CCAAAGCACTATTCCATCTGGCA	Pool2	18368	18545
KM034562v1_1.19.115440	CTGCTGCAATGAGTCTAACGC	ATCACCATTTTTAAATCGACAAATTAAAGCAA	Pool1	18453	18629
KM034562v1_1.19.215226	TGCTTTGGTTGATAACACGACTTCTTAA	ATCATTAACACGAAGATTATTATCTAGTCTGC	Pool2	18588	18802
KM034562v1_1.19.62807	ATGGTGATAGCCTTAATCTTTGTGTAAAATAAG	TTAAATTTTTGTGTGCGACCATTTTTCC	Pool1	18686	18898
LMNA.ENCTRL.E3E4	CTGAGAACAGGCTGCAGACCATGAA	ACTCACGCTGCTTCCCATTGTCAA	Pool1		
LMNA.ENCTRL.E3E4.1	CTGAGAACAGGCTGCAGACCATGAA	ACTCACGCTGCTTCCCATTGTCAA	Pool2		
MYC.ENCTRL.E2E3	CCTGGTGCTCCATGAGGAGACA	GTGATCCAGACTCTGACCTTTTGCCA	Pool1		
MYC.ENCTRL.E2E3.1	CCTGGTGCTCCATGAGGAGACA	GTGATCCAGACTCTGACCTTTTGCCA	Pool2		
TBP.ENCTRL.E3E4	GACTCCCATGACCCCCATC	ACGAAGTGCAATGGTCTTTAGGT	Pool1		
TBP.ENCTRL.E3E4.1	GACTCCCATGACCCCCATC	ACGAAGTGCAATGGTCTTTAGGT	Pool2		
HMBS.ENCTRL.E8E9	CCAGCTCCCTGCGAAGAG	AGGATGATGGCACTGAACTCC	Pool1		
HMBS.ENCTRL.E8E9.1	CCAGCTCCCTGCGAAGAG	AGGATGATGGCACTGAACTCC	Pool2		
ITGB7.ENCTRL.E14E15	CCTATCTTGGATGATGGCTGGTGCAAAG	CGTGTGGTCTGCTCCCTTTTCTTG	Pool1		
ITGB7.ENCTRL.E14E15.1	CCTATCTTGGATGATGGCTGGTGCAAAG	CGTGTGGTCTGCTCCCTTTTCTTG	Pool2		

SM_Table 3 Ion Torrent Repeat Sequencing

Sample	genome_1	GenBank	genome_2	differences	nt _diff	indel
14729_EMLK	0807_C1_14729_EMLK	KU296392	1807_C2_14729_EMLK	0	0	0
14743_EMLK	0807_C1_14743_EMLK	KU296298	1807_C2_14743_EMLK	1	1	0
14750_EMLK	0807_C1_14750_EMLK	KU296459	1807_C2_14750_EMLK	0	0	0
KT4609	1706_C1_KT4609	KU296622	0806_C2_KT4609	0	0	0
MK0369	1708_C1_MK0369R	KU296413	1007_C2_MK0369	1	0	1
MK0375	1708_C1_MK0375R	KU296606	0507_C2_MK0375	1	0	1
MK0384	1708_C1_MK0384R	KU296628	0507_C2_MK0384	1	0	1
MK0388	1708_C1_MK0388R	KU296630	1007_C2_MK0388	1	0	1
MK0425	1708_C1_MK0425R	KU296379	0507_C2_MK0425	1	0	1
MK0431	1708_C1_MK0431R	KU296621	1007_C2_MK0431	1	0	1
MK0434	2208_C1_MK0434R2	KU296554	1708_C1_MK0434R	2	0	2
MK0491	1708_C1_MK0491R	KU296510	0208_C2_MK0491R	3	0	3
MK0531	1708_C1_MK0531R	KU296448	0208_C2_MK0531R	1	0	1
MK0607	1007_C2_MK0607	KU296335	1708_C1_MK0607R	1	0	1
MK1036	1708_C2_MK1036R	KU296834	1007_C2_MK1036	1	1	0
MK1888	1007_C2_MK1888	KU296708	1706_C1_MK1888	0	0	0
MK2341	1805_C1_MK2341	KU296494	1805_C1_MK2341R	0	0	0
MK2371	1105_C2_MK2371	KU296397	1805_C1_MK2371	6	0	6
MK2789	2208_C1_MK2789R3	KU296613	1708_C2_MK2789R2	4	0	4
MK2790	1105_C1_MK2790	KU296452	1708_C2_MK2790R	2	0	2
MK2826	0807_C2_MK2826	KU296405	1708_C2_MK2826R	2	0	2
PL4105	1007_C2_PL4105	KU296694	2906_C1_PL4105	0	0	0
PL5133	2605_C2_PL5133R	KU296489	2605_C2_PL5133	0	0	0
PL6049	0205_C2_PL6049	KU296743	0305_C1_PL6049	2	0	2
PL7376	0806_C1_PL7376	KU296740	0106_C2_PL7376	10	0	10
PL7401	0806_C1_PL7401	KU296819	0106_C2_PL7401	7	0	7
PL7577	0807_C1_PL7577	KU296385	0806_C1_PL7577	1	0	1
PL9150	1807_C1_PL9150R	KU296522	1507_C1_PL9150	0	0	0

SM_Table 4 MinION Ion Torrent overlap

Sample	Minion genome	Ion Torrent genome	GenBank	differences	nt differences	indels	nt overlap	nt after N removal	nts missing in Minion/IT overlap
020380	EBOV|KAM020380|MinION|SLE|Kambia--|2015-08-28	0109_C2_020380_EMLK	KU296775	5	0	5	18886	17796	1090
508124	EBOV|KAM0508124|MinION|SLE|Kambia--|2015-09-04	0809_C1_020484_EMLK	KU296462	5	0	5	18887	17795	1092
20524	EBOV|KAM020524|MinION|SLE|Kambia--|2015-09-08	1009_C1_20524_EMLK	KU296424	5	0	5	18746	17680	1066
20573	EBOV|KAM020573|MinION|SLE|Kambia--|2015-09-09	1209_C1_020573_EMLK	KU296654	4	0	4	18820	17749	1071
13013	EBOV|MK013013|MinION|GUI|Bombali--|2015-09-15	1309_C2_MK13013	KU296454	1	0	1	18874	15960	2914

SM Table 5 Page 1 Residual Primers

Genome_ID	number_reads	reads_examined	average_read_length	no_reads_primer_ends	fraction
0106_C2_KT2315	46319	10000	197.086	33	0.001
0106_C2_KT2316	49331	10000	193.395	23	0
0106_C2_KT2317	123234	10000	192.881	27	0
0106_C2_KT2321	98831	10000	194.351	25	0
0106_C2_KT2449	156082	10000	180.951	7	0
0106_C2_KT2641	48461	10000	194.404	34	0.001
0106_C2_PL7375	47465	10000	183.56	14	0
0109_C2_020380_EMLK_ABCDEF	346228	10000	202.716	22	0
0205_C1_PL5249	151610	10000	161.926	9	0
0205_C1_PL5260	347623	10000	184.169	24	0
0205_C1_PL5294	504789	10000	184.149	6	0
0205_C1_PL5361	135185	10000	186.824	20	0
0205_C1_PL5428	527028	10000	166.992	7	0
0205_C2_PL5674	458553	10000	183.555	37	0
0205_C2_PL5801	633677	10000	184.277	8	0
0205_C2_PL5803	716659	10000	200.306	27	0
0205_C2_PL5868	538598	10000	203.316	35	0
0205_C2_PL5950	58745	10000	192.022	32	0.001
0205_C2_PL6049	140295	10000	189.475	26	0
0205_C2_PL6070	339601	10000	195.691	35	0
0205_C2_PL6086	445542	10000	176.155	15	0
0205_C2_PL6158	208506	10000	188.908	28	0
0208_C1_77667_EMLK	133677	10000	192.371	22	0
0208_C1_77669_EMLK	65256	10000	201.881	36	0.001
0208_C1_77670_EMLK	137666	10000	198.735	31	0
0208_C1_77673_EMLK	97509	10000	197.121	15	0
0208_C1_77675_EMLK	81740	10000	201.123	39	0
0208_C1_77676_EMLK	385781	10000	202.268	14	0
0208_C1_77677_EMLK	148594	10000	197.601	21	0
0208_C1_77679_EMLK	89922	10000	200.243	37	0
0208_C1_77681_EMLK	202379	10000	191.619	15	0
0208_C1_MK10128R	491743	10000	206.166	14	0
0208_C1_MK10173	327169	10000	203.247	9	0
0208_C2_KT4828R	209631	10000	197.897	23	0
0208_C2_KT4935R	266894	10000	196.563	15	0
0208_C2_KT5022R	193794	10000	198.367	30	0
0208_C2_KT5245R	162930	10000	197.671	15	0
0305_C1_PL4651	174270	10000	185.104	22	0
0305_C1_PL4696	447181	10000	195.255	28	0
0305_C1_PL4844	153411	10000	192.158	28	0
0305_C1_PL4932	389248	10000	203.053	44	0
0305_C1_PL5025	213904	10000	193.526	17	0
0305_C1_PL5201	235681	10000	190.391	26	0
0305_C1_PL5202	255412	10000	192.86	21	0
0305_C1_PL5228	96948	10000	189.055	25	0
0305_C2_PL4570	151623	10000	188.324	20	0
0507_C1_PL4226	95765	10000	167.721	12	0
0507_C1_PL4248	136352	10000	162.775	13	0
0507_C1_PL4249	57518	10000	182.548	30	0.001
0507_C1_PL4283	185502	10000	168.411	3	0
0507_C1_PL4322	151527	10000	177.689	22	0
0507_C1_PL4443	177230	10000	187.3	11	0
0507_C1_PL4671	206360	10000	161.671	6	0
0507_C1_PL4679	26770	10000	175.122	33	0.001
0507_C1_PL4680	65094	10000	156.503	20	0
0507_C1_PL5179	80191	10000	162.743	17	0
0507_C2_MK0390	288388	10000	163.936	18	0
0507_C2_MK0392	34881	10000	168.661	34	0.001
0507_C2_MK0664	23098	10000	191.551	16	0.001
0507_C2_MK0725	43435	10000	176.082	23	0.001
0806_C1_KT4677	450542	10000	194.715	22	0
0806_C1_KT4695	601671	10000	193.328	18	0
0806_C1_KT4715	115249	10000	159.126	8	0
0806_C1_KT4730	163832	10000	190.053	18	0
0806_C1_KT4801	63311	10000	181.414	37	0.001
0806_C1_KT5073	385189	10000	192.098	27	0

SM Table 5 Page 2 Residual Primers

0806_C1_MK1040	37017	10000	186.817	20	0.001
0806_C1_MK1079	224813	10000	182.489	6	0
0806_C1_PL7376	214565	10000	188.426	29	0
0806_C1_PL7401	106246	10000	185.232	31	0
0806_C1_PL7429	110505	10000	184.34	32	0
0806_C1_PL7451	42020	10000	182.574	31	0.001
0806_C1_PL7496	13788	10000	186.177	31	0.002
0806_C1_PL7511	177224	10000	204.646	17	0
0806_C1_PL7551	154830	10000	199.097	26	0
0806_C2_KT2669	33903	10000	195.885	37	0.001
0806_C2_KT2810	110254	10000	193.917	12	0
0806_C2_KT4054	101160	10000	191.013	25	0
0806_C2_KT4553	317323	10000	197.099	36	0
0806_C2_KT4555	313915	10000	198.582	20	0
0806_C2_KT4622	53650	10000	195.582	27	0.001
0806_C2_KT4631	152688	10000	191.369	19	0
0806_C2_KT4663	60698	10000	202.357	29	0
0806_C2_PL1387	233153	10000	200.529	42	0
0806_C2_PL2101	34243	10000	190.474	31	0.001
0806_C2_PL2668	201134	10000	196.372	11	0
0806_C2_PL2921	401957	10000	195.824	63	0
0807_C1_14729_EMLK	113485	10000	152.02	14	0
0807_C1_14743_EMLK	184675	10000	174.838	13	0
0807_C1_14748_EMLK	179951	10000	159.179	23	0
0807_C1_14750_EMLK	112090	10000	178.333	10	0
0807_C1_PL3984	26660	10000	181.116	26	0.001
0807_C1_PL4119	21393	10000	178.954	41	0.002
0807_C1_PL4121	87321	10000	177.547	30	0
0807_C1_PL4123	33699	10000	188.869	30	0.001
0807_C1_PL4125	31513	10000	191.025	46	0.001
0807_C1_PL4126	270577	10000	185.54	12	0
0807_C1_PL4763	20006	10000	183.598	38	0.002
0807_C1_PL4784	11773	10000	190.566	34	0.003
0807_C1_PL4785	45067	10000	184.786	11	0
0807_C1_PL4804	104623	10000	174.049	23	0
0807_C1_PL4813	78096	10000	170.231	28	0
0807_C1_PL4864	73265	10000	176.667	32	0
0807_C1_PL4865	81358	10000	188.768	16	0
0807_C1_PL4866	119461	10000	167.937	21	0
0807_C1_PL7577	75620	10000	182.837	41	0.001
0807_C1_PL8429	68849	10000	189.839	47	0.001
0807_C1_PL8486	55661	10000	191.347	35	0.001
0807_C1_PL8630	124586	10000	179.559	40	0
0807_C2_MK2826	228811	10000	199.965	30	0
0809_C1_020484_EMLK	149022	10000	197.79	10	0
0809_C1_19560_EMLK	277907	10000	201.273	5	0
0908_C1_KT7105	303738	10000	206.325	18	0
1007_C1_MK1782	164392	10000	184.341	10	0
1007_C1_PL4028	55984	10000	180.972	39	0.001
1007_C1_PL4059	124683	10000	161.346	11	0
1007_C1_PL4094	168033	10000	177.853	13	0
1007_C1_PL4187	86462	10000	191.811	44	0.001
1007_C1_PL4190	191562	10000	158.687	23	0
1007_C1_PL4346	297643	10000	198.158	10	0
1007_C2_MK0020	342466	10000	201.39	16	0
1007_C2_MK0024	12587	10000	196.413	18	0.001
1007_C2_MK0045	768158	10000	202.85	37	0
1007_C2_MK0085	100159	10000	197.813	31	0
1007_C2_MK0391	36775	10000	175.959	36	0.001
1007_C2_MK0607	121821	10000	177.032	31	0
1007_C2_MK0894	68223	10000	197.338	40	0.001
1007_C2_MK1000	193304	10000	184.401	28	0
1007_C2_MK1014	904024	10000	202.85	7	0
1007_C2_MK1888	19536	10000	195.948	39	0.002
1007_C2_PL4105	53099	10000	173.819	11	0
1009_C1_20524_EMLK	51293	10000	177.227	18	0
1009_C1_20525_EMLK	359698	10000	185.752	4	0

SM Table 5 Page 3 Residual Primers

1009_C1_20547_EMLK	325524	10000	198.56	11	0
1105_C1_MK2405	93927	10000	195.572	31	0
1105_C1_MK2427	595874	10000	199.417	32	0
1105_C1_MK2431	713158	10000	197.785	28	0
1105_C1_MK2436	360883	10000	189.713	30	0
1105_C1_MK2673	697083	10000	207.534	47	0
1105_C1_MK2790	412016	10000	178.033	7	0
1105_C2_MK2334	368561	10000	174.75	12	0
1105_C2_MK2369	97043	10000	193.31	30	0
1105_C2_MK2370	253780	10000	189.249	35	0
1105_C2_MK2371	622310	10000	196.877	8	0
1105_C2_MK2383R	274991	10000	187.525	12	0
1209_C1_020573_EMLK	284299	10000	185.367	26	0
1309_C2_MK13013	390853	10000	191.835	5	0
1507_C1_13204_EMLH	50556	10000	168.888	23	0
1507_C1_13268_EMLH	59157	10000	170.524	10	0
1507_C1_13275_EMLH	36409	10000	184.558	24	0.001
1507_C1_18538_EMLH	84120	10000	177.551	4	0
1507_C1_18596_EMLH	37528	10000	173.125	35	0.001
1507_C1_18636_EMLH	252561	10000	173.043	6	0
1507_C1_18647_EMLH	25358	10000	182.61	38	0.001
1507_C1_18648_EMLH	146124	10000	170.034	17	0
1507_C1_18649_EMLH	113710	10000	199.272	12	0
1507_C1_18650_EMLH	232371	10000	182.598	13	0
1507_C1_18660_EMLH	203702	10000	177.853	11	0
1507_C1_18706_EMLH	130273	10000	170.932	20	0
1507_C2_15543_EMLK	87307	10000	167.228	26	0
1507_C2_15661_EMLK	50410	10000	176.167	35	0.001
1507_C2_15674_EMLK	131854	10000	174.352	24	0
1507_C2_15686_EMLK	48122	10000	164.998	21	0
1507_C2_19521_EMLK	62190	10000	184.403	27	0
1507_C2_MK0363	143338	10000	170.879	28	0
1507_C2_MK0393	72375	10000	154.584	19	0
1507_C2_MK0574	67997	10000	181.586	36	0.001
1507_C2_MK0575	248564	10000	164.017	12	0
1507_C2_MK0584	344074	10000	198.618	5	0
1507_C2_MK0709	89472	10000	175.105	22	0
1507_C2_MK0857	35269	10000	190.984	32	0.001
1507_C2_MK0864	40511	10000	187.245	28	0.001
1507_C2_MK0865	239399	10000	175.107	31	0
1507_C2_MK0866	64463	10000	189.817	46	0.001
1507_C2_MK0867	59134	10000	169.243	30	0.001
1507_C2_MK0868	171062	10000	172.963	9	0
1507_C2_MK0869	321483	10000	161.419	3	0
1507_C2_MK0879	75971	10000	191.145	42	0.001
1507_C2_MK0888	93847	10000	181.156	36	0
1507_C2_MK0889	281715	10000	131.151	8	0
1507_C2_MK0966	44842	10000	168.096	16	0
1706_C1_KT4609	398968	10000	205.924	28	0
1706_C1_PL7604	465840	10000	199.825	20	0
1706_C1_PL7605	67905	10000	194.178	26	0
1706_C1_PL7626	743962	10000	197.333	4	0
1706_C1_PL7678	96548	10000	190.635	22	0
1706_C1_PL7685	144707	10000	198.973	22	0
1706_C1_PL7709	504415	10000	204.282	20	0
1706_C1_PL7773	120218	10000	190.539	18	0
1706_C1_PL7774	79634	10000	196.512	38	0
1706_C1_PL7775	19500	10000	187.336	30	0.002
1706_C1_PL7792	228177	10000	204.908	45	0
1706_C1_PL7801	109036	10000	187.455	53	0
1706_C1_PL7820	59279	10000	188.3	69	0.001
1708_C1_12634_EMLH	59817	10000	193.093	30	0.001
1708_C1_12705_EMLH	46987	10000	194.134	33	0.001
1708_C1_13031_EMLH	182079	10000	203.149	27	0
1708_C1_MK0367R	22044	10000	202.139	35	0.002
1708_C1_MK0369R	201680	10000	197.852	30	0
1708_C1_MK0375R	308366	10000	200.273	27	0

SM Table 5 Page 4 Residual Primers

1708_C1_MK0379R	125516	10000	196.003	44	0
1708_C1_MK0384R	489273	10000	204.995	21	0
1708_C1_MK0388R	64101	10000	200.151	33	0.001
1708_C1_MK0425R	518505	10000	207.57	23	0
1708_C1_MK0431R	76804	10000	198.069	26	0
1708_C1_MK0491R	26719	10000	200.569	24	0.001
1708_C1_MK0531R	142807	10000	201.012	38	0
1708_C1_MK0533R	8031	8031	210.743	30	0.003735525
1708_C1_MK0592R	66133	10000	201.561	40	0.001
1708_C1_MK0704R	24714	10000	201.461	35	0.001
1708_C1_MK0723R	249376	10000	196.669	18	0
1708_C1_MK0766R	24376	10000	201.16	33	0.001
1708_C1_MK0849R	356029	10000	207.131	24	0
1708_C2_MK1036R	115367	10000	199.926	18	0
1708_C2_MK1156R	214301	10000	200.094	19	0
1708_C2_MK2008R2	196879	10000	184.262	9	0
1708_C2_MK2282R	66449	10000	205.054	35	0.001
1708_C2_MK2283R	224606	10000	203.49	26	0
1708_C2_MK2307R	24704	10000	204.165	30	0.001
1708_C2_MK2506R	137888	10000	199.322	27	0
1708_C2_MK2656R2	38012	10000	201.334	39	0.001
1708_C2_MK2717R	122486	10000	205.316	40	0
1708_C2_MK2900R2	26837	10000	199.143	24	0.001
1804_C1_MK2570	185701	10000	195.327	9	0
1805_C1_MK2255	145991	10000	191.281	26	0
1805_C1_MK2341	142325	10000	191.594	9	0
1805_C1_MK2382	29678	10000	189.539	31	0.001
1805_C1_MK2395	60770	10000	189.302	21	0
1805_C1_MK2397	170588	10000	191.783	7	0
1805_C1_MK2398	69078	10000	189.909	25	0
1805_C1_MK2710	125091	10000	188.216	17	0
1805_C1_MK2724	92113	10000	194.028	28	0
1805_C1_MK2788	68407	10000	197.819	28	0
1805_C1_MK2825	191558	10000	196.275	24	0
1805_C1_MK3043	155025	10000	202.846	25	0
1805_C1_MK3462	42655	10000	193.297	38	0.001
1805_C1_MK3479	36357	10000	195.659	41	0.001
1807_C1_14859_EMLK	24991	10000	202.833	28	0.001
1807_C1_14888_EMLK	65057	10000	189.951	20	0
1807_C1_15000_EMLK	80953	10000	194.517	11	0
1807_C1_15274_EMLK	71375	10000	175.97	20	0
1807_C1_15314_EMLK	120918	10000	207.226	32	0
1807_C1_15323_EMLK	38768	10000	203.808	36	0.001
1807_C1_15327_EMLK	48830	10000	204.52	29	0.001
1807_C1_15338_EMLK	231369	10000	208.696	23	0
1807_C1_15349_EMLK	84380	10000	202.221	28	0
1807_C1_15360_EMLK	59357	10000	201.453	36	0.001
1807_C1_15373_EMLK	62752	10000	198.668	20	0
1807_C1_15421_EMLK	32472	10000	189.782	34	0.001
1807_C1_15470_EMLK	76280	10000	180.309	30	0
1807_C1_77724_EMLK	41446	10000	170.488	9	0
1807_C1_MK0464	95209	10000	172.158	58	0.001
1807_C1_MK0551	237743	10000	170.778	29	0
1807_C1_PL8310	350515	10000	204.722	28	0
1807_C1_PL8780	407439	10000	208.655	27	0
1807_C1_PL8896	53773	10000	206.939	56	0.001
1807_C1_PL9150R	427699	10000	204.333	28	0
1807_C1_PL9192R	40559	10000	208.202	4	0
1807_C1_PL9199R	513160	10000	192.601	13	0
1807_C2_10354_EMLK	119074	10000	191.043	36	0
1807_C2_10356_EMLK	66174	10000	195.316	24	0
1807_C2_10394_EMLK	47003	10000	202.441	27	0.001
1807_C2_10398_EMLK	130871	10000	195.93	45	0
1807_C2_10402_EMLK	90537	10000	193.257	30	0
1807_C2_10415_EMLK	83915	10000	196.611	19	0
1807_C2_10454_EMLK	96266	10000	179.083	17	0
1807_C2_10455_EMLK	120057	10000	196.697	22	0

SM Table 5 Page 5 Residual Primers

1807_C2_14521_EMLK	244813	10000	201.405	14	0
1807_C2_14549_EMLK	173117	10000	188.044	9	0
1807_C2_14687_EMLK	78598	10000	200.965	18	0
1807_C2_14723_EMLK	131338	10000	200.826	19	0
1807_C2_14749_EMLK	85107	10000	171.407	11	0
1807_C2_14763_EMLK	118908	10000	207.081	24	0
1807_C2_14776_EMLK	70981	10000	206.346	38	0.001
1807_C2_14782_EMLK	143320	10000	206.364	35	0
1807_C2_14795_EMLK	90333	10000	200.939	22	0
1807_C2_14830_EMLK	226244	10000	197.644	27	0
1807_C2_14846_EMLK	62760	10000	201.872	29	0
2005_C2_PL6656	51713	10000	191.946	38	0.001
2005_C2_PL7053	40617	10000	188.275	35	0.001
2005_C2_PL7055	19260	10000	189.814	28	0.001
2104_C1_MK2342	893472	10000	193.554	23	0
2104_C1_MK2343	430741	10000	183.377	14	0
2104_C1_MK2344	748037	10000	185.338	26	0
2104_C1_MK2362	327017	10000	185.701	18	0
2104_C1_MK2364	253279	10000	189.52	36	0
2104_C1_MK2365	497785	10000	187.667	18	0
2104_C1_MK2367	327903	10000	190.646	20	0
2104_C1_MK2437	369443	10000	188.604	21	0
2104_C1_MK2449	181505	10000	193.328	34	0
2104_C2_MK2366	185059	10000	187.277	14	0
2104_C2_MK2447	559412	10000	193.601	8	0
2104_C2_MK2448	339427	10000	187.637	22	0
2104_C2_MK2850R	102448	10000	187.496	23	0
2104_C2_MK2938	201839	10000	189.332	23	0
2104_C2_MK2983	211071	10000	189.039	23	0
2104_C2_MK3050	96197	10000	191.023	38	0
2104_C2_MK4165	404083	10000	192.955	19	0
2208_C1_MK0433R2	11760	10000	202.224	28	0.002
2208_C1_MK0434R2	21674	10000	201.99	35	0.002
2208_C1_MK0549R2	9586	9586	211.804	28	0.002920926
2208_C1_MK2789R3	40648	10000	205.534	32	0.001
2208_C1_MK2918R	240086	10000	207.838	44	0
2208_C1_MK3183R2	196607	10000	203.874	12	0
2208_C1_PL4127R	103879	10000	203.491	27	0
2208_C1_PL4169R	77758	10000	204.196	32	0
2208_C1_PL4197R	106602	10000	208.071	39	0
2208_C1_PL4203R	63475	10000	203.095	25	0
2208_C1_PL4256R	315032	10000	199.12	26	0
2208_C1_PL4284R	46676	10000	203.811	35	0.001
2208_C1_PL4292R	201142	10000	201.884	40	0
2208_C1_PL4432R	47167	10000	202.881	20	0
2208_C1_PL4736R	60127	10000	203.019	26	0
2208_C2_12615_EMLH	79710	10000	201.759	22	0
2208_C2_15207R_EMLK	39829	10000	199.561	28	0.001
2208_C2_15438R_EMLK	67263	10000	201.487	42	0.001
2208_C2_18638R_EMLH	67866	10000	202.25	16	0
2208_C2_18642R_EMLH	152575	10000	200.094	14	0
2208_C2_18659R_EMLH	64864	10000	204.593	41	0.001
2208_C2_18687R_EMLH	80798	10000	204.895	17	0
2208_C2_19560R_EMLK	112044	10000	203.824	29	0
2208_C2_356_EMLH	16439	10000	205.212	29	0.002
2208_C2_651_EMLH	46490	10000	203.124	46	0.001
2208_C2_75711_EMLH	69669	10000	202.709	34	0
2208_C2_75722_EMLH	230514	10000	202.228	20	0
2208_C2_75731_EMLH	29257	10000	198.627	45	0.002
2208_C2_75779_EMLH	41534	10000	202.029	42	0.001
2208_C2_762_EMLH	29589	10000	201.406	33	0.001
2208_C2_PL7483R	100825	10000	200.271	33	0
2208_C2_PL7834R2	44307	10000	197.974	27	0.001
2507_C1_10012_EMLK	77775	10000	192.881	18	0
2507_C1_10022_EMLK	50196	10000	191.786	24	0
2507_C1_10023_EMLK	592308	10000	196.023	9	0
2507_C1_10025_EMLK	117149	10000	200.643	20	0

SM Table 5 Page 6 Residual Primers

2507_C1_77725_EMLK	58853	10000	195.911	47	0.001
2507_C1_77727_EMLK	126912	10000	187.45	15	0
2507_C1_77737_EMLK	102149	10000	196.288	27	0
2507_C1_77742_EMLK	136885	10000	193.705	28	0
2507_C1_77746_EMLK	200956	10000	185.115	20	0
2507_C1_77747_EMLK	109374	10000	194.279	26	0
2507_C1_77748_EMLK	201977	10000	186.247	22	0
2507_C1_77749_EMLK	61108	10000	200.867	43	0.001
2507_C1_77750_EMLK	607706	10000	200.244	27	0
2507_C1_77751_EMLK	525260	10000	195.602	9	0
2507_C1_77753_EMLK	166888	10000	192.592	19	0
2507_C1_77756_EMLK	213934	10000	180.191	7	0
2507_C1_77767_EMLK	50576	10000	194.771	39	0.001
2507_C1_77768_EMLK	35831	10000	199.527	29	0.001
2507_C1_77795_EMLK	72874	10000	199.407	12	0
2507_C1_MK8878	217200	10000	194.085	8	0
2507_C2_10027_EMLK	57128	10000	204.217	42	0.001
2507_C2_10038_EMLK	264053	10000	195.62	20	0
2507_C2_10040_EMLK	125209	10000	194.105	29	0
2507_C2_10049_EMLK	51460	10000	196.903	23	0
2507_C2_10065_EMLK	32078	10000	197.365	26	0.001
2507_C2_10066_EMLK	159762	10000	202.386	28	0
2507_C2_10080_EMLK	365161	10000	205.358	42	0
2507_C2_10082_EMLK	71503	10000	191.869	35	0
2507_C2_10084_EMLK	32189	10000	205.987	33	0.001
2507_C2_10112_EMLK	63861	10000	198.08	34	0.001
2507_C2_10113_EMLK	81328	10000	202.484	30	0
2507_C2_10123_EMLK	59991	10000	193.667	8	0
2507_C2_10132_EMLK	58777	10000	203.675	18	0
2507_C2_10134_EMLK	118417	10000	200.38	8	0
2507_C2_10142_EMLK	23794	10000	200.545	32	0.001
2507_C2_10212_EMLK	272964	10000	206.516	23	0
2507_C2_10244_EMLK	256847	10000	205.506	35	0
2507_C2_10247_EMLK	31205	10000	199.524	37	0.001
2507_C2_10248_EMLK	135138	10000	204.203	40	0
2507_C2_10278_EMLK	30387	10000	201.825	31	0.001
2507_C2_10352_EMLK	61828	10000	204.847	42	0.001
2507_C2_10353_EMLK	30282	10000	201.323	27	0.001
2507_C2_77722_EMLK	120307	10000	189.518	27	0
2507_C2_77723_EMLK	180695	10000	204.417	16	0
2605_C1_MK0885	221035	10000	160.929	9	0
2605_C1_MK1197	447490	10000	137.223	9	0
2605_C1_MK1202	222197	10000	148.747	4	0
2605_C1_MK1320	95463	10000	114.618	11	0
2605_C2_KT5226	193171	10000	184.594	22	0
2605_C2_KT5233	151897	10000	185.181	17	0
2605_C2_KT5243	163152	10000	177.135	11	0
2605_C2_PL5133R	228810	10000	199.402	22	0
2605_C2_PL5275	273300	10000	178.901	21	0
2605_C2_PL5534	174124	10000	173.146	32	0
2605_C2_PL7136b	147754	10000	187.734	33	0
2607_C1_12004_EMLH	18862	10000	201.234	27	0.001
2607_C1_12106_EMLH	413694	10000	209.701	24	0
2607_C1_12112_EMLH	41428	10000	200.838	29	0.001
2607_C1_12161_EMLH	569287	10000	209.536	23	0
2607_C1_12178_EMLH	185235	10000	199.949	21	0
2607_C1_12179_EMLH	148493	10000	201.507	40	0
2607_C1_592_EMLH	42335	10000	200.978	41	0.001
2607_C1_611_EMLH	58898	10000	206.249	57	0.001
2607_C1_614_EMLH	245358	10000	206.386	42	0
2607_C1_632_EMLH	77426	10000	199.246	54	0.001
2607_C1_634_EMLH	34251	10000	201.679	40	0.001
2607_C1_704_EMLH	53610	10000	203.421	48	0.001
2607_C1_705_EMLH	109007	10000	204.896	35	0
2607_C1_731_EMLH	61447	10000	199.621	33	0.001
2607_C1_732_EMLH	79835	10000	198.532	34	0
2607_C1_735_EMLH	98639	10000	199.683	23	0

SM Table 5 Page 7 Residual Primers

2607_C1_741_EMLH	114916	10000	203.452	37	0
2607_C1_793_EMLH	185393	10000	206.002	22	0
2607_C1_PL4022R	215639	10000	202.709	10	0
2607_C1_PL4052R	262122	10000	200.4	14	0
2607_C1_PL4330R	180414	10000	178.291	20	0
2607_C2_12180_EMLH	319546	10000	206.792	42	0
2607_C2_12181_EMLH	41708	10000	180.847	20	0
2607_C2_12189_EMLH	162376	10000	204.149	24	0
2607_C2_12192_EMLH	230552	10000	207.925	26	0
2607_C2_12193_EMLH	75392	10000	205.678	36	0
2607_C2_12229_EMLH	27718	10000	205.653	33	0.001
2607_C2_12242_EMLH	290627	10000	208.74	36	0
2607_C2_12257_EMLH	152968	10000	207.98	44	0
2607_C2_12275_EMLH	110656	10000	202.955	28	0
2607_C2_12600_EMLH	62762	10000	203.494	28	0
2607_C2_12628_EMLH	49443	10000	201.285	24	0
2607_C2_12666_EMLH	100021	10000	201.598	27	0
2607_C2_12757_EMLH	82780	10000	195.687	23	0
2607_C2_12767_EMLH	26165	10000	191.399	36	0.001
2607_C2_12781_EMLH	206733	10000	207.757	36	0
2607_C2_12851_EMLH	61558	10000	204.482	37	0.001
2607_C2_12854_EMLH	99764	10000	204.234	16	0
2607_C2_12855_EMLH	66055	10000	204.55	33	0
2607_C2_12872_EMLH	67969	10000	203.561	29	0
2607_C2_12916_EMLH	15460	10000	205.359	43	0.003
2607_C2_12934_EMLH	95022	10000	205.073	29	0
2607_C2_12936_EMLH	355945	10000	203.086	15	0
2807_C1_361_EMLH	117414	10000	197.73	29	0
2807_C1_362_EMLH	368080	10000	203.49	27	0
2807_C1_375_EMLH	71983	10000	202.578	29	0
2807_C1_380_EMLH	85794	10000	200.177	23	0
2807_C1_389_EMLH	101031	10000	194.729	29	0
2807_C1_402_EMLH	88031	10000	200.475	28	0
2807_C1_405_EMLH	62417	10000	200.537	25	0
2807_C1_408_EMLH	547057	10000	204.766	14	0
2807_C1_413_EMLH	562050	10000	205.375	20	0
2807_C1_466_EMLH	185891	10000	193.148	16	0
2807_C1_468_EMLH	242827	10000	198.021	11	0
2807_C1_470_EMLH	175225	10000	188.978	7	0
2807_C1_473_EMLH	84258	10000	188.757	13	0
2807_C1_480_EMLH	484971	10000	206.229	26	0
2807_C1_75674_EMLH	23216	10000	201.586	26	0.001
2807_C1_75685_EMLH	51997	10000	199.01	26	0.001
2807_C1_75688_EMLH	7808	7808	208.565	24	0.00307377
2807_C1_75705_EMLH	107962	10000	201.136	39	0
2807_C1_75707_EMLH	51934	10000	197.701	27	0.001
2807_C1_75727_EMLH	114209	10000	201.013	43	0
2807_C1_75728_EMLH	131335	10000	200.795	37	0
2807_C1_75750_EMLH	437301	10000	204.197	20	0
2807_C1_75789_EMLH	55158	10000	199.371	41	0.001
2807_C2_489_EMLH	145368	10000	203.442	37	0
2807_C2_490_EMLH	74468	10000	200.092	23	0
2807_C2_491_EMLH	103457	10000	201.013	19	0
2807_C2_505_EMLH	104586	10000	201.784	36	0
2807_C2_508_EMLH	47171	10000	202.115	24	0.001
2807_C2_511_EMLH	73060	10000	199.729	46	0.001
2807_C2_518_EMLH	79283	10000	201.693	34	0
2807_C2_539_EMLH	147054	10000	199.011	33	0
2807_C2_554_EMLH	48415	10000	201.394	36	0.001
2807_C2_567_EMLH	72177	10000	193.605	38	0.001
2807_C2_77685_EMLK	124011	10000	182.857	29	0
2807_C2_77687_EMLK	272302	10000	160.587	14	0
2807_C2_77688_EMLK	179661	10000	187.308	32	0
2807_C2_77693_EMLK	258868	10000	204.303	18	0
2807_C2_77696_EMLK	49063	10000	202.895	38	0.001
2807_C2_77706_EMLK	186953	10000	201.322	35	0
2807_C2_77710_EMLK	75698	10000	204.539	30	0

SM Table 5 Page 8 Residual Primers

2807_C2_77711_EMLK	47926	10000	197.146	24	0.001
2807_C2_77712_EMLK	74644	10000	196.548	36	0
2807_C2_77716_EMLK	120716	10000	197.578	21	0
2807_C2_MK1355R	220943	10000	125.712	8	0
2906_C1_PL4194	123385	10000	186.248	9	0
2906_C1_PL4347	207724	10000	190.95	2	0
2906_C1_PL4483	85992	10000	167.789	29	0
2906_C1_PL4561	118616	10000	168.927	9	0
2906_C1_PL4569	80107	10000	173.457	23	0
2906_C1_PL4872	158466	10000	183.429	17	0
2906_C1_PL4886	280791	10000	194.069	15	0
2906_C1_PL5001	252347	10000	186.411	14	0
2906_C1_PL5019	86999	10000	183.444	41	0
2906_C1_PL5099	51704	10000	192.769	33	0.001
2906_C1_PL5157	220430	10000	187.977	6	0
2906_C1_PL7689	59873	10000	188.383	41	0.001
2906_C1_PL7946	34263	10000	188.967	43	0.001
2906_C1_PL7947	90837	10000	167.123	16	0
2906_C1_PL8128	131192	10000	162.462	21	0

SM Table 5 Page 9 Residual Primers

2906_C1_PL8172	333390	10000	191.528	10	0
2906_C2_KT5034	65688	10000	181.338	44	0.001
2906_C2_KT5786	63626	10000	187.426	22	0
2906_C2_MK0364	40165	10000	184.989	42	0.001
2906_C2_MK0365	81653	10000	171.956	15	0
2906_C2_MK0366	64350	10000	198.162	33	0.001
2906_C2_MK0368	102596	10000	178.44	22	0
3004_C1_KT5734	246053	10000	191.441	10	0
3004_C1_KT5738	324360	10000	190.494	27	0
3004_C1_KT5787	396633	10000	201.652	20	0
3004_C1_KT5788	299958	10000	198.774	27	0
3004_C1_KT5789	90155	10000	195.234	29	0
3004_C1_KT5832	105976	10000	193.786	32	0
3004_C1_KT5858	125483	10000	191.85	33	0
3004_C1_KT5859	279090	10000	171.038	3	0
3004_C1_KT5974	292338	10000	197.094	1	0
3004_C2_KT5214	203664	10000	201.927	23	0
3004_C2_KT5224	206475	10000	189.142	16	0
3004_C2_KT5225	284964	10000	189.025	29	0
3004_C2_KT5249	314432	10000	187.016	16	0
3004_C2_KT5317	280475	10000	192.747	17	0
3004_C2_KT5320	173431	10000	188.81	20	0
3004_C2_KT5382	131310	10000	191.24	30	0
3004_C2_KT5388	226424	10000	190.015	25	0
3004_C2_KT5486	256912	10000	192.705	25	0
3004_C2_KT5542	421720	10000	199.853	15	0
3007_C1_12578_EMLH	214299	10000	208.041	26	0
3007_C1_12579_EMLH	126826	10000	204.694	26	0
3007_C1_12580_EMLH	55608	10000	199.535	21	0
3007_C1_13247_EMLH	195284	10000	202.067	12	0
3007_C1_442_EMLH	49358	10000	206.302	16	0
3007_C1_443_EMLH	114621	10000	208.018	34	0
3007_C1_448_EMLH	57523	10000	205.661	34	0.001
3007_C1_75708_EMLH	31877	10000	202.166	27	0.001
3007_C1_77524_EMLK	32271	10000	196.028	32	0.001
3007_C1_77525_EMLK	480060	10000	192.124	14	0
3007_C1_77526_EMLK	191868	10000	182.562	8	0
3007_C1_77529_EMLK	213668	10000	184.543	9	0
3007_C1_77549_EMLK	13079	10000	200.447	37	0.003
3007_C1_77551_EMLK	45037	10000	196.941	36	0.001
3007_C1_77558_EMLK	66918	10000	197.239	26	0
3007_C1_77563_EMLK	114798	10000	195.497	23	0
3007_C1_77572_EMLK	38845	10000	191.18	35	0.001
3007_C1_KT7095	57647	10000	204.513	22	0
3007_C1_MK9396	225608	10000	194.986	28	0
3007_C2_77573_EMLK	262355	10000	203.94	6	0
3007_C2_77574_EMLK	45452	10000	195.98	36	0.001
3007_C2_77577_EMLK	12640	10000	198.453	33	0.003
3007_C2_77578_EMLK	28853	10000	192.535	27	0.001
3007_C2_77580_EMLK	23794	10000	187.767	18	0.001
3007_C2_77581_EMLK	46384	10000	188.159	18	0
3007_C2_77582_EMLK	356109	10000	196.32	4	0
3007_C2_77585_EMLK	25666	10000	192.287	17	0.001
3007_C2_77588_EMLK	38145	10000	197.588	21	0.001
3007_C2_77589_EMLK	111964	10000	193.809	12	0
3007_C2_77591_EMLK	83638	10000	193.05	26	0
3007_C2_77594_EMLK	274815	10000	187.587	7	0
3007_C2_77601_EMLK	61587	10000	195.345	18	0
3007_C2_77612_EMLK	135637	10000	195.226	12	0
3007_C2_77613_EMLK	60046	10000	200.256	42	0.001
3007_C2_77615_EMLK	78394	10000	201.533	28	0
3007_C2_77630_EMLK	27167	10000	197.848	33	0.001
3007_C2_77632_EMLK	29913	10000	200.165	32	0.001
3007_C2_77643_EMLK	50762	10000	168.558	27	0.001
3007_C2_77644_EMLK	73022	10000	170.828	12	0
3007_C2_77648_EMLK	54662	10000	191.01	27	0
3007_C2_77649_EMLK	103542	10000	176.834	24	0

SM_Table 6 Page 1 Minor Variant Fractions

reads_file	position	motif_A	snpA	snpA_count	motif_B	snpB	snpB_count	fraction_minor	total_count
0208_C1_77667_EMLK_125.spltChi.qc.fq	8072	TCGCTTTATTCTGTATATGCA	C	1527	TCGCTTTATTTTGTATATGCA	T	1	0.00065	1528
0208_C1_77667_EMLK_125.spltChi.qc.fq	10511	TGGAAAGTTTATTGGGCTGGT	A	1453	TGGAAAGTTTGTTGGGCTGGT	G	0	0.00000	1453
0208_C1_77667_EMLK_125.spltChi.qc.fq	18542	CGCACTCTTTATAATTAAGCT	A	1366	CGCACTCTTTGTAATTAAGCT	G	1	0.00073	1367
0208_C1_77667_EMLK_125.spltChi.qc.fq	18139	GGTTCTATCATATTAGAGATT	T	1331	GGTTCTATCACATTAGAGATT	C	1	0.00075	1332
0208_C1_77667_EMLK_125.spltChi.qc.fq	13404	AAGCATTTCCTAGCAATATGA	T	815	AAGCATTTCCCAGCAATATGA	C	1	0.00123	816
0208_C1_77667_EMLK_125.spltChi.qc.fq	12671	GGCTCACAAGATCCGTGAATT	A	801	GGCTCACAAGGTCCGTGAATT	G	0	0.00000	801
0208_C1_77667_EMLK_125.spltChi.qc.fq	2374	TTCAAGAGGCCAGGAACCAAG	C	739	TTCAAGAGGTCAGGAACCAAG	T	2	0.00271	741
0208_C1_77667_EMLK_125.spltChi.qc.fq	4393	AACCCAAACCAAAAATGATGA	A	695	AACCCAAACCTAAAATGATGA	T	0	0.00000	695
0208_C1_77667_EMLK_125.spltChi.qc.fq	5046	CACTCAACCACTGCCTGCTGC	C	644	CACTCAACCAATGCCTGCTGC	A	1	0.00155	645
0208_C1_77667_EMLK_125.spltChi.qc.fq	2776	AAGAATTTTTGATGTCTAAGG	G	572	AAGAATTTTTTATGTCTAAGG	T	0	0.00000	572
0208_C1_77667_EMLK_125.spltChi.qc.fq	3650	AACCACCACCTGGACCATCAC	T	490	AACCACCACCCGGACCATCAC	C	1	0.00204	491
0208_C1_77667_EMLK_125.spltChi.qc.fq	5556	AATCCGTCTACTAGAAGAGAT	A	431	AATCCGTCTACTAGAAGGGAT	G	0	0.00000	431
0208_C1_77667_EMLK_125.spltChi.qc.fq	18381	TTGATCTGATTTCAATAAATA	T	286	TTGATCTGATCTCAATAAATA	C	0	0.00000	286
0208_C1_77667_EMLK_125.spltChi.qc.fq	15285	TTCCGCAGCCTTAAGAGAGGC	T	242	TTCCGCAGCCCTAAGAGAGGC	C	0	0.00000	242
0208_C1_77667_EMLK_125.spltChi.qc.fq	5578	TTGATCAATATACTAAAGGTG	T	239	TTGATCAATACACTAAAGGTG	C	0	0.00000	239
0208_C1_77667_EMLK_125.spltChi.qc.fq	5524	CTCAGGGATAGTGATAACATC	T	227	CTCAGGGATAGTGACAACATC	C	0	0.00000	227
0208_C1_77667_EMLK_125.spltChi.qc.fq	13611	ATGGTGTTAAGAATGTTTTTA	G	200	ATGGTGTTAAAAATGTTTTTA	A	0	0.00000	200
0208_C1_77669_EMLK_125.spltChi.qc.fq	18139	GGTTCTATCATATTAGAGATT	T	653	GGTTCTATCACATTAGAGATT	C	1	0.00153	654
0208_C1_77669_EMLK_125.spltChi.qc.fq	13404	AAGCATTTCCTAGCAATATGA	T	585	AAGCATTTCCCAGCAATATGA	C	0	0.00000	585
0208_C1_77669_EMLK_125.spltChi.qc.fq	18542	CGCACTCTTTATAATTAAGCT	A	578	CGCACTCTTTGTAATTAAGCT	G	0	0.00000	578
0208_C1_77669_EMLK_125.spltChi.qc.fq	8072	TCGCTTTATTCTGTATATGCA	C	520	TCGCTTTATTTTGTATATGCA	T	0	0.00000	520
0208_C1_77669_EMLK_125.spltChi.qc.fq	10511	TGGAAAGTTTATTGGGCTGGT	A	504	TGGAAAGTTTGTTGGGCTGGT	G	1	0.00198	505
0208_C1_77669_EMLK_125.spltChi.qc.fq	4393	AACCCAAACCAAAAATGATGA	A	488	AACCCAAACCTAAAATGATGA	T	0	0.00000	488
0208_C1_77669_EMLK_125.spltChi.qc.fq	5046	CACTCAACCACTGCCTGCTGC	C	376	CACTCAACCAATGCCTGCTGC	A	0	0.00000	376
0208_C1_77669_EMLK_125.spltChi.qc.fq	12671	GGCTCACAAGATCCGTGAATT	A	362	GGCTCACAAGGTCCGTGAATT	G	1	0.00276	363
0208_C1_77669_EMLK_125.spltChi.qc.fq	2374	TTCAAGAGGCCAGGAACCAAG	C	283	TTCAAGAGGTCAGGAACCAAG	T	0	0.00000	283
0208_C1_77669_EMLK_125.spltChi.qc.fq	3650	AACCACCACCTGGACCATCAC	T	262	AACCACCACCCGGACCATCAC	C	0	0.00000	262
0208_C1_77669_EMLK_125.spltChi.qc.fq	5556	AATCCGTCTACTAGAAGAGAT	A	257	AATCCGTCTACTAGAAGGGAT	G	0	0.00000	257
0208_C1_77669_EMLK_125.spltChi.qc.fq	2776	AAGAATTTTTGATGTCTAAGG	G	256	AAGAATTTTTTATGTCTAAGG	T	0	0.00000	256
0208_C1_77669_EMLK_125.spltChi.qc.fq	15285	TTCCGCAGCCTTAAGAGAGGC	T	233	TTCCGCAGCCCTAAGAGAGGC	C	0	0.00000	233
0208_C1_77669_EMLK_125.spltChi.qc.fq	5578	TTGATCAATATACTAAAGGTG	T	199	TTGATCAATACACTAAAGGTG	C	0	0.00000	199
0208_C1_77669_EMLK_125.spltChi.qc.fq	18381	TTGATCTGATTTCAATAAATA	T	186	TTGATCTGATCTCAATAAATA	C	0	0.00000	186
0208_C1_77669_EMLK_125.spltChi.qc.fq	13611	ATGGTGTTAAGAATGTTTTTA	G	142	ATGGTGTTAAAAATGTTTTTA	A	3	0.02113	145
0208_C1_77670_EMLK_125.spltChi.qc.fq	18542	CGCACTCTTTATAATTAAGCT	A	1284	CGCACTCTTTGTAATTAAGCT	G	1	0.00078	1285
0208_C1_77670_EMLK_125.spltChi.qc.fq	18139	GGTTCTATCATATTAGAGATT	T	1240	GGTTCTATCACATTAGAGATT	C	1	0.00081	1241
0208_C1_77670_EMLK_125.spltChi.qc.fq	13404	AAGCATTTCCTAGCAATATGA	T	1235	AAGCATTTCCCAGCAATATGA	C	1	0.00081	1236
0208_C1_77670_EMLK_125.spltChi.qc.fq	8072	TCGCTTTATTCTGTATATGCA	C	1180	TCGCTTTATTTTGTATATGCA	T	0	0.00000	1180
0208_C1_77670_EMLK_125.spltChi.qc.fq	10511	TGGAAAGTTTATTGGGCTGGT	A	1052	TGGAAAGTTTGTTGGGCTGGT	G	1	0.00095	1053
0208_C1_77670_EMLK_125.spltChi.qc.fq	5046	CACTCAACCACTGCCTGCTGC	C	839	CACTCAACCAATGCCTGCTGC	A	0	0.00000	839
0208_C1_77670_EMLK_125.spltChi.qc.fq	4393	AACCCAAACCAAAAATGATGA	A	743	AACCCAAACCTAAAATGATGA	T	0	0.00000	743
0208_C1_77670_EMLK_125.spltChi.qc.fq	12671	GGCTCACAAGATCCGTGAATT	A	685	GGCTCACAAGGTCCGTGAATT	G	1	0.00146	686
0208_C1_77670_EMLK_125.spltChi.qc.fq	5556	AATCCGTCTACTAGAAGAGAT	A	647	AATCCGTCTACTAGAAGGGAT	G	1	0.00155	648
0208_C1_77670_EMLK_125.spltChi.qc.fq	2776	AAGAATTTTTGATGTCTAAGG	G	623	AAGAATTTTTTATGTCTAAGG	T	0	0.00000	623
0208_C1_77670_EMLK_125.spltChi.qc.fq	2374	TTCAAGAGGCCAGGAACCAAG	C	539	TTCAAGAGGTCAGGAACCAAG	T	0	0.00000	539
0208_C1_77670_EMLK_125.spltChi.qc.fq	3650	AACCACCACCTGGACCATCAC	T	511	AACCACCACCCGGACCATCAC	C	0	0.00000	511
0208_C1_77670_EMLK_125.spltChi.qc.fq	5578	TTGATCAATATACTAAAGGTG	T	498	TTGATCAATACACTAAAGGTG	C	0	0.00000	498
0208_C1_77670_EMLK_125.spltChi.qc.fq	15285	TTCCGCAGCCTTAAGAGAGGC	T	392	TTCCGCAGCCCTAAGAGAGGC	C	1	0.00255	393
0208_C1_77670_EMLK_125.spltChi.qc.fq	18381	TTGATCTGATTTCAATAAATA	T	322	TTGATCTGATCTCAATAAATA	C	0	0.00000	322
0208_C1_77670_EMLK_125.spltChi.qc.fq	13611	ATGGTGTTAAGAATGTTTTTA	G	281	ATGGTGTTAAAAATGTTTTTA	A	0	0.00000	281
0208_C1_77670_EMLK_125.spltChi.qc.fq	5524	CTCAGGGATAGTGATAACATC	T	204	CTCAGGGATAGTGACAACATC	C	0	0.00000	204
0208_C1_77673_EMLK_125.spltChi.qc.fq	18542	CGCACTCTTTATAATTAAGCT	A	990	CGCACTCTTTGTAATTAAGCT	G	3	0.00303	993
0208_C1_77673_EMLK_125.spltChi.qc.fq	8072	TCGCTTTATTCTGTATATGCA	C	908	TCGCTTTATTTTGTATATGCA	T	0	0.00000	908
0208_C1_77673_EMLK_125.spltChi.qc.fq	13404	AAGCATTTCCTAGCAATATGA	T	901	AAGCATTTCCCAGCAATATGA	C	2	0.00222	903
0208_C1_77673_EMLK_125.spltChi.qc.fq	18139	GGTTCTATCATATTAGAGATT	T	866	GGTTCTATCACATTAGAGATT	C	0	0.00000	866
0208_C1_77673_EMLK_125.spltChi.qc.fq	10511	TGGAAAGTTTATTGGGCTGGT	A	788	TGGAAAGTTTGTTGGGCTGGT	G	2	0.00254	790
0208_C1_77673_EMLK_125.spltChi.qc.fq	5046	CACTCAACCACTGCCTGCTGC	C	659	CACTCAACCAATGCCTGCTGC	A	0	0.00000	659
0208_C1_77673_EMLK_125.spltChi.qc.fq	4393	AACCCAAACCAAAAATGATGA	A	608	AACCCAAACCTAAAATGATGA	T	0	0.00000	608
0208_C1_77673_EMLK_125.spltChi.qc.fq	12671	GGCTCACAAGATCCGTGAATT	A	599	GGCTCACAAGGTCCGTGAATT	G	0	0.00000	599
0208_C1_77673_EMLK_125.spltChi.qc.fq	5556	AATCCGTCTACTAGAAGAGAT	A	471	AATCCGTCTACTAGAAGGGAT	G	1	0.00212	472
0208_C1_77673_EMLK_125.spltChi.qc.fq	3650	AACCACCACCTGGACCATCAC	T	382	AACCACCACCCGGACCATCAC	C	1	0.00262	383
0208_C1_77673_EMLK_125.spltChi.qc.fq	5578	TTGATCAATATACTAAAGGTG	T	374	TTGATCAATACACTAAAGGTG	C	1	0.00267	375
0208_C1_77673_EMLK_125.spltChi.qc.fq	2374	TTCAAGAGGCCAGGAACCAAG	C	367	TTCAAGAGGTCAGGAACCAAG	T	1	0.00272	368
0208_C1_77673_EMLK_125.spltChi.qc.fq	15285	TTCCGCAGCCTTAAGAGAGGC	T	356	TTCCGCAGCCCTAAGAGAGGC	C	2	0.00562	358
0208_C1_77673_EMLK_125.spltChi.qc.fq	2776	AAGAATTTTTGATGTCTAAGG	G	313	AAGAATTTTTTATGTCTAAGG	T	0	0.00000	313
0208_C1_77673_EMLK_125.spltChi.qc.fq	13611	ATGGTGTTAAGAATGTTTTTA	G	209	ATGGTGTTAAAAATGTTTTTA	A	1	0.00478	210
0208_C1_77673_EMLK_125.spltChi.qc.fq	18381	TTGATCTGATTTCAATAAATA	T	173	TTGATCTGATCTCAATAAATA	C	0	0.00000	173
0208_C1_77673_EMLK_125.spltChi.qc.fq	5524	CTCAGGGATAGTGATAACATC	T	138	CTCAGGGATAGTGACAACATC	C	0	0.00000	138
0208_C1_77675_EMLK_125.spltChi.qc.fq	18139	GGTTCTATCATATTAGAGATT	T	807	GGTTCTATCACATTAGAGATT	C	1	0.00124	808
0208_C1_77675_EMLK_125.spltChi.qc.fq	10511	TGGAAAGTTTATTGGGCTGGT	A	776	TGGAAAGTTTGTTGGGCTGGT	G	1	0.00129	777
0208_C1_77675_EMLK_125.spltChi.qc.fq	13404	AAGCATTTCCTAGCAATATGA	T	746	AAGCATTTCCCAGCAATATGA	C	2	0.00268	748
0208_C1_77675_EMLK_125.spltChi.qc.fq	18542	CGCACTCTTTATAATTAAGCT	A	676	CGCACTCTTTGTAATTAAGCT	G	1	0.00148	677
0208_C1_77675_EMLK_125.spltChi.qc.fq	8072	TCGCTTTATTCTGTATATGCA	C	610	TCGCTTTATTTTGTATATGCA	T	1	0.00164	611
0208_C1_77675_EMLK_125.spltChi.qc.fq	12671	GGCTCACAAGATCCGTGAATT	A	512	GGCTCACAAGGTCCGTGAATT	G	0	0.00000	512
0208_C1_77675_EMLK_125.spltChi.qc.fq	5046	CACTCAACCACTGCCTGCTGC	C	458	CACTCAACCAATGCCTGCTGC	A	0	0.00000	458
0208_C1_77675_EMLK_125.spltChi.qc.fq	2374	TTCAAGAGGCCAGGAACCAAG	C	371	TTCAAGAGGTCAGGAACCAAG	T	1	0.00270	372
0208_C1_77675_EMLK_125.spltChi.qc.fq	2776	AAGAATTTTTGATGTCTAAGG	G	364	AAGAATTTTTTATGTCTAAGG	T	0	0.00000	364
0208_C1_77675_EMLK_125.spltChi.qc.fq	5556	AATCCGTCTACTAGAAGAGAT	A	312	AATCCGTCTACTAGAAGGGAT	G	1	0.00321	313
0208_C1_77675_EMLK_125.spltChi.qc.fq	3650	AACCACCACCTGGACCATCAC	T	300	AACCACCACCCGGACCATCAC	C	1	0.00333	301
0208_C1_77675_EMLK_125.spltChi.qc.fq	15285	TTCCGCAGCCTTAAGAGAGGC	T	277	TTCCGCAGCCCTAAGAGAGGC	C	0	0.00000	277
0208_C1_77675_EMLK_125.spltChi.qc.fq	18381	TTGATCTGATTTCAATAAATA	T	217	TTGATCTGATCTCAATAAATA	C	0	0.00000	217
0208_C1_77675_EMLK_125.spltChi.qc.fq	5578	TTGATCAATATACTAAAGGTG	T	209	TTGATCAATACACTAAAGGTG	C	0	0.00000	209
0208_C1_77675_EMLK_125.spltChi.qc.fq	4393	AACCCAAACCAAAAATGATGA	A	203	AACCCAAACCTAAAATGATGA	T	0	0.00000	203
0208_C1_77675_EMLK_125.spltChi.qc.fq	13611	ATGGTGTTAAGAATGTTTTTA	G	171	ATGGTGTTAAAAATGTTTTTA	A	0	0.00000	171
0208_C1_77675_EMLK_125.spltChi.qc.fq	5524	CTCAGGGATAGTGATAACATC	T	132	CTCAGGGATAGTGACAACATC	C	0	0.00000	132
0208_C1_77676_EMLK_125.spltChi.qc.fq	8072	TCGCTTTATTCTGTATATGCA	C	4966	TCGCTTTATTTTGTATATGCA	T	2	0.00040	4968
0208_C1_77676_EMLK_125.spltChi.qc.fq	18542	CGCACTCTTTATAATTAAGCT	A	3987	CGCACTCTTTGTAATTAAGCT	G	1	0.00025	3988
0208_C1_77676_EMLK_125.spltChi.qc.fq	10511	TGGAAAGTTTATTGGGCTGGT	A	3576	TGGAAAGTTTGTTGGGCTGGT	G	1	0.00028	3577
0208_C1_77676_EMLK_125.spltChi.qc.fq	18139	GGTTCTATCATATTAGAGATT	T	2823	GGTTCTATCACATTAGAGATT	C	1	0.00035	2824
0208_C1_77676_EMLK_125.spltChi.qc.fq	5046	CACTCAACCACTGCCTGCTGC	C	2574	CACTCAACCAATGCCTGCTGC	A	0	0.00000	2574
0208_C1_77676_EMLK_125.spltChi.qc.fq	2374	TTCAAGAGGCCAGGAACCAAG	C	2233	TTCAAGAGGTCAGGAACCAAG	T	5	0.00224	2238
0208_C1_77676_EMLK_125.spltChi.qc.fq	13404	AAGCATTTCCTAGCAATATGA	T	2237	AAGCATTTCCCAGCAATATGA	C	1	0.00045	2238

SM_Table 6 Page 2 Minor Variant Fractions

0208_C1_77676_EMLK_125.spltChi.qc.fq	5556	AATCCGTCTACTAGAAGAGAT	A	2041	AATCCGTCTACTAGAAGGGAT	G	1	0.00049	2042
0208_C1_77676_EMLK_125.spltChi.qc.fq	3650	AACCACCACCTGGACCATCAC	T	1959	AACCACCACCCGGACCATCAC	C	4	0.00204	1963
0208_C1_77676_EMLK_125.spltChi.qc.fq	12671	GGCTCACAAGATCCGTGAATT	A	1910	GGCTCACAAGGTCCGTGAATT	G	2	0.00105	1912
0208_C1_77676_EMLK_125.spltChi.qc.fq	4393	AACCCAAACCAAAAATGATGA	A	1547	AACCCAAACCTAAAATGATGA	T	2	0.00129	1549
0208_C1_77676_EMLK_125.spltChi.qc.fq	15285	TTCCGCAGCCTTAAGAGAGGC	T	1478	TTCCGCAGCCCTAAGAGAGGC	C	2	0.00135	1480
0208_C1_77676_EMLK_125.spltChi.qc.fq	5578	TTGATCAATATACTAAAGGTG	T	1313	TTGATCAATACACTAAAGGTG	C	1	0.00076	1314
0208_C1_77676_EMLK_125.spltChi.qc.fq	13611	ATGGTGTTAAGAATGTTTTTA	G	1284	ATGGTGTTAAAAATGTTTTTA	A	1	0.00078	1285
0208_C1_77676_EMLK_125.spltChi.qc.fq	5524	CTCAGGGATAGTGATAACATC	T	1011	CTCAGGGATAGTGACAACATC	C	0	0.00000	1011
0208_C1_77676_EMLK_125.spltChi.qc.fq	18381	TTGATCTGATTTCAATAAATA	T	654	TTGATCTGATCTCAATAAATA	C	1	0.00153	655
0208_C1_77676_EMLK_125.spltChi.qc.fq	2776	AAGAATTTTTGATGTCTAAGG	G	515	AAGAATTTTTTATGTCTAAGG	T	0	0.00000	515
0208_C1_77676_EMLK_125.spltChi.qc.fq	10476	TCTGTAACTTCTTAGTTAGTC	C	155	TCTGTAACTTTTTAGTTAGTC	T	0	0.00000	155
0208_C1_77677_EMLK_125.spltChi.qc.fq	18542	CGCACTCTTTATAATTAAGCT	A	2241	CGCACTCTTTGTAATTAAGCT	G	1	0.00045	2242
0208_C1_77677_EMLK_125.spltChi.qc.fq	8072	TCGCTTTATTCTGTATATGCA	C	1632	TCGCTTTATTTTGTATATGCA	T	0	0.00000	1632
0208_C1_77677_EMLK_125.spltChi.qc.fq	10511	TGGAAAGTTTATTGGGCTGGT	A	1544	TGGAAAGTTTGTTGGGCTGGT	G	1	0.00065	1545
0208_C1_77677_EMLK_125.spltChi.qc.fq	13404	AAGCATTTCCTAGCAATATGA	T	1002	AAGCATTTCCCAGCAATATGA	C	0	0.00000	1002
0208_C1_77677_EMLK_125.spltChi.qc.fq	18139	GGTTCTATCATATTAGAGATT	T	933	GGTTCTATCACATTAGAGATT	C	1	0.00107	934
0208_C1_77677_EMLK_125.spltChi.qc.fq	5046	CACTCAACCACTGCCTGCTGC	C	900	CACTCAACCAATGCCTGCTGC	A	0	0.00000	900
0208_C1_77677_EMLK_125.spltChi.qc.fq	12671	GGCTCACAAGATCCGTGAATT	A	779	GGCTCACAAGGTCCGTGAATT	G	1	0.00128	780
0208_C1_77677_EMLK_125.spltChi.qc.fq	2374	TTCAAGAGGCCAGGAACCAAG	C	705	TTCAAGAGGTCAGGAACCAAG	T	2	0.00284	707
0208_C1_77677_EMLK_125.spltChi.qc.fq	3650	AACCACCACCTGGACCATCAC	T	646	AACCACCACCCGGACCATCAC	C	0	0.00000	646
0208_C1_77677_EMLK_125.spltChi.qc.fq	5556	AATCCGTCTACTAGAAGAGAT	A	602	AATCCGTCTACTAGAAGGGAT	G	1	0.00166	603
0208_C1_77677_EMLK_125.spltChi.qc.fq	4393	AACCCAAACCAAAAATGATGA	A	533	AACCCAAACCTAAAATGATGA	T	1	0.00188	534
0208_C1_77677_EMLK_125.spltChi.qc.fq	15285	TTCCGCAGCCTTAAGAGAGGC	T	483	TTCCGCAGCCCTAAGAGAGGC	C	0	0.00000	483
0208_C1_77677_EMLK_125.spltChi.qc.fq	5578	TTGATCAATATACTAAAGGTG	T	439	TTGATCAATACACTAAAGGTG	C	1	0.00228	440
0208_C1_77677_EMLK_125.spltChi.qc.fq	13611	ATGGTGTTAAGAATGTTTTTA	G	359	ATGGTGTTAAAAATGTTTTTA	A	0	0.00000	359
0208_C1_77677_EMLK_125.spltChi.qc.fq	5524	CTCAGGGATAGTGATAACATC	T	224	CTCAGGGATAGTGACAACATC	C	1	0.00446	225
0208_C1_77677_EMLK_125.spltChi.qc.fq	2776	AAGAATTTTTGATGTCTAAGG	G	218	AAGAATTTTTTATGTCTAAGG	T	0	0.00000	218
0208_C1_77677_EMLK_125.spltChi.qc.fq	18381	TTGATCTGATTTCAATAAATA	T	203	TTGATCTGATCTCAATAAATA	C	0	0.00000	203
0208_C1_77679_EMLK_125.spltChi.qc.fq	18139	GGTTCTATCATATTAGAGATT	T	896	GGTTCTATCACATTAGAGATT	C	1	0.00112	897
0208_C1_77679_EMLK_125.spltChi.qc.fq	8072	TCGCTTTATTCTGTATATGCA	C	861	TCGCTTTATTTTGTATATGCA	T	1	0.00116	862
0208_C1_77679_EMLK_125.spltChi.qc.fq	18542	CGCACTCTTTATAATTAAGCT	A	788	CGCACTCTTTGTAATTAAGCT	G	3	0.00381	791
0208_C1_77679_EMLK_125.spltChi.qc.fq	10511	TGGAAAGTTTATTGGGCTGGT	A	777	TGGAAAGTTTGTTGGGCTGGT	G	0	0.00000	777
0208_C1_77679_EMLK_125.spltChi.qc.fq	13404	AAGCATTTCCTAGCAATATGA	T	748	AAGCATTTCCCAGCAATATGA	C	1	0.00134	749
0208_C1_77679_EMLK_125.spltChi.qc.fq	4393	AACCCAAACCAAAAATGATGA	A	590	AACCCAAACCTAAAATGATGA	T	0	0.00000	590
0208_C1_77679_EMLK_125.spltChi.qc.fq	12671	GGCTCACAAGATCCGTGAATT	A	544	GGCTCACAAGGTCCGTGAATT	G	0	0.00000	544
0208_C1_77679_EMLK_125.spltChi.qc.fq	5046	CACTCAACCACTGCCTGCTGC	C	524	CACTCAACCAATGCCTGCTGC	A	0	0.00000	524
0208_C1_77679_EMLK_125.spltChi.qc.fq	5556	AATCCGTCTACTAGAAGAGAT	A	406	AATCCGTCTACTAGAAGGGAT	G	0	0.00000	406
0208_C1_77679_EMLK_125.spltChi.qc.fq	2374	TTCAAGAGGCCAGGAACCAAG	C	369	TTCAAGAGGTCAGGAACCAAG	T	0	0.00000	369
0208_C1_77679_EMLK_125.spltChi.qc.fq	2776	AAGAATTTTTGATGTCTAAGG	G	361	AAGAATTTTTTATGTCTAAGG	T	0	0.00000	361
0208_C1_77679_EMLK_125.spltChi.qc.fq	3650	AACCACCACCTGGACCATCAC	T	357	AACCACCACCCGGACCATCAC	C	0	0.00000	357
0208_C1_77679_EMLK_125.spltChi.qc.fq	5578	TTGATCAATATACTAAAGGTG	T	295	TTGATCAATACACTAAAGGTG	C	0	0.00000	295
0208_C1_77679_EMLK_125.spltChi.qc.fq	15285	TTCCGCAGCCTTAAGAGAGGC	T	285	TTCCGCAGCCCTAAGAGAGGC	C	0	0.00000	285
0208_C1_77679_EMLK_125.spltChi.qc.fq	13611	ATGGTGTTAAGAATGTTTTTA	G	236	ATGGTGTTAAAAATGTTTTTA	A	0	0.00000	236
0208_C1_77679_EMLK_125.spltChi.qc.fq	18381	TTGATCTGATTTCAATAAATA	T	232	TTGATCTGATCTCAATAAATA	C	0	0.00000	232
0208_C1_77679_EMLK_125.spltChi.qc.fq	5524	CTCAGGGATAGTGATAACATC	T	164	CTCAGGGATAGTGACAACATC	C	0	0.00000	164
0208_C1_77681_EMLK_125.spltChi.qc.fq	18542	CGCACTCTTTATAATTAAGCT	A	3194	CGCACTCTTTGTAATTAAGCT	G	1	0.00031	3195
0208_C1_77681_EMLK_125.spltChi.qc.fq	8072	TCGCTTTATTCTGTATATGCA	C	2647	TCGCTTTATTTTGTATATGCA	T	1	0.00038	2648
0208_C1_77681_EMLK_125.spltChi.qc.fq	10511	TGGAAAGTTTATTGGGCTGGT	A	1829	TGGAAAGTTTGTTGGGCTGGT	G	0	0.00000	1829
0208_C1_77681_EMLK_125.spltChi.qc.fq	18139	GGTTCTATCATATTAGAGATT	T	1358	GGTTCTATCACATTAGAGATT	C	1	0.00074	1359
0208_C1_77681_EMLK_125.spltChi.qc.fq	5046	CACTCAACCACTGCCTGCTGC	C	1309	CACTCAACCAATGCCTGCTGC	A	0	0.00000	1309
0208_C1_77681_EMLK_125.spltChi.qc.fq	13404	AAGCATTTCCTAGCAATATGA	T	1296	AAGCATTTCCCAGCAATATGA	C	1	0.00077	1297
0208_C1_77681_EMLK_125.spltChi.qc.fq	12671	GGCTCACAAGATCCGTGAATT	A	992	GGCTCACAAGGTCCGTGAATT	G	2	0.00202	994
0208_C1_77681_EMLK_125.spltChi.qc.fq	3650	AACCACCACCTGGACCATCAC	T	873	AACCACCACCCGGACCATCAC	C	2	0.00229	875
0208_C1_77681_EMLK_125.spltChi.qc.fq	5556	AATCCGTCTACTAGAAGAGAT	A	863	AATCCGTCTACTAGAAGGGAT	G	0	0.00000	863
0208_C1_77681_EMLK_125.spltChi.qc.fq	2374	TTCAAGAGGCCAGGAACCAAG	C	855	TTCAAGAGGTCAGGAACCAAG	T	2	0.00234	857
0208_C1_77681_EMLK_125.spltChi.qc.fq	15285	TTCCGCAGCCTTAAGAGAGGC	T	764	TTCCGCAGCCCTAAGAGAGGC	C	0	0.00000	764
0208_C1_77681_EMLK_125.spltChi.qc.fq	5578	TTGATCAATATACTAAAGGTG	T	672	TTGATCAATACACTAAAGGTG	C	0	0.00000	672
0208_C1_77681_EMLK_125.spltChi.qc.fq	13611	ATGGTGTTAAGAATGTTTTTA	G	659	ATGGTGTTAAAAATGTTTTTA	A	1	0.00152	660
0208_C1_77681_EMLK_125.spltChi.qc.fq	4393	AACCCAAACCAAAAATGATGA	A	404	AACCCAAACCTAAAATGATGA	T	0	0.00000	404
0208_C1_77681_EMLK_125.spltChi.qc.fq	5524	CTCAGGGATAGTGATAACATC	T	254	CTCAGGGATAGTGACAACATC	C	0	0.00000	254
0208_C1_MK10128R_125.spltChi.qc.fq	8072	TCGCTTTATTCTGTATATGCA	C	10	TCGCTTTATTTTGTATATGCA	T	8568	0.00117	8578
0208_C1_MK10128R_125.spltChi.qc.fq	18542	CGCACTCTTTATAATTAAGCT	A	6	CGCACTCTTTGTAATTAAGCT	G	6183	0.00097	6189
0208_C1_MK10128R_125.spltChi.qc.fq	18139	GGTTCTATCATATTAGAGATT	T	4	GGTTCTATCACATTAGAGATT	C	4433	0.00090	4437
0208_C1_MK10128R_125.spltChi.qc.fq	2374	TTCAAGAGGCCAGGAACCAAG	C	13	TTCAAGAGGTCAGGAACCAAG	T	3928	0.00331	3941
0208_C1_MK10128R_125.spltChi.qc.fq	10511	TGGAAAGTTTATTGGGCTGGT	A	2	TGGAAAGTTTGTTGGGCTGGT	G	3478	0.00058	3480
0208_C1_MK10128R_125.spltChi.qc.fq	5046	CACTCAACCACTGCCTGCTGC	C	3	CACTCAACCAATGCCTGCTGC	A	2804	0.00107	2807
0208_C1_MK10128R_125.spltChi.qc.fq	12671	GGCTCACAAGATCCGTGAATT	A	3	GGCTCACAAGGTCCGTGAATT	G	2442	0.00123	2445
0208_C1_MK10128R_125.spltChi.qc.fq	4393	AACCCAAACCAAAAATGATGA	A	1	AACCCAAACCTAAAATGATGA	T	2251	0.00044	2252
0208_C1_MK10128R_125.spltChi.qc.fq	3650	AACCACCACCTGGACCATCAC	T	10	AACCACCACCCGGACCATCAC	C	2011	0.00497	2021
0208_C1_MK10128R_125.spltChi.qc.fq	13404	AAGCATTTCCTAGCAATATGA	T	1	AAGCATTTCCCAGCAATATGA	C	1665	0.00060	1666
0208_C1_MK10128R_125.spltChi.qc.fq	15285	TTCCGCAGCCTTAAGAGAGGC	T	0	TTCCGCAGCCCTAAGAGAGGC	C	1510	0.00000	1510
0208_C1_MK10128R_125.spltChi.qc.fq	18381	TTGATCTGATTTCAATAAATA	T	1	TTGATCTGATCTCAATAAATA	C	1484	0.00067	1485
0208_C1_MK10128R_125.spltChi.qc.fq	13611	ATGGTGTTAAGAATGTTTTTA	G	1	ATGGTGTTAAAAATGTTTTTA	A	1408	0.00071	1409
0208_C1_MK10128R_125.spltChi.qc.fq	5556	AATCCGTCTACTAGAAGAGAT	A	2	AATCCGTCTACTAGAAGGGAT	G	811	0.00247	813
0208_C1_MK10128R_125.spltChi.qc.fq	2776	AAGAATTTTTGATGTCTAAGG	G	0	AAGAATTTTTTATGTCTAAGG	T	799	0.00000	799
0208_C1_MK10128R_125.spltChi.qc.fq	5578	TTGATCAATATACTAAAGGTG	T	0	TTGATCAATACACTAAAGGTG	C	762	0.00000	762
0208_C1_MK10128R_125.spltChi.qc.fq	10476	TCTGTAACTTCTTAGTTAGTC	C	0	TCTGTAACTTTTTAGTTAGTC	T	150	0.00000	150
0208_C1_MK10128R_125.spltChi.qc.fq	5524	CTCAGGGATAGTGATAACATC	T	0	CTCAGGGATAGTGACAACATC	C	105	0.00000	105
0208_C1_MK10173_125.spltChi.qc.fq	8072	TCGCTTTATTCTGTATATGCA	C	6	TCGCTTTATTTTGTATATGCA	T	4847	0.00124	4853
0208_C1_MK10173_125.spltChi.qc.fq	18542	CGCACTCTTTATAATTAAGCT	A	6	CGCACTCTTTGTAATTAAGCT	G	3760	0.00160	3766
0208_C1_MK10173_125.spltChi.qc.fq	10511	TGGAAAGTTTATTGGGCTGGT	A	3	TGGAAAGTTTGTTGGGCTGGT	G	2645	0.00113	2648
0208_C1_MK10173_125.spltChi.qc.fq	2374	TTCAAGAGGCCAGGAACCAAG	C	10	TTCAAGAGGTCAGGAACCAAG	T	2571	0.00389	2581
0208_C1_MK10173_125.spltChi.qc.fq	18139	GGTTCTATCATATTAGAGATT	T	3	GGTTCTATCACATTAGAGATT	C	2546	0.00118	2549
0208_C1_MK10173_125.spltChi.qc.fq	5046	CACTCAACCACTGCCTGCTGC	C	2	CACTCAACCAATGCCTGCTGC	A	1852	0.00108	1854
0208_C1_MK10173_125.spltChi.qc.fq	12671	GGCTCACAAGATCCGTGAATT	A	1	GGCTCACAAGGTCCGTGAATT	G	1794	0.00056	1795
0208_C1_MK10173_125.spltChi.qc.fq	13404	AAGCATTTCCTAGCAATATGA	T	3	AAGCATTTCCCAGCAATATGA	C	1604	0.00187	1607
0208_C1_MK10173_125.spltChi.qc.fq	3650	AACCACCACCTGGACCATCAC	T	4	AACCACCACCCGGACCATCAC	C	1485	0.00269	1489
0208_C1_MK10173_125.spltChi.qc.fq	4393	AACCCAAACCAAAAATGATGA	A	0	AACCCAAACCTAAAATGATGA	T	1277	0.00000	1277
0208_C1_MK10173_125.spltChi.qc.fq	15285	TTCCGCAGCCTTAAGAGAGGC	T	0	TTCCGCAGCCCTAAGAGAGGC	C	1122	0.00000	1122
0208_C1_MK10173_125.spltChi.qc.fq	13611	ATGGTGTTAAGAATGTTTTTA	G	0	ATGGTGTTAAAAATGTTTTTA	A	858	0.00000	858
0208_C1_MK10173_125.spltChi.qc.fq	18381	TTGATCTGATTTCAATAAATA	T	0	TTGATCTGATCTCAATAAATA	C	744	0.00000	744
0208_C1_MK10173_125.spltChi.qc.fq	5556	AATCCGTCTACTAGAAGAGAT	A	0	AATCCGTCTACTAGAAGGGAT	G	534	0.00000	534

SM_Table 6 Page 3 Minor Variant Fractions

0208_C1_MK10173_125.spltChi.qc.fq	5578	TTGATCAATATACTAAAGGTG	T	0	TTGATCAATACACTAAAGGTG	C	513	0.00000	513
0208_C1_MK10173_125.spltChi.qc.fq	2776	AAGAATTTTTTGATGTCTAAGG	G	0	AAGAATTTTTTATGTCTAAGG	T	245	0.00000	245
0208_C1_MK10173_125.spltChi.qc.fq	10476	TCTGTAACTTCTTAGTTAGTC	C	0	TCTGTAACTTTTTAGTTAGTC	T	163	0.00000	163
2208_C1_MK0433R2_125.spltChi.qc.fq	6029	AGCTTCACTAGAAGGATATTG	G	12	AGCTTCACTAAAAGGATATTG	A	112	0.10714	124
2208_C1_MK0434R2_125.spltChi.qc.fq	6029	AGCTTCACTAGAAGGATATTG	G	9	AGCTTCACTAAAAGGATATTG	A	202	0.04455	211
2208_C1_MK0434R2_125.spltChi.qc.fq	10136	ACATTGTATTAGGGGCAATAA	A	7	ACATTGTATTGGGGGCAATAA	G	156	0.04487	163
2208_C1_MK0549R2_125.spltChi.qc.fq	6029	AGCTTCACTAGAAGGATATTG	G	21	AGCTTCACTAAAAGGATATTG	A	81	0.25926	102
2208_C1_MK2789R3_125.spltChi.qc.fq	10136	ACATTGTATTAGGGGCAATAA	A	445	ACATTGTATTGGGGGCAATAA	G	2	0.00449	447
2208_C1_MK2789R3_125.spltChi.qc.fq	6029	AGCTTCACTAGAAGGATATTG	G	393	AGCTTCACTAAAAGGATATTG	A	10	0.02545	403
2208_C1_MK2789R3_125.spltChi.qc.fq	18301	ATAGTATTTTGATACTTGTAA	G	147	ATAGTATTTTTTATACTTGTAA	T	3	0.02041	150
2208_C1_MK2918R_125.spltChi.qc.fq	10136	ACATTGTATTAGGGGCAATAA	A	2447	ACATTGTATTGGGGGCAATAA	G	3	0.00123	2450
2208_C1_MK2918R_125.spltChi.qc.fq	6029	AGCTTCACTAGAAGGATATTG	G	1973	AGCTTCACTAAAAGGATATTG	A	1	0.00051	1974
2208_C1_MK2918R_125.spltChi.qc.fq	13663	ATATGCATGTCAGTGATTATT	C	1020	ATATGCATGTTAGTGATTATT	T	1	0.00098	1021
2208_C1_MK2918R_125.spltChi.qc.fq	18301	ATAGTATTTTGATACTTGTAA	G	895	ATAGTATTTTTTATACTTGTAA	T	0	0.00000	895
2208_C1_MK3183R2_125.spltChi.qc.fq	6029	AGCTTCACTAGAAGGATATTG	G	2531	AGCTTCACTAAAAGGATATTG	A	4	0.00158	2535
2208_C1_MK3183R2_125.spltChi.qc.fq	10136	ACATTGTATTAGGGGCAATAA	A	1438	ACATTGTATTGGGGGCAATAA	G	4	0.00278	1442
2208_C1_MK3183R2_125.spltChi.qc.fq	13663	ATATGCATGTCAGTGATTATT	C	764	ATATGCATGTTAGTGATTATT	T	1	0.00131	765
2208_C1_MK3183R2_125.spltChi.qc.fq	18301	ATAGTATTTTGATACTTGTAA	G	756	ATAGTATTTTTTATACTTGTAA	T	0	0.00000	756
2208_C1_PL4127R_125.spltChi.qc.fq	6029	AGCTTCACTAGAAGGATATTG	G	1114	AGCTTCACTAAAAGGATATTG	A	2	0.00180	1116
2208_C1_PL4127R_125.spltChi.qc.fq	10136	ACATTGTATTAGGGGCAATAA	A	825	ACATTGTATTGGGGGCAATAA	G	4	0.00485	829
2208_C1_PL4127R_125.spltChi.qc.fq	13663	ATATGCATGTCAGTGATTATT	C	322	ATATGCATGTTAGTGATTATT	T	1	0.00311	323
2208_C1_PL4127R_125.spltChi.qc.fq	18301	ATAGTATTTTGATACTTGTAA	G	284	ATAGTATTTTTTATACTTGTAA	T	0	0.00000	284
2208_C1_PL4169R_125.spltChi.qc.fq	6029	AGCTTCACTAGAAGGATATTG	G	827	AGCTTCACTAAAAGGATATTG	A	3	0.00363	830
2208_C1_PL4169R_125.spltChi.qc.fq	10136	ACATTGTATTAGGGGCAATAA	A	618	ACATTGTATTGGGGGCAATAA	G	1	0.00162	619
2208_C1_PL4169R_125.spltChi.qc.fq	13663	ATATGCATGTCAGTGATTATT	C	268	ATATGCATGTTAGTGATTATT	T	0	0.00000	268
2208_C1_PL4169R_125.spltChi.qc.fq	18301	ATAGTATTTTGATACTTGTAA	G	247	ATAGTATTTTTTATACTTGTAA	T	0	0.00000	247
2208_C1_PL4197R_125.spltChi.qc.fq	6029	AGCTTCACTAGAAGGATATTG	G	821	AGCTTCACTAAAAGGATATTG	A	1	0.00122	822
2208_C1_PL4197R_125.spltChi.qc.fq	10136	ACATTGTATTAGGGGCAATAA	A	639	ACATTGTATTGGGGGCAATAA	G	1	0.00156	640
2208_C1_PL4197R_125.spltChi.qc.fq	13663	ATATGCATGTCAGTGATTATT	C	385	ATATGCATGTTAGTGATTATT	T	0	0.00000	385
2208_C1_PL4197R_125.spltChi.qc.fq	18301	ATAGTATTTTGATACTTGTAA	G	367	ATAGTATTTTTTATACTTGTAA	T	0	0.00000	367
2208_C1_PL4203R_125.spltChi.qc.fq	10136	ACATTGTATTAGGGGCAATAA	A	575	ACATTGTATTGGGGGCAATAA	G	1	0.00174	576
2208_C1_PL4203R_125.spltChi.qc.fq	6029	AGCTTCACTAGAAGGATATTG	G	522	AGCTTCACTAAAAGGATATTG	A	4	0.00766	526
2208_C1_PL4203R_125.spltChi.qc.fq	18301	ATAGTATTTTGATACTTGTAA	G	206	ATAGTATTTTTTATACTTGTAA	T	0	0.00000	206
2208_C1_PL4203R_125.spltChi.qc.fq	13663	ATATGCATGTCAGTGATTATT	C	201	ATATGCATGTTAGTGATTATT	T	0	0.00000	201
2208_C1_PL4256R_125.spltChi.qc.fq	6029	AGCTTCACTAGAAGGATATTG	G	4898	AGCTTCACTAAAAGGATATTG	A	5	0.00102	4903
2208_C1_PL4256R_125.spltChi.qc.fq	10136	ACATTGTATTAGGGGCAATAA	A	2598	ACATTGTATTGGGGGCAATAA	G	2	0.00077	2600
2208_C1_PL4256R_125.spltChi.qc.fq	18301	ATAGTATTTTGATACTTGTAA	G	1137	ATAGTATTTTTTATACTTGTAA	T	0	0.00000	1137
2208_C1_PL4256R_125.spltChi.qc.fq	13663	ATATGCATGTCAGTGATTATT	C	638	ATATGCATGTTAGTGATTATT	T	0	0.00000	638
2208_C1_PL4284R_125.spltChi.qc.fq	6029	AGCTTCACTAGAAGGATATTG	G	522	AGCTTCACTAAAAGGATATTG	A	10	0.01916	532
2208_C1_PL4284R_125.spltChi.qc.fq	10136	ACATTGTATTAGGGGCAATAA	A	431	ACATTGTATTGGGGGCAATAA	G	1	0.00232	432
2208_C1_PL4284R_125.spltChi.qc.fq	13663	ATATGCATGTCAGTGATTATT	C	158	ATATGCATGTTAGTGATTATT	T	0	0.00000	158
2208_C1_PL4284R_125.spltChi.qc.fq	18301	ATAGTATTTTGATACTTGTAA	G	142	ATAGTATTTTTTATACTTGTAA	T	0	0.00000	142
2208_C1_PL4292R_125.spltChi.qc.fq	10136	ACATTGTATTAGGGGCAATAA	A	1589	ACATTGTATTGGGGGCAATAA	G	1	0.00063	1590
2208_C1_PL4292R_125.spltChi.qc.fq	6029	AGCTTCACTAGAAGGATATTG	G	1285	AGCTTCACTAAAAGGATATTG	A	0	0.00000	1285
2208_C1_PL4292R_125.spltChi.qc.fq	18301	ATAGTATTTTGATACTTGTAA	G	1165	ATAGTATTTTTTATACTTGTAA	T	0	0.00000	1165
2208_C1_PL4292R_125.spltChi.qc.fq	13663	ATATGCATGTCAGTGATTATT	C	1114	ATATGCATGTTAGTGATTATT	T	0	0.00000	1114
2208_C1_PL4432R_125.spltChi.qc.fq	6029	AGCTTCACTAGAAGGATATTG	G	461	AGCTTCACTAAAAGGATATTG	A	2	0.00434	463
2208_C1_PL4432R_125.spltChi.qc.fq	18301	ATAGTATTTTGATACTTGTAA	G	167	ATAGTATTTTTTATACTTGTAA	T	0	0.00000	167
2208_C1_PL4432R_125.spltChi.qc.fq	13663	ATATGCATGTCAGTGATTATT	C	160	ATATGCATGTTAGTGATTATT	T	0	0.00000	160
2208_C1_PL4736R_125.spltChi.qc.fq	6029	AGCTTCACTAGAAGGATATTG	G	667	AGCTTCACTAAAAGGATATTG	A	1	0.00150	668
2208_C1_PL4736R_125.spltChi.qc.fq	10136	ACATTGTATTAGGGGCAATAA	A	479	ACATTGTATTGGGGGCAATAA	G	0	0.00000	479
2208_C1_PL4736R_125.spltChi.qc.fq	13663	ATATGCATGTCAGTGATTATT	C	198	ATATGCATGTTAGTGATTATT	T	1	0.00505	199
2208_C1_PL4736R_125.spltChi.qc.fq	18301	ATAGTATTTTGATACTTGTAA	G	198	ATAGTATTTTTTATACTTGTAA	T	0	0.00000	198
2906_C2_KT5034_125.spltChi.qc.fq	6029	AGCTTCACTAGAAGGATATTG	G	494	AGCTTCACTAAAAGGATATTG	A	9	0.01822	503
2906_C2_KT5034_125.spltChi.qc.fq	5865	ATTTCTAAGTCGTTCACAATC	C	485	ATTTCTAAGTTGTTCACAATC	T	6	0.01237	491
2906_C2_KT5034_125.spltChi.qc.fq	11455	GTAACTAAACTCGCAGGTCAT	T	174	GTAACTAAACCCGCAGGTCAT	C	14	0.08046	188
2906_C2_KT5786_125.spltChi.qc.fq	5865	ATTTCTAAGTCGTTCACAATC	C	583	ATTTCTAAGTTGTTCACAATC	T	5	0.00858	588
2906_C2_KT5786_125.spltChi.qc.fq	6029	AGCTTCACTAGAAGGATATTG	G	533	AGCTTCACTAAAAGGATATTG	A	3	0.00563	536
2906_C2_KT5786_125.spltChi.qc.fq	11455	GTAACTAAACTCGCAGGTCAT	T	194	GTAACTAAACCCGCAGGTCAT	C	11	0.05670	205
2906_C2_MK0364_125.spltChi.qc.fq	5865	ATTTCTAAGTCGTTCACAATC	C	2	ATTTCTAAGTTGTTCACAATC	T	377	0.00531	379
2906_C2_MK0364_125.spltChi.qc.fq	6029	AGCTTCACTAGAAGGATATTG	G	1	AGCTTCACTAAAAGGATATTG	A	357	0.00280	358
2906_C2_MK0364_125.spltChi.qc.fq	11455	GTAACTAAACTCGCAGGTCAT	T	0	GTAACTAAACCCGCAGGTCAT	C	160	0.00000	160
2906_C2_MK0365_125.spltChi.qc.fq	6029	AGCTTCACTAGAAGGATATTG	G	0	AGCTTCACTAAAAGGATATTG	A	656	0.00000	656
2906_C2_MK0365_125.spltChi.qc.fq	5865	ATTTCTAAGTCGTTCACAATC	C	1	ATTTCTAAGTTGTTCACAATC	T	618	0.00162	619
2906_C2_MK0365_125.spltChi.qc.fq	11455	GTAACTAAACTCGCAGGTCAT	T	0	GTAACTAAACCCGCAGGTCAT	C	129	0.00000	129
2906_C2_MK0366_125.spltChi.qc.fq	5865	ATTTCTAAGTCGTTCACAATC	C	1	ATTTCTAAGTTGTTCACAATC	T	563	0.00178	564
2906_C2_MK0366_125.spltChi.qc.fq	6029	AGCTTCACTAGAAGGATATTG	G	1	AGCTTCACTAAAAGGATATTG	A	499	0.00200	500
2906_C2_MK0366_125.spltChi.qc.fq	11455	GTAACTAAACTCGCAGGTCAT	T	0	GTAACTAAACCCGCAGGTCAT	C	294	0.00000	294
2906_C2_MK0366_125.spltChi.qc.fq	18301	ATAGTATTTTGATACTTGTAA	G	0	ATAGTATTTTTTATACTTGTAA	T	190	0.00000	190
2906_C2_MK0368_125.spltChi.qc.fq	5865	ATTTCTAAGTCGTTCACAATC	C	0	ATTTCTAAGTTGTTCACAATC	T	982	0.00000	982
2906_C2_MK0368_125.spltChi.qc.fq	6029	AGCTTCACTAGAAGGATATTG	G	2	AGCTTCACTAAAAGGATATTG	A	299	0.00669	301
2906_C2_MK0368_125.spltChi.qc.fq	11455	GTAACTAAACTCGCAGGTCAT	T	0	GTAACTAAACCCGCAGGTCAT	C	187	0.00000	187
3007_C1_12578_EMLH_125.spltChi.qc.fq	12395	TAATCTCAAAATTAGCAGAGG	T	0	TAATCTCAAAAATAGCAGAGG	A	3203	0.00000	3203
3007_C1_12578_EMLH_125.spltChi.qc.fq	12758	AACACTGGGGACATCCTGTGC	A	1	AACACTGGGGGCATCCTGTGC	G	1965	0.00051	1966
3007_C1_12578_EMLH_125.spltChi.qc.fq	9719	ATCCATCTCACCAAAAAATAA	C	2	ATCCATCTCATCAAAAAATAA	T	937	0.00213	939
3007_C1_12578_EMLH_125.spltChi.qc.fq	2484	TTATCATATGGTGAAGGATGA	G	2	TTATCATATGATGAAGGATGA	A	786	0.00254	788
3007_C1_12578_EMLH_125.spltChi.qc.fq	6140	CTTTTCCAAAAAACATTTTCC	A	0	CTTTTCCAAAGAACATTTTCC	G	665	0.00000	665
3007_C1_12578_EMLH_125.spltChi.qc.fq	10199	TCTGGGCTAACCCCACCAGGT	C	1	CTGGGCTAACTCCACCAGGTC	T	550	0.00182	551
3007_C1_12578_EMLH_125.spltChi.qc.fq	14415	GGAATCTAGGCGATCCAGTTA	C	0	GGAATCTAGGAGATCCAGTTA	A	150	0.00000	150
3007_C1_12579_EMLH_125.spltChi.qc.fq	12395	TAATCTCAAAATTAGCAGAGG	T	1	TAATCTCAAAAATAGCAGAGG	A	1711	0.00058	1712
3007_C1_12579_EMLH_125.spltChi.qc.fq	12758	AACACTGGGGACATCCTGTGC	A	1	AACACTGGGGGCATCCTGTGC	G	675	0.00148	676
3007_C1_12579_EMLH_125.spltChi.qc.fq	6140	CTTTTCCAAAAAACATTTTCC	A	0	CTTTTCCAAAGAACATTTTCC	G	491	0.00000	491
3007_C1_12579_EMLH_125.spltChi.qc.fq	2484	TTATCATATGGTGAAGGATGA	G	1	TTATCATATGATGAAGGATGA	A	475	0.00211	476
3007_C1_12579_EMLH_125.spltChi.qc.fq	10199	TCTGGGCTAACCCCACCAGGT	C	0	CTGGGCTAACTCCACCAGGTC	T	340	0.00000	340
3007_C1_12579_EMLH_125.spltChi.qc.fq	9719	ATCCATCTCACCAAAAAATAA	C	0	ATCCATCTCATCAAAAAATAA	T	288	0.00000	288
3007_C1_12580_EMLH_125.spltChi.qc.fq	12395	TAATCTCAAAATTAGCAGAGG	T	1	TAATCTCAAAAATAGCAGAGG	A	643	0.00156	644
3007_C1_12580_EMLH_125.spltChi.qc.fq	12758	AACACTGGGGACATCCTGTGC	A	0	AACACTGGGGGCATCCTGTGC	G	259	0.00000	259
3007_C1_12580_EMLH_125.spltChi.qc.fq	10199	TCTGGGCTAACCCCACCAGGT	C	1	CTGGGCTAACTCCACCAGGTC	T	187	0.00535	188
3007_C1_12580_EMLH_125.spltChi.qc.fq	2484	TTATCATATGGTGAAGGATGA	G	1	TTATCATATGATGAAGGATGA	A	161	0.00621	162
3007_C1_12580_EMLH_125.spltChi.qc.fq	6140	CTTTTCCAAAAAACATTTTCC	A	2	CTTTTCCAAAGAACATTTTCC	G	154	0.01299	156
3007_C1_12580_EMLH_125.spltChi.qc.fq	9719	ATCCATCTCACCAAAAAATAA	C	0	ATCCATCTCATCAAAAAATAA	T	109	0.00000	109
3007_C1_13247_EMLH_125.spltChi.qc.fq	12395	TAATCTCAAAATTAGCAGAGG	T	0	TAATCTCAAAAATAGCAGAGG	A	4178	0.00000	4178

SM_Table 6 Page 4 Minor Variant Fractions

3007_C1_13247_EMLH_125.spltChi.qc.fq	12758	AACACTGGGGACATCCTGTGC	A	0	AACACTGGGGGCATCCTGTGC	G	1045	0.00000	1045
3007_C1_13247_EMLH_125.spltChi.qc.fq	2484	TTATCATATGGTGAAGGATGA	G	1	TTATCATATGATGAAGGATGA	A	969	0.00103	970
3007_C1_13247_EMLH_125.spltChi.qc.fq	6140	CTTTTCCAAAAAACATTTTCC	A	0	CTTTTCCAAAGAACATTTTCC	G	541	0.00000	541
3007_C1_13247_EMLH_125.spltChi.qc.fq	10199	TCTGGGCTAACCCCACCAGGT	C	0	CTGGGCTAACTCCACCAGGTC	T	540	0.00000	540
3007_C1_13247_EMLH_125.spltChi.qc.fq	9719	ATCCATCTCACCAAAAAAATAA	C	0	ATCCATCTCATCAAAAAAATAA	T	441	0.00000	441
3007_C1_442_EMLH_125.spltChi.qc.fq	2484	TTATCATATGGTGAAGGATGA	G	432	TTATCATATGATGAAGGATGA	A	0	0.00000	432
3007_C1_442_EMLH_125.spltChi.qc.fq	12395	TAATCTCAAAATTAGCAGAGG	T	399	TAATCTCAAAAATAGCAGAGG	A	0	0.00000	399
3007_C1_442_EMLH_125.spltChi.qc.fq	12758	AACACTGGGGACATCCTGTGC	A	303	AACACTGGGGGCATCCTGTGC	G	1	0.00330	304
3007_C1_442_EMLH_125.spltChi.qc.fq	6140	CTTTTCCAAAAAACATTTTCC	A	253	CTTTTCCAAAGAACATTTTCC	G	0	0.00000	253
3007_C1_442_EMLH_125.spltChi.qc.fq	10199	TCTGGGCTAACCCCACCAGGT	C	253	CTGGGCTAACTCCACCAGGTC	T	0	0.00000	253
3007_C1_442_EMLH_125.spltChi.qc.fq	9719	ATCCATCTCACCAAAAAAATAA	C	110	ATCCATCTCATCAAAAAAATAA	T	0	0.00000	110
3007_C1_443_EMLH_125.spltChi.qc.fq	12395	TAATCTCAAAATTAGCAGAGG	T	1145	TAATCTCAAAAATAGCAGAGG	A	0	0.00000	1145
3007_C1_443_EMLH_125.spltChi.qc.fq	12758	AACACTGGGGACATCCTGTGC	A	790	AACACTGGGGGCATCCTGTGC	G	1	0.00127	791
3007_C1_443_EMLH_125.spltChi.qc.fq	2484	TTATCATATGGTGAAGGATGA	G	445	TTATCATATGATGAAGGATGA	A	2	0.00449	447
3007_C1_443_EMLH_125.spltChi.qc.fq	10199	TCTGGGCTAACCCCACCAGGT	C	426	CTGGGCTAACTCCACCAGGTC	T	3	0.00704	429
3007_C1_443_EMLH_125.spltChi.qc.fq	6140	CTTTTCCAAAAAACATTTTCC	A	382	CTTTTCCAAAGAACATTTTCC	G	0	0.00000	382
3007_C1_443_EMLH_125.spltChi.qc.fq	9719	ATCCATCTCACCAAAAAAATAA	C	378	ATCCATCTCATCAAAAAAATAA	T	0	0.00000	378
3007_C1_448_EMLH_125.spltChi.qc.fq	12395	TAATCTCAAAATTAGCAGAGG	T	694	TAATCTCAAAAATAGCAGAGG	A	0	0.00000	694
3007_C1_448_EMLH_125.spltChi.qc.fq	12758	AACACTGGGGACATCCTGTGC	A	318	AACACTGGGGGCATCCTGTGC	G	0	0.00000	318
3007_C1_448_EMLH_125.spltChi.qc.fq	10199	TCTGGGCTAACCCCACCAGGT	C	191	CTGGGCTAACTCCACCAGGTC	T	2	0.01047	193
3007_C1_448_EMLH_125.spltChi.qc.fq	2484	TTATCATATGGTGAAGGATGA	G	165	TTATCATATGATGAAGGATGA	A	0	0.00000	165
3007_C1_448_EMLH_125.spltChi.qc.fq	6140	CTTTTCCAAAAAACATTTTCC	A	158	CTTTTCCAAAGAACATTTTCC	G	0	0.00000	158
3007_C1_448_EMLH_125.spltChi.qc.fq	9719	ATCCATCTCACCAAAAAAATAA	C	151	ATCCATCTCATCAAAAAAATAA	T	0	0.00000	151
3007_C1_75708_EMLH_125.spltChi.qc.fq	12395	TAATCTCAAAATTAGCAGAGG	T	0	TAATCTCAAAAATAGCAGAGG	A	371	0.00000	371
3007_C1_75708_EMLH_125.spltChi.qc.fq	12758	AACACTGGGGACATCCTGTGC	A	0	AACACTGGGGGCATCCTGTGC	G	135	0.00000	135
3007_C1_75708_EMLH_125.spltChi.qc.fq	6140	CTTTTCCAAAAAACATTTTCC	A	0	CTTTTCCAAAGAACATTTTCC	G	100	0.00000	100
3007_C1_77524_EMLK_125.spltChi.qc.fq	12395	TAATCTCAAAATTAGCAGAGG	T	0	TAATCTCAAAAATAGCAGAGG	A	352	0.00000	352
3007_C1_77524_EMLK_125.spltChi.qc.fq	12758	AACACTGGGGACATCCTGTGC	A	0	AACACTGGGGGCATCCTGTGC	G	171	0.00000	171
3007_C1_77524_EMLK_125.spltChi.qc.fq	6140	CTTTTCCAAAAAACATTTTCC	A	0	CTTTTCCAAAGAACATTTTCC	G	100	0.00000	100
3007_C1_77525_EMLK_125.spltChi.qc.fq	12395	TAATCTCAAAATTAGCAGAGG	T	2	TAATCTCAAAAATAGCAGAGG	A	8431	0.00024	8433
3007_C1_77525_EMLK_125.spltChi.qc.fq	2484	TTATCATATGGTGAAGGATGA	G	10	TTATCATATGATGAAGGATGA	A	3560	0.00281	3570
3007_C1_77525_EMLK_125.spltChi.qc.fq	6140	CTTTTCCAAAAAACATTTTCC	A	0	CTTTTCCAAAGAACATTTTCC	G	1837	0.00000	1837
3007_C1_77525_EMLK_125.spltChi.qc.fq	12758	AACACTGGGGACATCCTGTGC	A	2	AACACTGGGGGCATCCTGTGC	G	1581	0.00127	1583
3007_C1_77525_EMLK_125.spltChi.qc.fq	9719	ATCCATCTCACCAAAAAAATAA	C	1	ATCCATCTCATCAAAAAAATAA	T	574	0.00174	575
3007_C1_77526_EMLK_125.spltChi.qc.fq	2484	TTATCATATGGTGAAGGATGA	G	4	TTATCATATGATGAAGGATGA	A	2240	0.00179	2244
3007_C1_77526_EMLK_125.spltChi.qc.fq	12395	TAATCTCAAAATTAGCAGAGG	T	0	TAATCTCAAAAATAGCAGAGG	A	1054	0.00000	1054
3007_C1_77526_EMLK_125.spltChi.qc.fq	10199	TCTGGGCTAACCCCACCAGGT	C	0	CTGGGCTAACTCCACCAGGTC	T	926	0.00000	926
3007_C1_77526_EMLK_125.spltChi.qc.fq	12758	AACACTGGGGACATCCTGTGC	A	2	AACACTGGGGGCATCCTGTGC	G	615	0.00325	617
3007_C1_77526_EMLK_125.spltChi.qc.fq	6140	CTTTTCCAAAAAACATTTTCC	A	0	CTTTTCCAAAGAACATTTTCC	G	512	0.00000	512
3007_C1_77526_EMLK_125.spltChi.qc.fq	9719	ATCCATCTCACCAAAAAAATAA	C	0	ATCCATCTCATCAAAAAAATAA	T	126	0.00000	126
3007_C1_77529_EMLK_125.spltChi.qc.fq	2484	TTATCATATGGTGAAGGATGA	G	2	TTATCATATGATGAAGGATGA	A	1924	0.00104	1926
3007_C1_77529_EMLK_125.spltChi.qc.fq	12395	TAATCTCAAAATTAGCAGAGG	T	0	TAATCTCAAAAATAGCAGAGG	A	1007	0.00000	1007
3007_C1_77529_EMLK_125.spltChi.qc.fq	6140	CTTTTCCAAAAAACATTTTCC	A	1	CTTTTCCAAAGAACATTTTCC	G	851	0.00118	852
3007_C1_77529_EMLK_125.spltChi.qc.fq	10199	TCTGGGCTAACCCCACCAGGT	C	1	CTGGGCTAACTCCACCAGGTC	T	756	0.00132	757
3007_C1_77529_EMLK_125.spltChi.qc.fq	12758	AACACTGGGGACATCCTGTGC	A	0	AACACTGGGGGCATCCTGTGC	G	719	0.00000	719
3007_C1_77549_EMLK_125.spltChi.qc.fq	12395	TAATCTCAAAATTAGCAGAGG	T	0	TAATCTCAAAAATAGCAGAGG	A	141	0.00000	141
3007_C1_77551_EMLK_125.spltChi.qc.fq	12395	TAATCTCAAAATTAGCAGAGG	T	0	TAATCTCAAAAATAGCAGAGG	A	555	0.00000	555
3007_C1_77551_EMLK_125.spltChi.qc.fq	12758	AACACTGGGGACATCCTGTGC	A	0	AACACTGGGGGCATCCTGTGC	G	247	0.00000	247
3007_C1_77551_EMLK_125.spltChi.qc.fq	6140	CTTTTCCAAAAAACATTTTCC	A	0	CTTTTCCAAAGAACATTTTCC	G	188	0.00000	188
3007_C1_77551_EMLK_125.spltChi.qc.fq	2484	TTATCATATGGTGAAGGATGA	G	1	TTATCATATGATGAAGGATGA	A	117	0.00855	118
3007_C1_77551_EMLK_125.spltChi.qc.fq	10199	TCTGGGCTAACCCCACCAGGT	C	0	CTGGGCTAACTCCACCAGGTC	T	112	0.00000	112
3007_C1_77558_EMLK_125.spltChi.qc.fq	12395	TAATCTCAAAATTAGCAGAGG	T	0	TAATCTCAAAAATAGCAGAGG	A	813	0.00000	813
3007_C1_77558_EMLK_125.spltChi.qc.fq	12758	AACACTGGGGACATCCTGTGC	A	0	AACACTGGGGGCATCCTGTGC	G	303	0.00000	303
3007_C1_77558_EMLK_125.spltChi.qc.fq	10199	TCTGGGCTAACCCCACCAGGT	C	0	CTGGGCTAACTCCACCAGGTC	T	259	0.00000	259
3007_C1_77558_EMLK_125.spltChi.qc.fq	2484	TTATCATATGGTGAAGGATGA	G	2	TTATCATATGATGAAGGATGA	A	226	0.00885	228
3007_C1_77558_EMLK_125.spltChi.qc.fq	6140	CTTTTCCAAAAAACATTTTCC	A	1	CTTTTCCAAAGAACATTTTCC	G	220	0.00455	221
3007_C1_77558_EMLK_125.spltChi.qc.fq	9719	ATCCATCTCACCAAAAAAATAA	C	0	ATCCATCTCATCAAAAAAATAA	T	148	0.00000	148
3007_C1_77563_EMLK_125.spltChi.qc.fq	12395	TAATCTCAAAATTAGCAGAGG	T	0	TAATCTCAAAAATAGCAGAGG	A	1466	0.00000	1466
3007_C1_77563_EMLK_125.spltChi.qc.fq	12758	AACACTGGGGACATCCTGTGC	A	1	AACACTGGGGGCATCCTGTGC	G	498	0.00201	499
3007_C1_77563_EMLK_125.spltChi.qc.fq	6140	CTTTTCCAAAAAACATTTTCC	A	0	CTTTTCCAAAGAACATTTTCC	G	430	0.00000	430
3007_C1_77563_EMLK_125.spltChi.qc.fq	2484	TTATCATATGGTGAAGGATGA	G	0	TTATCATATGATGAAGGATGA	A	338	0.00000	338
3007_C1_77563_EMLK_125.spltChi.qc.fq	10199	TCTGGGCTAACCCCACCAGGT	C	1	CTGGGCTAACTCCACCAGGTC	T	337	0.00297	338
3007_C1_77563_EMLK_125.spltChi.qc.fq	9719	ATCCATCTCACCAAAAAAATAA	C	0	ATCCATCTCATCAAAAAAATAA	T	237	0.00000	237
3007_C1_77572_EMLK_125.spltChi.qc.fq	12395	TAATCTCAAAATTAGCAGAGG	T	0	TAATCTCAAAAATAGCAGAGG	A	429	0.00000	429
3007_C1_77572_EMLK_125.spltChi.qc.fq	12758	AACACTGGGGACATCCTGTGC	A	0	AACACTGGGGGCATCCTGTGC	G	179	0.00000	179
3007_C1_77572_EMLK_125.spltChi.qc.fq	10199	TCTGGGCTAACCCCACCAGGT	C	0	CTGGGCTAACTCCACCAGGTC	T	117	0.00000	117
3007_C1_77572_EMLK_125.spltChi.qc.fq	6140	CTTTTCCAAAAAACATTTTCC	A	0	CTTTTCCAAAGAACATTTTCC	G	100	0.00000	100
3007_C1_KT7095_125.spltChi.qc.fq	12395	TAATCTCAAAATTAGCAGAGG	T	0	TAATCTCAAAAATAGCAGAGG	A	817	0.00000	817
3007_C1_KT7095_125.spltChi.qc.fq	12758	AACACTGGGGACATCCTGTGC	A	1	AACACTGGGGGCATCCTGTGC	G	319	0.00313	320
3007_C1_KT7095_125.spltChi.qc.fq	6140	CTTTTCCAAAAAACATTTTCC	A	0	CTTTTCCAAAGAACATTTTCC	G	213	0.00000	213
3007_C1_KT7095_125.spltChi.qc.fq	10199	TCTGGGCTAACCCCACCAGGT	C	1	CTGGGCTAACTCCACCAGGTC	T	157	0.00637	158
3007_C1_KT7095_125.spltChi.qc.fq	2484	TTATCATATGGTGAAGGATGA	G	0	TTATCATATGATGAAGGATGA	A	138	0.00000	138
3007_C1_KT7095_125.spltChi.qc.fq	9719	ATCCATCTCACCAAAAAAATAA	C	0	ATCCATCTCATCAAAAAAATAA	T	112	0.00000	112
3007_C1_MK9396_125.spltChi.qc.fq	12395	TAATCTCAAAATTAGCAGAGG	T	1	TAATCTCAAAAATAGCAGAGG	A	4662	0.00021	4663
3007_C1_MK9396_125.spltChi.qc.fq	2484	TTATCATATGGTGAAGGATGA	G	4	TTATCATATGATGAAGGATGA	A	1708	0.00234	1712
3007_C1_MK9396_125.spltChi.qc.fq	12758	AACACTGGGGACATCCTGTGC	A	1	AACACTGGGGGCATCCTGTGC	G	1004	0.00100	1005
3007_C1_MK9396_125.spltChi.qc.fq	6140	CTTTTCCAAAAAACATTTTCC	A	0	CTTTTCCAAAGAACATTTTCC	G	914	0.00000	914
3007_C1_MK9396_125.spltChi.qc.fq	10199	TCTGGGCTAACCCCACCAGGT	C	0	CTGGGCTAACTCCACCAGGTC	T	658	0.00000	658
3007_C1_MK9396_125.spltChi.qc.fq	9719	ATCCATCTCACCAAAAAAATAA	C	0	ATCCATCTCATCAAAAAAATAA	T	375	0.00000	375
