# Supplementary material for: Rapid outbreak sequencing of Ebola virus in Sierra Leone identifies transmission chains linked to sporadic cases
Source: Virus Evol. 2016 Jun 22;2(1):vew016. doi: 10.1093/ve/vew016 (PMC5499387; doi:10.1093/ve/vew016)
Supplement: Supplementary Data [file vew016_Supp_01.zip › Supplementary_Material_8May16_plus_figures.pdf]

**Supplementary Material for Outbreak_sequencing_of_Ebola_virus**

**Short read data handling and genome assembly.** Index specific bam files were generated by Ion Torrent software and were converted to fastq format using a combination of SAMtools[1] and awk[2]. Reads were trimmed and quality filtered using QUASR[3]. Nucleotides were removed from the 3' end until a median Phred score >30 was obtained, terminal primers starting within the terminal 5 nts were removed and all resulting reads with length <125 nt were discarded. Chimeric reads were then resolved using a Python script (available at https://github.com/simonjwatson/chimeric-splitter). In brief, the script took a FASTQ file of the short reads and performed a BLAST search with each read against an EBOV Makona database. If a read returned multiple hits against different regions of the genome, the read was split and the subfragments were collected and combined with all non-chimeric reads in a clean fastq file. The final reads sets were the processed by *de novo* assembly using SPAdes 3.5.0[4] with Ion Torrent settings, EBOV contigs were identified using ublast[5] with an EBOV protein database and further assembled into complete genomes (if not already a complete genome) using Sequencher v5.3 (Gene Codes Corporation, USA). Conflicts were resolved by direct counting of the motif in the short read data set. The provisional consensus genome was compared to a close reference genome and all nucleotide differences were checked and the majority nucleotide at that position was determined by directly counting the 21nt motif spanning the nucleotide (10nt of either side of the SNP) in the quality-controlled short read set for that sample.

SM_Figure1

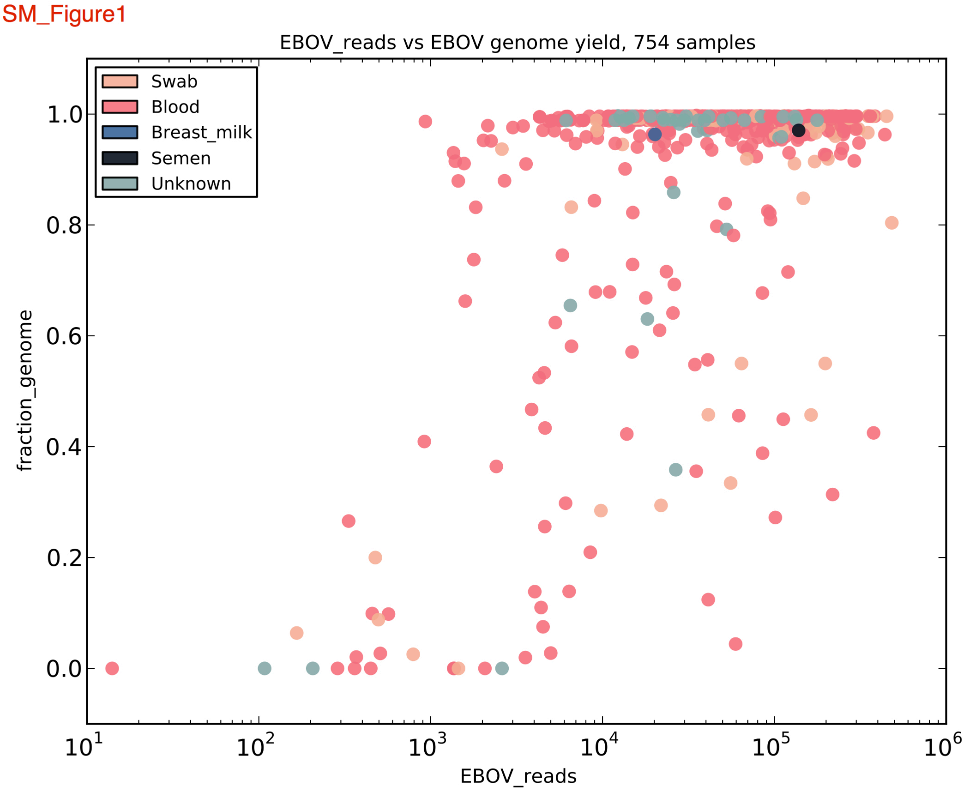


**SM_Figure 1 Genome yield.** EBOV full genome yields were plotted as a function of EBOV read content. The short read sets for the 554 EBOV samples yielding complete genomes plus an additional 200 samples that did not yield complete genomes were examined. Each point represents a sample plotted by the number of EBOV short reads present in the sample (determined by Bowtie2[6] mapping with " --very-sensitive-local" settings to Zaire Ebola virus KM034562) and the obtained fraction of the virus genome (total length of contig/18957). Sample types (blood, swab, breast milk, semen or unknown) are indicated at the top left. Obtaining a complete viral genome was associated with higher levels of EBOV short reads (a surrogate for the number of RNA genome copies per ml in the original sample) with genome yields dropping in samples containing less than 10,000 reads.

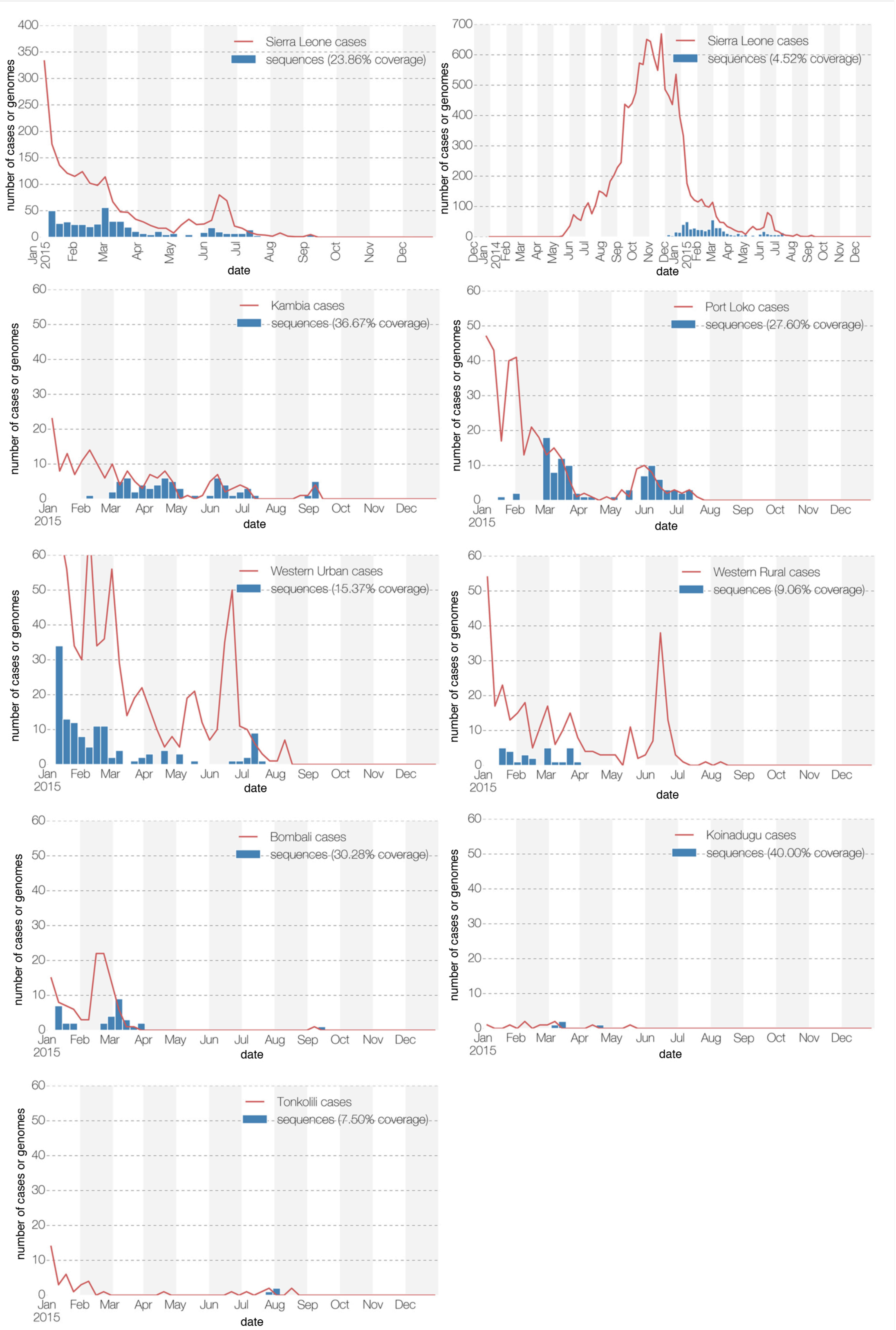


**SM_Figure 2 EBOV Genome sampling coverage in Sierra Leone**. Red lines show the sum of confirmed and suspected EVD cases for each epi week, as reported by WHO[7]. Blue bars indicate the number full EBOV genomes that were generated in this study from that week's samples. The inset value within each panel indicates the percentage of cases for which sequences are available within a given location for the time period shown in each plot.

SM_Figure 3

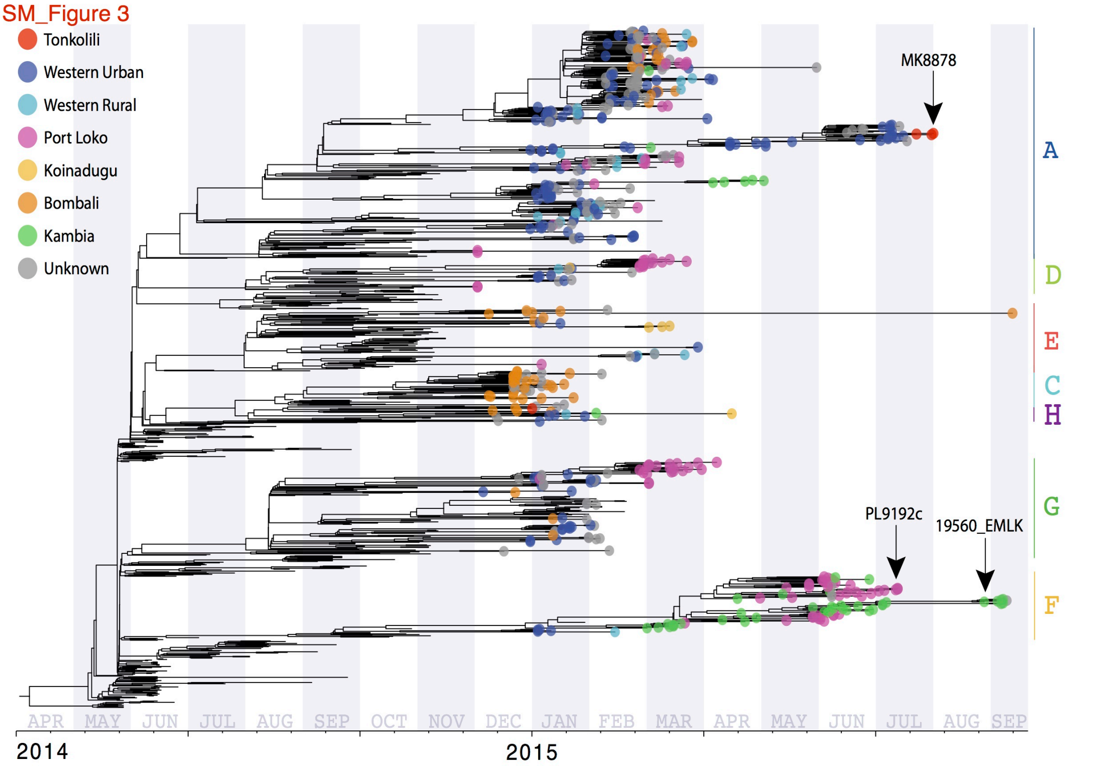


**SM_Figure 3 Time scaled tree.** A time-scaled phylogenetic tree was generated from an alignment of 1058 Sierra Leone virus genomes from the SL3 introduction into Sierra Leone, excluding those viruses within the SL3 lineage that were derived from a re-introduction from another country. The lineages defined in Figure 2 are shown to the right of the tree. Lineage B is not shown as it derived from a cross-border introduction from Guinea, while the GUI-1 viruses were descended from a separate introduction from Guinea into Sierra Leone to that of the SL3 lineage. Viruses sequenced in this study are shown as circles on the tips, coloured by the district they were collected from. A legend for the district colours is given at the top-left. The phylogenetic location of the three transmission chain vignettes are marked with arrows, labeled with a representative virus from each vignette (PL9192 from the Mamusa cluster, MK8878 from the Tonkolili cluster and 19560_EMLK from the Kambia cluster)

SM_Figure 4

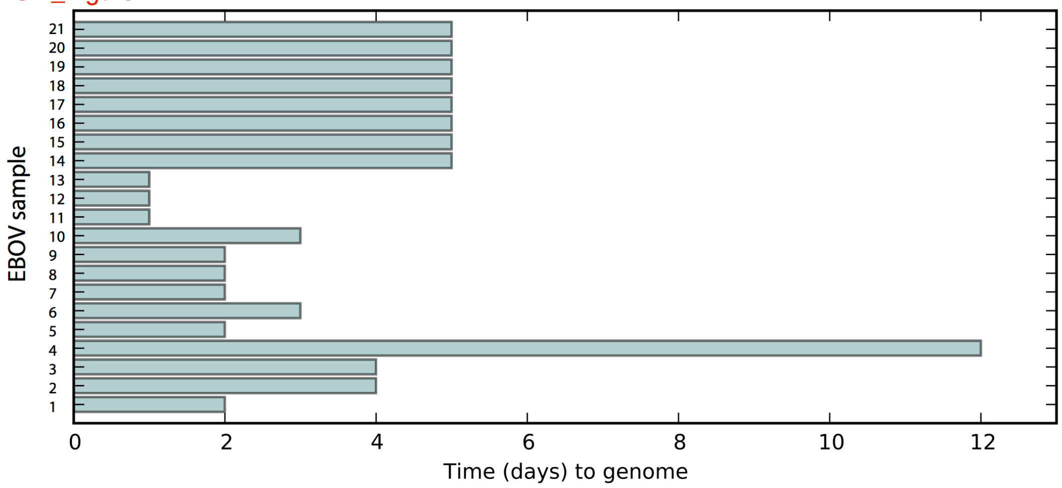


**SM_Figure 4 EOSS Response time.** 21 EVD samples (listed as Samples 1-21, Y-axis) were processed as part of the Ebola Outbreak Sequencing Support. Elapsed time (in days) from sample receipt at the sequencing tent to finished EBOV genome is plotted.

**SM_Table 1 Metadata 554 genomes.** For each of the 554 genomes, the table lists the GenBank accession number, the sample collection date, the sample type, the geographical location of case (at the district level), the number of short reads obtained from the sample, the median read length for the raw sequencing data and a mean sequencing coverage value for the assembled genome determined by Bowtie2[6] mapping with " --very-sensitive-local" settings.

**SM_Table 2 Primer and their binding positions.** The primers are organized by amplicon with forward (FWD_PRIMER), and reverse primer (REV_PRIMER) sequences listed, the pool in which the primers were used (Pool1 or Pool2) and the beginning (Position1) and ending (Position2) positions of the resulting amplicon in the EBOV reference genome (GenBank accession number KM034562).

**Comparison of duplicate sequencing on Ion Torrent.** A measure of the accuracy of the generated genomes was obtained by examining the genomes from 28 samples processed with two independent Ion Torrent runs. A comparison of the metrics for the 28 runs is provided in **SM_Table 3**. In the 28 pairs of genomes, there were 49 positions showing differences, 47 of these differences were due to insertion/deletion in one of the pair, with only 2 positions showing nucleotide differences. **SM_Table 3** lists the genome IDs for samples that were sequenced on two independent Ion Torrent runs. The table columns show the sample id (Sample), the genome_1 ID

(genome_1), the GenBank accession number for genome_1 (GenBank), the genome_2 ID (genome_2), the number of differences in an alignment of the two sequences, (differences), the number of those differences due to nucleotide differences (nt_diff) or insertion/deletions (indel).

**MinION/Ion Torrent comparison.** As part of the EOSS, 7 EBOV samples were sequenced on both the Ion Torrent platform in the Sierra Leone facility and the Oxford Nanopore MinION platform in Coyah, Guinea[8]; data available at ENA: http://www.ebi.ac.uk/ena/data/view/ERP011834]. Five of the MinION sequences yielded sufficient coverage for comparison. Comparing the resulting consensus genomes from the two methods, there were a total of 20 position differences between the 5 pairs of genomes. Importantly, there were no nucleotide differences between any of the 5 pairs of assembled genomes. All detected differences were due to insertion/deletions (indels) in Ion Torrent data and uncalled bases (Ns) in MinION data, which is expected due to the different sample preparation method and bioinformatics analysis employed. Crucially, neither indels nor the Ns would contribute to the phylogenetic inference from the genomes as these columns are masked from alignments before analysis, hence would not lead to phylogenetic inconsistencies or alteration of inferred transmission chains. The metrics of the comparison are provided in **SM_Table 4 Minion Ion Torrent comparison**.

**Residual_primers.** Primer sequence carry-over from the reverse transcription/PCR amplification could influence the resulting consensus sequences and the following two measures are included in the sequencing and genome assembly process to limit this effect:

A. During the viral RNA reverse transcription and PCR amplification process, modified primers are used that are susceptible to enzymatic digestions and are removed from the amplicons before ligation to the library adapters. These methods are described in detail in the patent publication (U.S. Publication No. 20120295819, U.S. Patent No. 8,673,560).

B. Because of the overlapping amplicon design, two categories of primer sequences will occur in the short read data. Terminal primers attached during the process of reverse transcription and PCR amplification and internal primers derived from the actual amplification of viral sequence (see Figure below). Terminal sequences have the potential to introduce nucleotide changes if there is a mismatch between the primer and the target that does not disrupt primer binding during the amplification. During the quality control processing of the raw reads and before *de novo* assembly,

a primer trimming algorithm is applied to remove primer sequences identified at the termini of reads but to retain those sequences if they appear internal to the read. The process further minimizes primer carry through influencing the resulting consensus sequence.

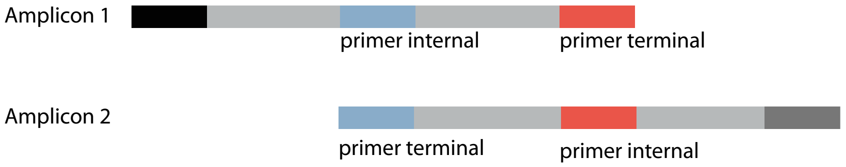


To demonstrate the extent of the primer removal process, metrics on the residual terminal primer content of all reads sets for the 554 genomes were determined.

The metrics of residual primers are summarized below:

| | |
|---|---|
| Average number of reads per sample | 163,222 |
| Average fraction of terminal primers remaining | 0.00030 |
| Average number terminal primers remaining per sample | 49.42 |
| 95% confidence interval | $4.7922 \times 10^{-5}$ |
| Terminal primers remaining 95%CI lower limit | 41.59 |
| Terminal primers remaining 95%CI upper limit | 57.24 |

Due to their low frequency in the read sets, these primer-bearing reads are unlikely to be incorporated into the *de novo* assembled viral contigs and are not expected to influence the derived consensus sequences. The actual metrics of residual primers present in each of the 554 short read sets is shown in **SM_Table 5 Residual primers**. The table show the Genome id (Genome_ID), the total number of quality-controlled reads available for that sample (number_reads), the number of reads examined in the analysis (reads_examined), the average read length (average_read_length), the number of reads with primer seqeunces identified at the the 5' or 3' end (no_reads_primer_ends) and the fraction of the the total reads this represents (fraction).

**Cross Sample Contamination.** It was important to assess the potential level of

cross sample contamination in the sequencing process. Contaminants could influence the conclusions. Good laboratory hygiene and workflow were established in the sequencing tent to minimize the contamination risks, including separate working areas with designated handling UV-hoods for template addition and for PCR clean area. Extensive between-run decontamination was performed with nucleic acid removal solutions, disposal of all waste and UV inactivation of reagent and sample handling hoods. Additionally, the Ion Torrent system has the advantage that all library preparation supplies including vessels, reagents, pipette tips, columns and peripheral plasticware are provided in individually packaged, run-sized units. This greatly limits run-to-run carryover of amplified material. A selection of the 64 available barcodes were used in sequential order such that consecutive library preps did not use overlapping barcode. As only barcoded data is included in the assembly, any potential contaminant would most likely have to originate from samples prepared several days (or weeks) previously.

We were very concerned about contamination of one set of reads with reads from another sample. Such inter-sample contamination would appear when the major nucleotide variant in sample A appears as a minor variant in sample B. Such cross-contamination would compromise the use of minor variants information to track virus transmission.

This minor nucleotide content could be due to natural variation in the virus population and we show in several of the close transmission chains that minor variants can provide additional information about transmission. Therefore, such an analysis requires that other sources of the minor nucleotide can be excluded. Such sources could be contamination from samples containing this nucleotide as a major variant, machine error in identifying this nucleotide at this position and enzymatic errors in the reverse transcription and/or PCR amplification process. As a method of assessing the background level of nucleotide variation we developed the following computational test for such contamination.

We have developed a measure of the level of inter-sample cross-contamination taking advantage of our sampling of viruses from distinct dates and locations, ie. epidemiologically distinct viruses. Our rationale was to identify genome positions that distinguish two such groups of Ebola virus samples which show no variation in the consensus genomes across the group. Because there was no variation observed at that site within the subgroup, it is unlikely that minor variants exist in any of the

subgroup samples due to natural viral evolution. Thus the appearance of a minor non-consensus nucleotide at such ‘sentinel positions’ could be due to machine noise, enzymatic errors or contamination from another sample processed at the same time. We then identified sequencing runs that contained at least two epidemiologically distinct groups; the frequency of a minor variants at these sentinel positions provides a measure of the potential contamination.

We identified 4 Ion Torrent runs that contained at least two distinct viral populations with a total of 354 sentinel nucleotide positions. Only positions with coverage ≥100 were included. Examples of 2 sentinel positions in an Ion Torrent run are presented in the following figure. Within this set, all 11 samples had been run on a single Ion Torrent run (0208_C1). The 9 EMLK samples were from the EMLabs, Kambia diagnostic lab and had been collected in January 2015 from the Western Urban district, the two MK samples were from the PHE Makeni diagnostic lab and had been collected in the Tonkolili district in July 2015. Genome positions 5046 and 10511 were distinct between the two groups but showed no intragroup variation.

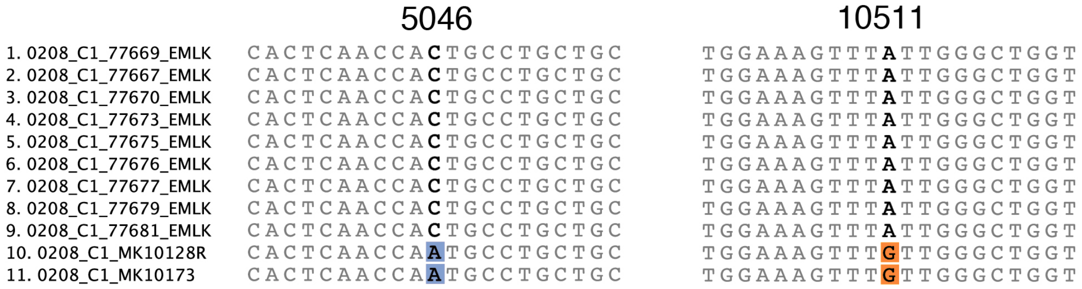


For each of these 354 sentinel positions we calculated the fraction of non-consensus nucleotides at that position. Because both variants were present as a majority species in at least two samples processed during that sequencing run, yet the variant is not observed in the majority species within that subgroup. Thus the fraction of minority variant at each of these positions is a measure of potential inter-sample contamination occurring during the actual sample processing and sequencing runs in the sequencing facility in Sierra Leone. The complete metrics for all 354 sentinel sites are presented in **ED_Table 6 Minor Variant fractions** and the summary values are listed below.

| | | |
|---|---|---|
| average read counts | 900.13 | |
| mean minor variant fraction | 0.0038 | 0.38% |
| 95% confidence interval | 0.0017 | 0.17% |
| lower fraction | 0.0021 | 0.21% |
| upper fraction | 0.0055 | 0.55% |

We found that for positions with sequencing coverage ≥100 the potential contamination fraction is 0.38% (95%CI: 0.21 to 0.55). Based on these data, we are confident that minor variants frequencies >1% reflect true viral variants in the sample. For the positions analyzed in **Figure 5b**, the minor variants present at ≥1% frequency are marked with a red asterisk.

## References


1 Li, H. *et al.* The Sequence Alignment/Map format and SAMtools. *Bioinformatics* **25**, 2078-2079, doi:10.1093/bioinformatics/btp352 (2009).

2 Turner, S. Useful bash one-liners for bioinformatics. . *https://github.com/stephenturner/oneliners* (2016).

3 Watson, S. J. *et al.* Viral population analysis and minority-variant detection using short read next-generation sequencing. *Philos Trans R Soc Lond B Biol Sci* **368**, 20120205, doi:10.1098/rstb.2012.0205
rstb.2012.0205 [pii] (2013).

4 Bankevich, A. *et al.* SPAdes: a new genome assembly algorithm and its applications to single-cell sequencing. *J Comput Biol* **19**, 455-477, doi:10.1089/cmb.2012.0021 (2012).

5 Edgar, R. C. Search and clustering orders of magnitude faster than BLAST. *Bioinformatics* **26**, 2460-2461, doi:10.1093/bioinformatics/btq461 (2010).

6 Langmead, B. & Salzberg, S. L. Fast gapped-read alignment with Bowtie 2. *Nature methods* **9**, 357-359, doi:10.1038/nmeth.1923 (2012).

7 WHO. Ebola data and statistics. *http://apps.who.int/gho/data/node.ebola-sitrep.ebola-country?lang=en* (2016).

8 Quick, J. *et al.* Real-time, portable genome sequencing for Ebola surveillance. *Nature* **530**, 228-232, doi:10.1038/nature16996 (2016).
